# Proteomic Analysis of Mesenchymal Stem Cells and Monocyte Co-Cultures Exposed to a Bioactive Silica-Based Sol–Gel Coating

**DOI:** 10.1021/acsbiomaterials.3c00254

**Published:** 2023-05-19

**Authors:** Andreia Cerqueira, Francisco Romero-Gavilán, Heike Helmholz, Mikel Azkargorta, Félix Elortza, Mariló Gurruchaga, Isabel Goñi, Regine Willumeit-Römer, Julio Suay

**Affiliations:** † Department of Industrial Systems Engineering and Design, Universitat Jaume I, Av. Vicent Sos Baynat s/n, 12071 Castellón de la Plana, Spain; ‡ Helmholtz-Zentrum Hereon Institute of Metallic Biomaterials, Max-Planck-St.1, Geesthacht D-21502, Germany; § Proteomics Platform, Basque Research and Technology Alliance (BRTA), CIBERehd, Bizkaia Science and Technology Park, CIC bioGUNE, 48160 Derio, Spain; ∥ Department of Science and Technology of Polymers, University of the Basque Country, P. M. de Lardizábal, 3, 20018 San Sebastián, Spain

**Keywords:** biomaterials, osteoimmunology, co-cultures, proteomics, sol−gel coatings

## Abstract

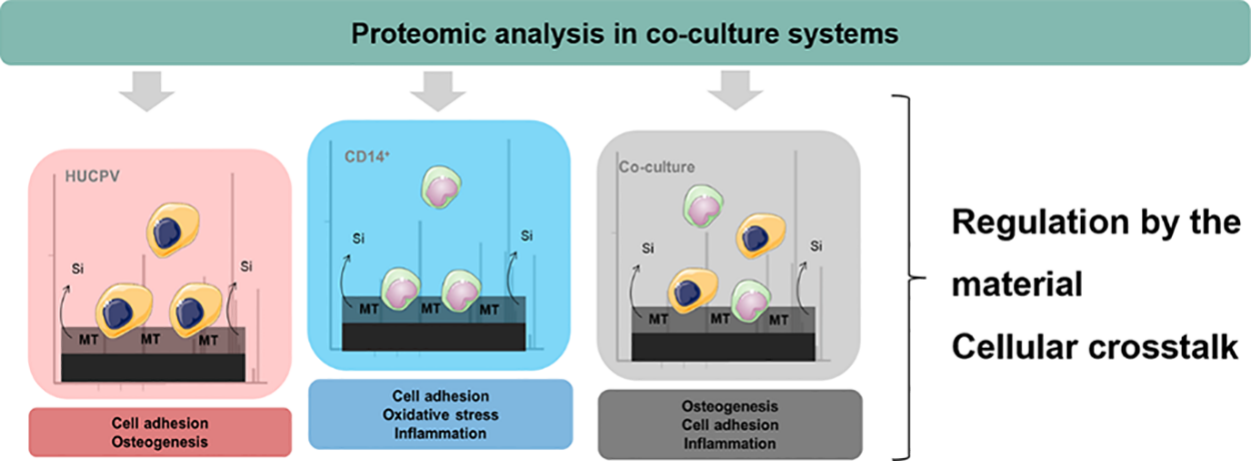

New methodologies capable of extensively analyzing the cell-material interactions are necessary to improve current in vitro characterization methods, and proteomics is a viable alternative. Also, many studies are focused on monocultures, even though co-cultures model better the natural tissue. For instance, human mesenchymal stem cells (MSCs) modulate immune responses and promote bone repair through interaction with other cell types. Here, label-free liquid chromatography tandem mass spectroscopy proteomic methods were applied for the first time to characterize HUCPV (MSC) and CD14^+^ monocytes co-cultures exposed to a bioactive sol–gel coating (MT). PANTHER, DAVID, and STRING were employed for data integration. Fluorescence microscopy, enzyme-linked immunosorbent assay, and ALP activity were measured for further characterization. Regarding the HUCPV response, MT mainly affected cell adhesion by decreasing integrins, RHOC, and CAD13 expression. In contrast, MT augmented CD14^+^ cell areas and integrins, Rho family GTPases, actins, myosins, and 14-3-3 expression. Also, anti-inflammatory (APOE, LEG9, LEG3, and LEG1) and antioxidant (peroxiredoxins, GSTO1, GPX1, GSHR, CATA, and SODM) proteins were overexpressed. On co-cultures, collagens (CO5A1, CO3A1, CO6A1, CO6A2, CO1A2, CO1A1, and CO6A3), cell adhesion, and pro-inflammatory proteins were downregulated. Thus, cell adhesion appears to be mainly regulated by the material, while inflammation is impacted by both cellular cross-talk and the material. Altogether, we conclude that applied proteomic approaches show its potential in biomaterial characterization, even in complex systems.

## 1 Introduction

Osteoimmunology is an interdisciplinary field that studies the mechanisms and interactions between bone and immune cells.^1^ These two systems share numerous cytokines, receptors, signaling molecules, and transcription factors, and the dynamic cross-talk between them is critical for bone tissue formation and healing.^2^ Upon material implantation, inflammation is the first response, and monocytes/macrophages are key factors in the host response to the foreign body.^2^ These cells release important molecules responsible for the recruitment of mesenchymal stem cells (MSCs) capable of modulating immune responses and promoting bone tissue repair.^3^ With this, the new generations of biomaterials should be able to modulate the local immune environment in a way that favors osteogenesis and material osseointegration.^4^

The lack of comprehension of the biomaterial-biological system interaction has presented a major barrier to the development of effective biomaterials.^5^ On the one hand, the characterization of biomaterials has focused on monoculture studies, even though the use of complex co-culture systems presents a better model of the natural tissues, physically and biologically, through interactions between different cell types.^6^ On the other hand, the difficulties in translating in vitro results into in vivo outcomes have increased the need to develop alternative assays that able us of overcoming the current inadequacies of present characterization methods.^7^ Considering this, proteomics offers a potent tool to describe cell behavior on material surfaces.^8^ Recently, these approaches have been employed to study the effects of biomaterials in osteosarcoma,^9^ MSCs,^10^ and human osteoblasts.^11,12^ However, the number of studies is still quite limited and no studies analyzing the protein expression patterns on co-culture systems could be found to date.

Currently, metallic devices, such as titanium (Ti) and alloys, represent the gold-standard treatments for bone defects.^13^ These materials provide a specific surface for protein and cell attachment, which undoubtedly guides implant/prosthesis fate, and must be analyzed as a whole.^14^ However, considering the biologically inert nature of these surfaces, modifications are necessary to increase their bioactivity. Hybrid silica sol–gel coatings are biocompatible, biodegradable, and capable of releasing silicate ions, leading to positive effects in the proliferation, differentiation, and osteogenic gene expression of osteoblasts.^15^ Considering this, our group has developed a sol–gel coating employing 70% methyltrimethoxysilane (M) and 30% tetraethyl orthosilicate (T) as precursors, and we showed that this surface modification improves in vitro osteogenic response and Ti in vivo osseointegration properties.^16^

Here, we present the first study characterizing protein expression profiles of human umbilical cord perivascular cells (HUCPV; source of MSC) and CD14^+^ monocyte co-cultures exposed to a hybrid silica sol–gel coating (MT). The proteomic profiles of cells exposed to uncoated Ti and MT-coated Ti were compared. Protein identification was carried out with liquid chromatography tandem mass spectrometry (LC–MS/MS), and the expression regulation was analyzed with computational methods. With this, we aim to further elucidate the biological response to these materials in a more complex setting.

## 2 Materials and Methods

### 2.1 Material Synthesis

The sol–gel route was applied to obtain coatings for titanium (Ti) surfaces. The precursors selected were methyltrimethoxysilane (M; Merck, Darmstadt, Germany) and tetraethyl orthosilicate (T; Merck) in a molar ratio of 7:3. The sol–gel synthesis was carried out as described by Araújo-Gomes et al.^17^ Then, grade-4 sandblasted and acid-etched titanium discs (12 mm diameter, 1 mm thick) were used as a substrate for the coatings. The discs were immersed in the sol–gel solutions at 60 cm min^–1^ for 1 min and removed at 100 cm min^–1^ with a dip-coater (KSV DC; KSV NIMA, Espoo, Finland). The process was carried out at room temperature (25 °C).

### 2.2 Cell Isolation and Culture

Human umbilical cord perivascular (HUCPV) cells were isolated from the perivascular site (Wharton’s jelly) of human umbilical cords, as described in the study of Wang et al.^18^ The material was provided by the Bethesda Hospital Hamburg Bergedorf, and the utilization of human donor materials was approved by the Ethic Commission of the Hamburg Medical Association. Cells were expanded in α-Minimum Essential Medium (MEM; Sigma-Aldrich, Munich, Germany) supplemented with 15% (v/v) fetal bovine serum for human mesenchymal stem cells (SC-FBS; Biological Industries, Beit-Haemek, Israel) and 1% (v/v) penicillin (100 U/mL)/streptomycin (100 μg/mL) (pen/strep; ThermoFisher Scientific GmbH, Schwerte, Germany).

Human leukocyte-enriched blood samples were obtained with leukoreduction chambers used during platelet (thrombocyte) donation. Blood from six healthy donors was provided by University Hospital Hamburg-Eppendorf (UKE; Hamburg, Germany). Anticoagulant solution (2.13% ACD-A citrate dextrose solution) was added at the time of donation. Peripheral blood mononuclear cells (PBMCs) were obtained by density gradient separation, as described by Wang et al.^18^ Then, the CD14^+^ monocytes were isolated from PBMCs employing the anti-human CD14 M-pluriBead kit (pluriSelect Life Science, Leipzig, Germany) and following the manufacturer’s instructions. The cell culture medium was composed of Roswell Park Memorial Institute (RPMI) 1640 Medium (Sigma-Aldrich), 10% (v/v) heat-inactivated FBS (Biochrom, Berlin, Germany), 1% pen/strep, 2 mM glutamate, and 20 ng mL^–1^ macrophage colony-stimulating factor (Sigma-Aldrich).

Cell cultures were maintained under physiological conditions (5% CO_2_, 20% O_2_, 95% relative humidity, 37 °C).

### 2.3 Co-Culture Systems

For direct co-culture systems, 24-well plates were coated with 1% (v/v) agarose (Sigma-Aldrich) before the experiment to avoid cell adherence on the plate surface rather than on the material surface. The materials were sterilized under ultraviolet (UV) light for 30 min before being placed on the agarose-coated wells with sterile tweezers. HUCPV and CD14^+^ cells alone were seeded onto the materials at a concentration of 1 × 10^4^ and 6 × 10^4^ cells per cm^–2^, respectively. In co-cultures, the same concentrations were applied. The rationale followed for these concentrations (1:6) was based on the studies by Wang et al.^3,18^ The co-culture medium was composed of α-MEM, 15% FBS, and 1% pen/strep. The cultures were maintained for 7 and 14 days, and the medium was changed every 2–3 days.

### 2.4 Cytoskeleton Arrangement

F-actin staining was employed to evaluate cytoskeleton arrangement. Cells were fixed with 4% paraformaldehyde (PFA; Alfa Aesar, ThermoFisher Scientific GmbH) for 20 min at room temperature. Then, cells were permeabilized with 0.1% Triton X-100 in PBS. Actin was stained with tetramethylrhodamine (TRITC)-conjugated phalloidin (1:500, Millipore, Sigma-Aldrich), and nuclei were stained with DAPI (5 μg/mL, Millipore). Samples were immediately analyzed with an Eclipse Ti-S microscope and NIS-Elements Microscope Imaging Software (Nikon GmbH, Düsseldorf, Germany, version 4.51).

### 2.5 Cytokine Release and ALP Activity

For cytokine release, the cell culture supernatants were collected after 7 and 14 days in culture, centrifuged, and stored at −80 °C until further analysis. Interleukin (IL)-10 and IL-1β concentrations were quantified with enzyme-linked immunosorbent assay kits (ELISA; R&D Systems GmbH, Wiesbaden, Germany) according to the manufacturer’s protocol. The absorbance was measured at 450 nm in a microplate reader (Tecan Sunrise; TECAN Deutschland GmbH, Crailsheim, Germany).

Alkaline phosphatase (ALP) activity was measured based on the hydrolyzation of *p*-nitrophenyl phosphate (*p*-NPP) onto *p*-nitrophenol. The Quantichrom alkaline phosphatase assay kit (BioAssay Systems, Hayward, CA, USA) was employed to quantify ALP activity following the manufacturer’s protocol. The absorbance was measured at 405 nm with a microplate reader.

### 2.6 Proteomic Analysis: Protein Extraction, Identification, and Functional Classification

For protein extraction, at each time point, samples were washed thrice with PBS, and the cells were lysed with RIPA buffer (G-Biosciences, St. Louis, MO, USA) with protease inhibitors, followed by incubation on ice for 15 min. Then, the lysate was collected and frozen at −80 °C until further analysis. Four technical replicates consisting of a pool of 3 discs were extracted.

For protein digestion, to every 70 μL of the sample, twice as much extraction buffer (7 M urea, 2 M thiourea, 4% 3-[(3-cholamidopropyl)dimethylammonio]-1-propanesulfonate (CHAPS), and 200 mM dithiothreitol (DTT)) was added. Then, the filter-aided sample preparation (FASP) protocol as described by Wiśniewski et al.^19^ was applied for tryptic digestion. After reduction and alkylation, 1:50 trypsin/protein ratio was used, and the mixture was incubated overnight at 37 °C. The resulting peptides were dried out in an RVC2 25 speedvac concentrator (Christ, Osterode/Harz, Germany) and resuspended in 0.1% formic acid (FA). Peptides were desalted using C18 stage tips (Merk Millipore, Burlington, MA) and resuspended in 0.1% FA.

For protein identification, 200 ng of purified and 0.1% FA resuspended sample were loaded into an Evosep One chromatograph (Evosep Biosystems, Odense C, Denmark) coupled to a hybrid trapped ion mobility quadrupole time-of-flight mass spectrometer (timsTOF Pro with PASEF; Bruker, Billerica, MA). The Evosep 30 SPD protocol was applied (44 min gradient) and a 15 cm column (Evosep) was used. The timsTOF Pro was operated in data-dependent acquisition (DDA) mode using the Standard 1.1 s cycle time acquisition mode. The mass spectrum raw dataset was examined using the MaxQuant (http://maxquant.org/), and label-free comparative analysis was done with Perseus (https://www.maxquant.org/perseus/). Only proteins identified with at least two different peptides at 1% false discovery rate (FDR) (peptide-spectrum match (PSM)-level) were considered in the analysis. Relative label-free quantification (LFQ) intensities were used for the quantitative analysis of proteins. Samples were analyzed in quadruplicate.

For functional classification, PANTHER (http://www.pantherdb.org/), DAVID (https://david.ncifcrf.gov/), and UniProt (https://www.uniprot.org/) were employed. Oliveros, J.C. (2007–2015) Venny. An interactive tool for comparing lists with Venn’s diagrams (https://bioinfogp.cnb.csic.es/tools/venny/index.html) was used to compare protein lists and make Venn diagrams. Protein–protein interactions were analyzed using the Search Tool for the Retrieval of Interacting Genes/Proteins (STRING; https://string-db.org, accessed on January 23, 2023) database of physical and functional interactions v11.5. The selected settings were full string network, meaning of network edge by evidence, involvement of all active interaction sources, and high confidence (0.7). Network nodes represent proteins and edges represent protein–protein associations. Protein clustering was done via *K*-means clustering (*K* = 3), also employing STRING.

### 2.7 Statistical Analysis

For in vitro assay data, considering normal distribution and equal variance, a one-way variance analysis (ANOVA) with Tukey post hoc test was done to evaluate differences between MT, Ti, and monocultures/co-cultures. A Student’s *t*-test was performed to confirm the results. GraphPad Prism 5.04 software (GraphPad Software Inc., La Jolla, CA, USA) was employed for the statistical analysis and the differences were considered significant at *p* ≤ 0.05 (*), *p* ≤ 0.01 (**), and *p* ≤ 0.001 (***). Data were expressed as mean ± standard deviation (SD).

To determine which proteins were differentially expressed on MT in relation to Ti, the Student’s *t*-test was conducted with Perseus. Protein expression was considered statistically significant when *p* ≤ 0.05, and the ratio difference was higher than 1.5 in either direction (under or overexpressed).

## 3 Results

### 3.1 Fluorescence Microscopy, Cytokine Release, and ALP Activity

**Figure 1:**
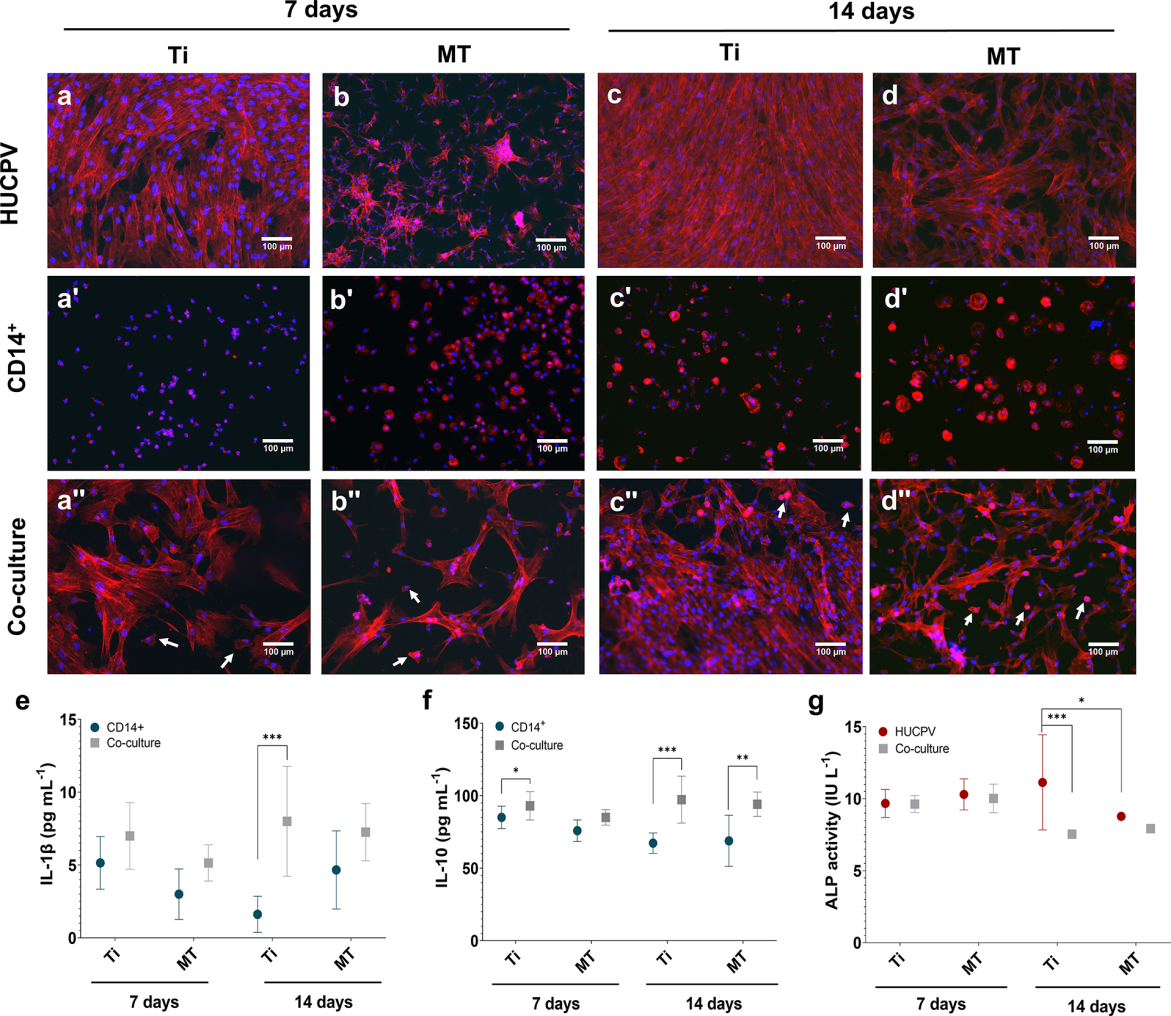
Microscopic fluorescence images of cytoskeleton arrangement for HUCPV, CD14^+^, and co-culture on Ti (a–a” and c–c”) and MT (b–b” and d–d”) at 7 and 14 days. Actin filaments were stained with phalloidin (red) and nuclei with DAPI (blue). Arrows in co-cultures indicate the monocytes. Scale bar: 100 μm. Interleukin (IL)-10 (e) and IL-1β (f) release in CD14^+^ and co-cultures at 7 and 14 days. Alkaline phosphate (ALP) activity (g) in HUCPV and co-cultures at 7 and 14 days. Results are shown as mean ± SD. The asterisks (*p* ≤ 0.05 (*), *p* ≤ 0.01 (**), and *p* ≤ 0.001 (***)) indicate statistically significant differences between monocultures, co-cultures, and materials (Ti and MT).

To study the cytoskeleton arrangement of HUCPV, CD14^+^, and co-cultures on the materials, cells were stained with fluorescence-conjugated phalloidin at 7 and 14 days (Figure 1a–d”). HUCPV cells cultured on Ti showed an elongated shape and formed a uniform layer on the surface at both time points (Figure 1a,c). On MT, these cells presented a triangular shape, being more dispersed on the material surface (Figure 1b,d). The CD14^+^ cells seeded on Ti displayed a barely visible cytoskeleton, while on MT, the cells showed a rounded shape (Figure 1a’,b’). At 14 days, the cells both on Ti and MT showed an increment in the cell area, being particularly predominant on MT (Figure 1c’,d’). Finally, on co-cultures, HUCPV cells showed a triangular shape both on Ti and MT, while CD14^+^ cells presented a lower cell area (indicated by the arrows).

Interleukin (IL)-1β and IL-10 concentrations were measured in cell culture supernatants to evaluate the inflammatory potential of the materials (Figure 1e,f). Regarding IL-1β, no differences were found between materials and cell culture systems (CD14^+^ and co-culture) at 7 days (Figure 1e). After 14 days, only the co-culture system showed a significant increase in relation to CD14^+^ cells on Ti. On the other hand, Ti led to a significant increase in IL-10 production in the co-culture at 7 days (Figure 1f). After 14 days, both co-cultures seeded on Ti and MT presented a significantly higher concentration of IL-10 compared to the CD14^+^ monoculture.

ALP activity was measured on HUCPV and co-culture systems to measure the osteogenic capability of the materials (Figure 1g). No differences were found after 7 days of culture regardless of the culture system (mono or co-culture) and the material. After 14 days, the co-culture showed a significantly lower ALP activity on Ti, regarding HUCPV cells. Similarly, HUCPV cells seeded on MT also showed a significantly lower enzymatic activity when compared to the same culture on Ti.

### 3.2 Proteomic Analysis

A total of 817 proteins were found to be significantly regulated by MT in HUCPV, CD14^+^, and co-culture systems (Table S1). Table 1 summarizes the number of proteins found up or downregulated by the material at 7 and 14 days.

**Table 1:** Number of Proteins Up and Downregulated in HUCPV, CD14^+^, and Co-Culture Systems at 7 and 14 Days on MT Compared with Untreated Ti As Determined by LC–MS/MS

	HUCPV		CD14^+^		co-culture	
	7 days	14 days	7 days	14 days	7 days	14 days
up	10	20	38	558	6	15
down	59	67	6	5	775	137
total	69	87	46	563	781	152

In HUCPV cells, MT regulated a total of 69 proteins, 10 upregulated and 59 downregulated; at 14 days, 87 were differentially expressed, with 20 being upregulated and 67 downregulated. Regarding CD14^+^ cells, MT significantly regulated 46 proteins after 7 days of culture (38 upregulated and 6 downregulated). After 14 days, a total of 563 proteins were affected by MT coating, with 558 being upregulated and 5 downregulated. In the co-culture systems, a total of 781 proteins were identified that were significantly regulated by the material after 7 days of culture wherein 6 presented a higher expression and 775 were downregulated. At 14 days of culture, 152 proteins were identified, with 15 being upregulated and 137 being downregulated.

#### 3.2.1 Monoculture Proteomic Profile: HUCPV and CD14^+^

**Figure 2:**
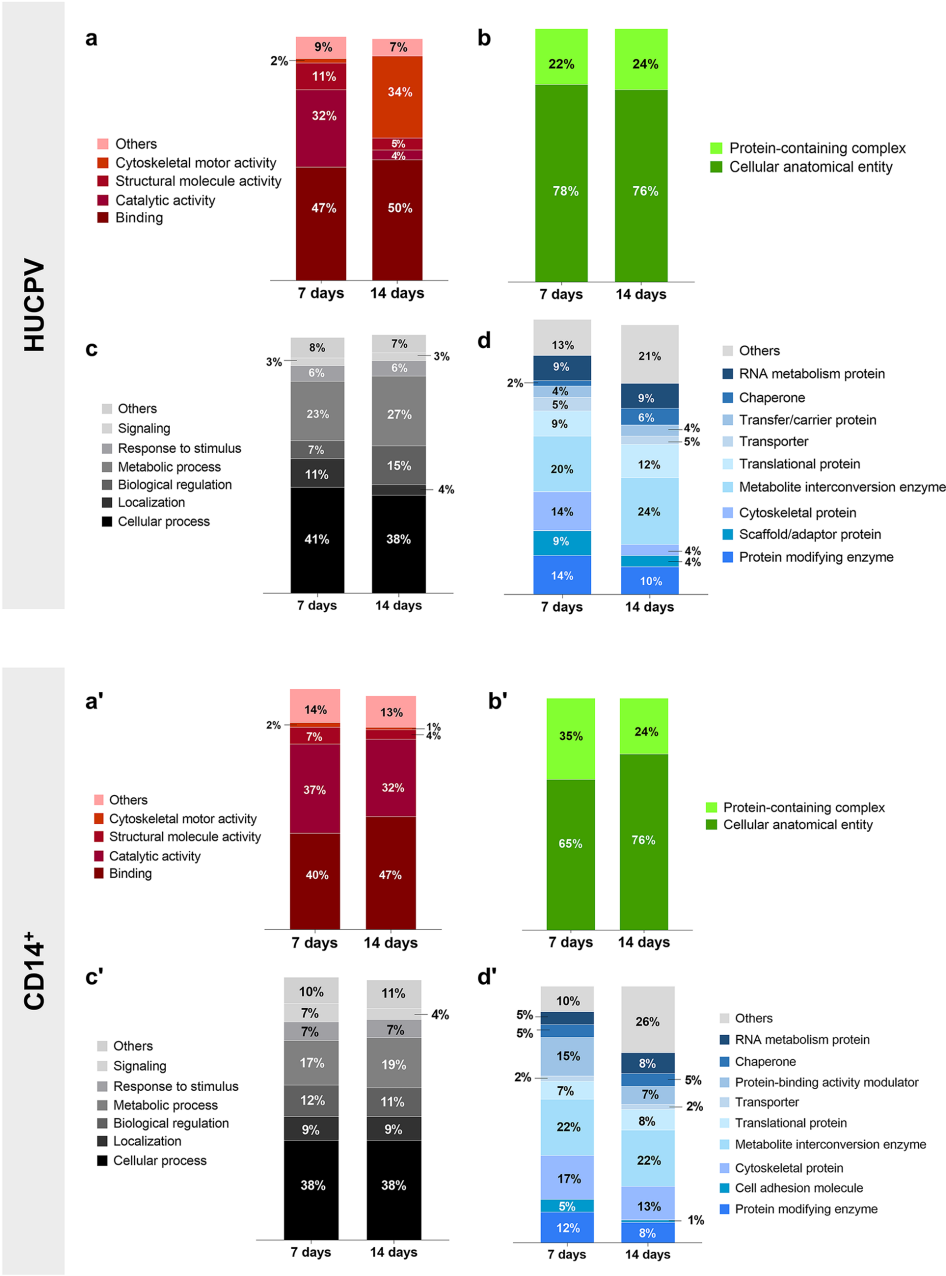
PANTHER analysis of the molecular function (a and a’), cellular component (b and b’), biological process (c and c’), and protein class (d and d’) of the proteins differentially expressed by HUCPV and CD14^+^ cells seeded onto MT at 7 and 14 days.

PANTHER analysis was used to associate the proteins differentially expressed in HUCPV and CD14^+^ cells with their molecular function, biological component, cellular component, and protein class (Figure 2). On HUCPV cells, MT mainly regulated the expression of proteins associated with binding, catalytic activity, structural molecule activity, and cytoskeletal motor activity (Figure 2a) located either in a cellular anatomical entity or in a protein-containing complex (Figure 2b). In what concerns biological processes, the identified proteins were associated with cellular processes (41 to 42%), while the others participate in processes of localization, biological regulation, metabolic process, response to stimulus, and signaling (Figure 2c). The main protein classes identified were the protein modifying enzyme, scaffold/adaptor protein, cytoskeletal protein, metabolite interconversion enzyme, transporter, transfer/carrier protein, chaperone, and RNA metabolism protein (Figure 2d).

On CD14^+^ cells, MT led to the expression of proteins mainly associated with binding, catalytic activity, structural molecule activity, and cytoskeletal motor activity (Figure 2a’) associated with the cellular anatomical entity (65 to 76%) or in a protein-containing complex (35 to 24%) (Figure 2b’). The biological processes identified were mostly cellular processes, localization, biological regulation, metabolic process, response to stimulus, and signaling (Figure 2c’). The protein classes identified were protein modifying enzyme, cell adhesion molecule, cytoskeleton protein, metabolite interconversion enzyme, translational protein, transporter, protein-binding activity modulator, chaperon, and RNA metabolism protein (Figure 2d’).

Table 2 lists all the pathways upregulated and downregulated on HUCPV and CD14^+^ cells exposed to MT after 7 and 14 days. On HUCPV cells, after 7 days of culture, MT regulated three pathways associated with cell adhesion (cytoskeleton regulation by Rho GTPase, Ras pathway, integrin signaling) and angiogenesis. Similarly, MT also affected proteins associated with cell adhesion (Ras pathway, integrin signaling, cadherin signaling), angiogenic responses (angiogenesis, VEGF signaling), and osteogenesis (JAK/STAT signaling, Wnt signaling) after 14 days.

**Table 2:** PANTHER Analysis of the Pathways Up and Downregulated on HUCPV and CD14^+^ Cells Seeded on MT Compared with Untreated Ti As Determined by LC–MS/MS after 7 and 14 Days

	7 days	14 days
HUCPV		
↑ up	Ras pathway	JAK/STAT signaling
	angiogenesis	PDGF signaling
	cytoskeletal regulation by Rho GTPase	EGF receptor signaling
		Ras pathway
		angiogenesis
↓ down	integrin signaling	cadherin signaling
	angiogenesis	integrin signaling
	FGF signaling	Wnt signaling
	CCKR signaling map	VEGF signaling
	EGF receptor signaling	CCKR signaling map
	cytoskeletal regulation by Rho GTPase	FAS signaling
CD14^+^		
↑ up	JAK/STAT signaling	angiogenesis
	angiogenesis	CCKR signaling map
	integrin signaling	Wnt signaling
	EGF receptor signaling	VEGF signaling
	PI3 kinase pathway	toll receptor signaling
	PDGF signaling	T cell activation
	cytoskeletal regulation by Rho GTPase	TGF-β signaling
	Ras pathway	PI3 kinase pathway
	cadherin signaling	PDGF signaling
	CCKR signaling map	oxidative stress response
	Wnt signaling	JAK/STAT signaling
	apoptosis signaling	interleukin signaling
		interferon-γ signaling
		integrin signaling
		insulin/IGF pathway-MAPK cascade
		inflammation by chemokine and cytokine signaling
		Ras pathway
		p38 MAPK
		FGF signaling
		FAS signaling
		endothelin signaling
		EGF receptor signaling
		cytoskeletal regulation by Rho GTPase
		cadherin signaling
		B cell activation
↓ down	—	—

On CD14^+^ cells, no pathways were downregulated at both time points. On the other hand, after 7 days, pathways associated with inflammatory responses (inflammation by chemokine and cytokine, interleukin signaling), cell adhesion (integrin signaling, cadherin signaling, cytoskeleton regulation by Rho GTPase, Ras signaling), and angiogenesis (angiogenesis, PDGF signaling) were significantly more expressed. At 14 days, processes associated with cell adhesion (integrin signaling, cadherin signaling, Ras signaling), inflammation (T cell activation, TGF-β signaling, interleukin signaling, interferon-ϒ signaling, inflammation by chemokine and cytokine, B cell activation), cell survival regulation (PI3 kinase, Wnt signaling), and oxidative stress were upregulated.

Table S1 shows the proteins that were differentially expressed by HUCPV and CD14^+^ cells at 7 and 14 days of culture. Considering the pathways identified, the proteins regulated on HUCPV cells by MT were associated with cell adhesion, oxidative stress, osteogenesis, and angiogenesis (Table 3). In what concerns cell adhesion, inverted formin-2 (INF2) and annexin A7 (ANXA7) were upregulated after 7 days, while 17 proteins (S10AB, PBIP1, PUR9, IPO9, CAD13, ILK, IPO7, RHOC, DBNL, G3BP2, FINC, GBP1, CAZA2, ACTBL, ARC1B, PLST, RHG01) were downregulated. After 14 days, MT upregulated rootletin (CROCC), glial fibrillary acidic protein (GFAP), CD63 antigen (CD63), and alpha-actinin-4 (ACTN4) and downregulated three integrins (ITA11, ITA2, ITB5), one cadherin (CAD13), adseverin (ADSV), and 11 other proteins (COTL1, CNN3, TBB8, LMO7, EPHA2, HDGF, RRAS, PDLI2, PLXB2, MFGM, SYNE1) associated with cellular adhesion processes. Moreover, oxidative stress proteins were significantly upregulated by MT at both time points. Nonetheless, at 14 days, cathepsin D (CATD), major prion protein (PRIO), and cathepsin H (CATH) were downregulated. Regarding osteogenesis, peroxisomal multifunctional enzyme type 2 (DHB4) was upregulated at 7 days of culture, while plastin-3 (PLST) and collagen alpha-1(XII) chain (COCA1) were downregulated. After 14 days, heterogeneous nuclear ribonucleoproteins C1/C2 (HNRPC) and protein-glutamine gamma-glutamyltransferase 2 (TGM2) were more expressed by HUCPV exposed to MT, and four (FKB10, EPHA2, M4K4, TIMP3) were significantly less expressed. Concerning angiogenesis, after 7 days, hemoglobin subunit alpha (HBA) was upregulated, and tissue factor pathway inhibitor (TFPI2) was downregulated. At 14 days, just hemoglobin subunit beta (HBB) was significantly upregulated.

**Table 3:** Proteins Differentially Expressed by HUCPV Cultured on MT (Compared with Ti) at 7 and 14 Days with Functions on Cell Adhesion, Oxidative Stress, Osteogenesis, and Angiogenesis^a^

function		7 days	14 days
cell adhesion	↑ up	INF2, ANXA7	CROCC, GFAP, CD63, ACTN4
	↓ down	S10AB, PBIP1, PUR9, IPO9, CAD13, ILK, IPO7, RHOC, DBNL, G3BP2, FINC, GBP1, CAZA2, ACTBL, ARC1B, PLST, RHG01	ITA11, COTL1, CNN3, ITA2, CAD13, ADSV, TBB8, ITB5, LMO7, EPHA2, HDGF, RRAS, PDLI2, PLXB2, MFGM, SYNE1
oxidative stress	↑ up	—	—
	↓ down	—	CATD, PRIO, CATH
osteogenesis	↑ up	DHB4	HNRPC, TGM2
	↓ down	PLST, COCA1	FKB10, EPHA2, M4K4, TIMP3
angiogenesis	↑ up	HBA	HBB
	↓ down	TFPI2	—

a The proteins shown present *P* ≤ 0.05 and a ratio >1.5 in either direction (UP, increased and DOWN, reduced).

Screening CD14^+^ cells as monoculture, the regulated proteins were associated with cell adhesion, oxidative stress, inflammation, osteogenesis, and angiogenesis (Table 4). After 7 days, several proteins with roles on cell adhesion were upregulated by MT exposure, including integrin α-5 (ITA5), annexin A5 (ANXA5), 14-3-3 proteins (1433Z, 1433 T), actin/actinin (ACTB, ACTN1), and six others (S10AB, S10A7, TBB5, SEPT2, TPM1, FSCN1). At 14 days, 108 proteins were significantly more expressed; on the other hand, only drebrin-like protein (DBNL) was downregulated. In what concerns oxidative stress, just peroxidasin homolog (PXDN) was affected by MT after 7 days of culture. However, at 14 days, glutathione S-transferases (GSTO1, GSTP1), thioredoxin-dependent peroxide reductases (PRDX3, PRDX4, PRDX1, PRDX6), catalase (CATA), superoxide dismutase (SODM), and eight others (GPX1, GSHR, COX41, COX2, QCR1, TRXR1, CY24B, QCR2, CISY, ERO1A, TMX1) were upregulated. On inflammation, no proteins were significantly affected by MT after 7 days of culture; however, five cathepsins (CATS, CATB, CATZ, CATH, CATD), three galectins (LEG9, LEG3, LEG1), two apolipoproteins (APOE, APOL2), three HLA class I histocompatibility antigens (HLAB, HLAC, HLAB), and 19 others (DRA, PHB, BAP31, C5AR1, CHIT1, CD44, HM13, LSP1, PKHO2, KPCD, NCF2, DDX21, CD276, CH3L1, CD166, CD36, HXK1, MIF, LXN) were upregulated after 14 days. Similarly, no proteins related to osteogenic responses were regulated after 7 days of culture, but 10 proteins (DHB4, CLH1, LMNA, EIF3E, MK01, VIME, P4K2A, CPPED, RACK1, PRS7) were upregulated after 14 days. Finally, HBA was more expressed after 7 days of culture, while heme oxygenase 1 (HMOX1), myoferlin (MYOF), serpin B6 (SPB6), and heme oxygenase 2 (HMOX2) were upregulated after 14 days of exposure to MT.

**Table 4:** Proteins Differentially Expressed by CD14^+^ Cultured on MT (Compared with Ti) at 7 and 14 Days with Functions on Cell Adhesion, Oxidative Stress, Inflammation, Osteogenesis, and Angiogenesis^a^

function		7 days	14 days
cell adhesion	↑ up	S10AB, S10A7, ITA5, TBB5, SEPT2, ANXA5, ACTB, 1433Z, TPM1, ACTN1, FSCN1, 1433 T	URP2, ITB2, FLNA, ITAM, ARPC5, RAC2, RAB7A, ARC1B, ARCP2, PLEC, CYFP1, WDR1, CAPG, 1433Z, CAZA1, ITAX, ANXA4, RAB14, TYOBP, ARP3, TLN1, S10A4, ARPC4, RTN4, GPNMB, RHOG, ZYX, ARP2, RAB5C, 1433G, SNX2, ACTBL, PTN6, CAZA2, GDIR1, IQGA1, 1433E, AHNK, RAB18, CAPZB, MMP9, SDCB1, SNX5, TWF2, ANX11, RAB6A, BASI, 1433F, CDC42, TBA1A, TBB4B, MYH9, 1433B, ACTN4, RAB2A, SNX1, SEPT9, PTMA, 1433 T, ACTN1, ANXA1, MYL6, MYO1B, COF1, COTL1, RB11B, RAB1A, ANXA5, NIBA2, COR1B, RAP2B, RAP1B, ITB5, COR1C, MOES, GELS, RAB10, TBB2A, TES, RHOC, RAB1B, ACTC, RAB13, ANXA2, ML12A, RAB8A, ITB1, ITA11, PLXB2, VINC, TPM4, MYOF1, G3BP1, RAB31, EFN4, S10AB, GDIR2, PROF1, CYRIB, E41L3, EVL, ARL8B, IQGA2, CD9, LAMC1, PDLI4, PAK2, LASP1
	↓ down	—	DBNL
oxidative stress	↑ up	PXDN	GSTO1, PRDX3, GPX1, GSHR, SODM, PRDX4, PRDX1, CATA, PRDX6, COX41, COX2, QCR1, TRXR1, CY24B, QCR2, CISY, ERO1A, TMX1, GSTP1
	↓ down	—	—
inflammation	↑ up	—	CATS, LEG3, CATB, HLAB, DRA, PHB, HLAA, HLAC, BAP31, LEG9, C5AR1, APOE, CHIT1, CD44, CATZ, HM13, LSP1, PKHO2, KPCD, NCF2, CATH, LEG1, CATD, DDX21, CD276, CH3L1, CD166, CD36, HXK1, APOL2, MIF, LXN
	↓ down	—	—
osteogenesis	↑ up	—	DHB4, CLH1, LMNA, EIF3E, MK01, VIME, P4K2A, CPPED, RACK1, PRS7
	↓ down	—	—
angiogenesis	↑ up	HBA	HMOX1, MYOF, SPB6, HMOX2
	↓ down	—	—

a The proteins shown present *P* ≤ 0.05 and a ratio >1.5 in either direction (UP, increased and DOWN, reduced).

#### 3.2.2 Co-Culture System Proteomic Profile

**Figure 3:**
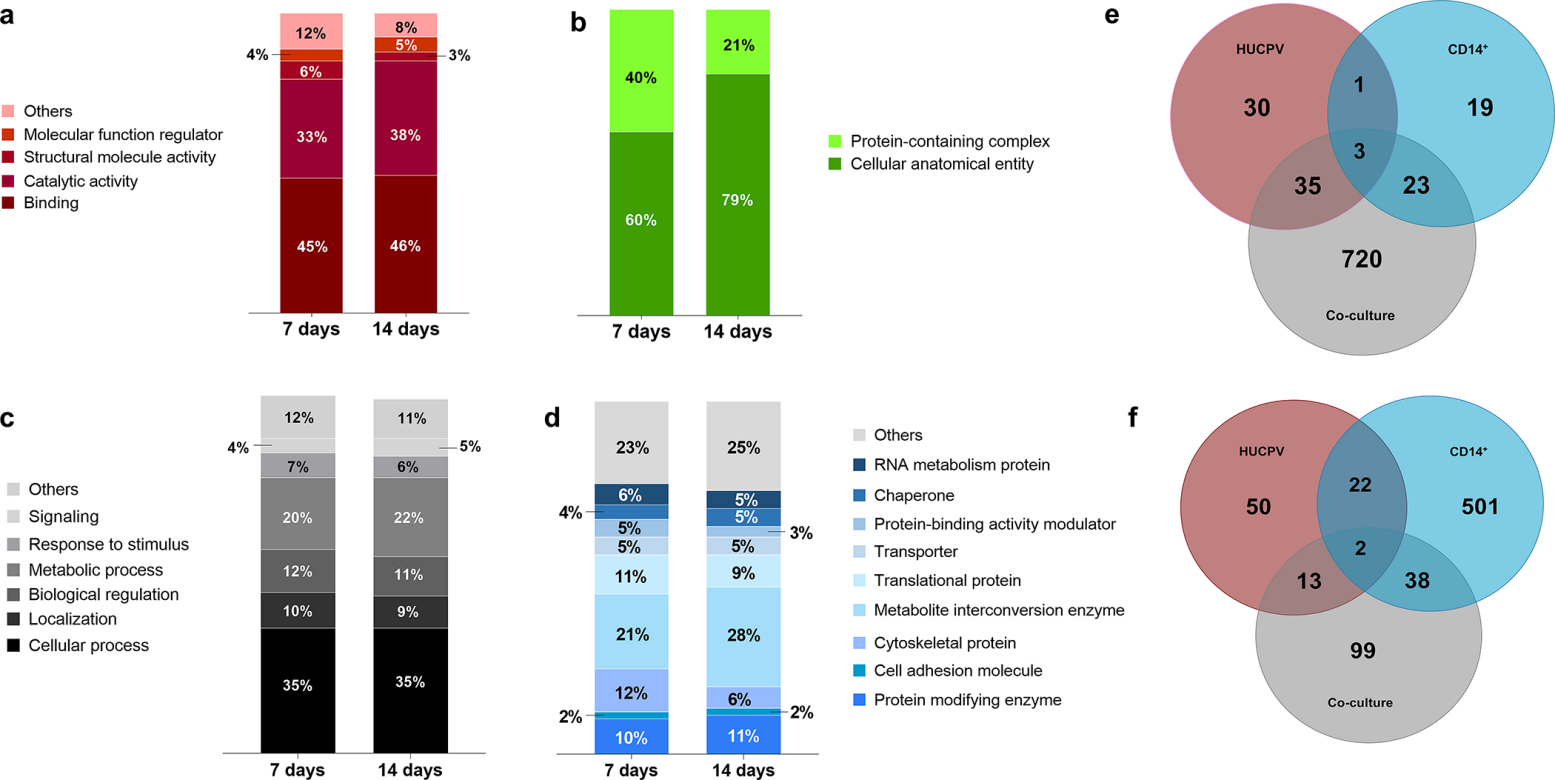
PANTHER analysis to the molecular function (a), cellular component (b), biological processes (c), and protein class (d) of the proteins differentially expressed on co-cultures seeded onto MT at 7 and 14 days. Venn diagrams of proteins differentially expressed on HUCPV, CD14^+^, and co-cultures at (e) 7 and (f) 14 days.

PANTHER analysis showed that in co-culture systems, MT mainly regulated the expression of proteins associated with binding, catalytic activity, structural molecule activity, and molecular function regulators (Figure 3a) belonging to two components (cellular anatomical entity and protein-containing complex) (Figure 3b). The main biological processes identified were cellular processes, localization, biological regulation, metabolic process, response to stimulus, and signaling (Figure 3c). The protein classes identified were protein modifying enzymes, cell adhesion molecules, cytoskeletal proteins, metabolite interconversion enzymes, translational proteins, transporters, protein-binding activity modulators, chaperones, and RNA metabolism proteins (Figure 3d).

To verify the effects of the co-cultures on the protein number in relation to monocultures cultures, Venn diagrams were made (Figure 3e,f). These show that 720 proteins were uniquely expressed by the cells maintained in the co-culture systems at 7 days, while 35 and 23 were coincident between HUCPV, CD14^+^, and co-cultures, respectively. In addition, only three proteins were coincident between all conditions (Figure 3e). At 14 days, 99 proteins were exclusively expressed by cells obtained from the co-cultures, 13 and 38 were coincident between monocultures and co-cultures, respectively, and two between the three conditions (Figure 3f).

Table 5 lists the pathways upregulated and downregulated by co-cultures exposed to MT after 7 and 14 days. PANTHER analysis showed that while MT upregulated Wnt and cadherin signaling at 7 days, no pathways were significantly upregulated after 14 days of the assay. In contrast, pathways of cell adhesion (cadherin signaling, cytoskeletal regulation by Rho GTPase, integrin signaling), inflammation (T cell activation, inflammation by chemokine and cytokine signaling, B cell activation), angiogenesis (angiogenesis, VEGF signaling, PDGF signaling), osteogenesis (PI3 kinase, Wnt signaling), and oxidative stress were downregulated after 7 days of culture. After 14 days, a similar pathway regulation pattern was found. Cadherin signaling, cytoskeletal regulation by Rho GTPase, integrin signaling, and Ras pathway were downregulated as well as T cell activation, inflammation by chemokine and cytokine signaling, B cell activation, interleukin signaling, and TGF-β signaling. Moreover, pathways associated with angiogenesis (angiogenesis, VEGF signaling, PDGF signaling) and osteogenesis (PI3 kinase, Wnt signaling, insulin/IGF pathway, vitamin D metabolism) were also downregulated.

**Table 5:** PANTHER Analysis to the Pathways Upregulated and Downregulated on Co-Cultures Seeded after Seeding on MT Compared with Untreated Ti As Determined by LC–MS/MS after 7 and 14 Days

	7 days	14 days
↑ up	Wnt signaling	—
	cadherin signaling	
↓ down	Wnt signaling	JAK/STAT signaling
	cadherin signaling	angiogenesis
	angiogenesis	interleukin signaling
	CCKR signaling map	integrin signaling
	VEGF signaling	insulin/IGF pathway-MAPK cascade
	T cell activation	inflammation mediated by chemokine and cytokine signaling
	PI3 kinase pathway	Wnt signaling
	PDGF signaling	vitamin D metabolism
	oxidative stress response	VEGF signaling
	integrin signaling	toll receptor signaling
	inflammation by chemokine and cytokine signaling	Ras pathway
	Ras pathway	T cell activation
	FGF signaling	FGF signaling
	FAS signaling	TGF-β signaling
	EGF receptor signaling	FAS signaling
	cytoskeletal regulation by Rho GTPase	endothelin signaling
	B cell activation	EGF receptor signaling
		cytoskeletal regulation by Rho GTPase
		PDGF signaling
		cadherin signaling
		B cell activation
		CCKR signaling map

Table S1 lists the proteins that were differentially expressed on co-culture systems at 7 and 14 days of culture. The proteins regulated by MT were mainly associated with cell adhesion, oxidative stress, inflammation, osteogenesis, and angiogenesis (Table 6). After 7 days, TRIO and F-actin-binding protein (TARA), which have been associated with cell adhesion, were upregulated. However, 119 proteins were significantly less expressed. On 14 days, only coactosin-like protein (COTL1) was upregulated, while 36 were downregulated. In what concerns oxidative stress, no proteins were upregulated at 7 days, while SODM, CATA, five thioredoxin-dependent peroxide reductases (PRDX5, PRDX3, PRDX2, PRDX1, PRDX6), and 12 others (NCF1C, GSTM2, GSTP1, TXND5, GSTK1, THIO, GSTO1, PXL2A, GSTM3, GPX8, TRXR1) were less expressed. On 14 days, peroxiredoxin-2 (PRDX2) and glutathione peroxidase 1 (GPX1) were upregulated, and neutrophil cytosol factor 4 (NCF4) and 3-mercaptopyruvate sulfurtransferase (THTM) were downregulated. On inflammation, no proteins were found upregulated after 7 days, while 32 were downregulated. After 14 days, fatty acid-binding protein 5 (FABP5) and cytochrome c oxidase subunit 4 isoform 1 (COX41) were upregulated. On the other hand, cathepsin Z (CATZ), endoplasmic reticulum aminopeptidase 1 (ERAP1), α-taxilin (TXLNA), interleukin enhancer-binding factor 2 (ILF2) APOE, prostaglandin reductase 1 (PTGR1), neutrophil cytosol factor 2 (NCF2), and platelet-activating factor acetylhydrolase IB subunit alpha2 (PA1B2) were significantly less expressed. Concerning osteogenesis, 25 proteins were downregulated after 7 days of culture, while no upregulated proteins were found. On 14 days, procollagen-lysine, 2-oxoglutarate 5-dioxygenase 2 (PLOD2), eukaryotic translation initiation factor 3 subunit K (EIF3K), and insulin-degrading enzyme (IDE) were more expressed. In contrast, three collagens (CO1A1, CO6A3, CO6A1), insulin-like growth factor 2 (IF2B1), reticulocalbin-3 (RCN3), cartilage-associated protein (CRTAP), and five others (P3H1, SULF1, RFIP5, HDGF, CCN1) were downregulated. Finally, in what concerns angiogenesis, no proteins were significantly more expressed after 7 days, while methionine aminopeptidase 1 (MAP11), MYOF, and TFPI2 were downregulated. At 14 days of culture, 5′-3′ exonuclease PLD3 (PLD3) was upregulated, while tryptophan-tRNA ligase (SYWC) and plasminogen activator inhibitor 1 (PAI1) were downregulated.

**Table 6:** Proteins Differentially Expressed by HUCPV and CD14^+^ Co-Cultures Cultured on MT (Compared with Ti) at 7 and 14 Days with Functions on Cell Adhesion, Oxidative Stress, Osteogenesis, and Angiogenesis^a^

function		7 days	14 days
cell adhesion	↑ up	TARA	COTL1
	↓ down	MRP, LAMA, FBN1, LEG7, CYRIB, ARHG2, FGF2, MMP9, TBA1B, PDLI3, RAB13, ACTB, RALA, EPN4, ANXA2, CADH2, ANXA5, TBB5, TBB4B, MPRIP, PDLI2, ACTBL, ITA11, ITAV, ACTN1, ITA3, ARP3, PTMA, SNX2, ITA2, MMP14, MYO1C, ANXA1, ACTC, MA7D1, RHOC, TPM1, LEG3, MYL6, ARPC2, ANXA6, ITB1, FLNA, PTMS, MYH10, MYH9, CAPZB, CTNB1, TBB6, ARC1A, ML12A, RHG01, CKAP4, CTNA1, ACTN4, RAB1A, ARP2, ARPC5, ITA5, TPM3, CAD13, FINC, PDLI4, MAP4, ANXA4, TBB3, FLNC, PLEC, MYL9, ARPC4, GDIR1, CAZA1, ANX11, VINC, ARP10, ROCK2, SNX1, COF1, PAK2, PROF1, ITB5, TMOD3, RAI14, URP2, VIME, RAB14, GELS, K1C18, CAN2, TAGL2, VASP, TAGL, SPTB2, RAB13, ICAM2, FSCN1, PLSL, TWF2, COF2, ERLN2, CNN1, EMD, DCTN1, TPM4, DREB, ZYX, PALLD, TWF1, RAB6A, PROF2, RB11B, 1433Z, 1433B, EIF3C, 1433F, 1433E, 1433 T, 1433G, MOES	SPTN1, HNRPU, PLEC, CAN2, YKT6, SEP11, GELS, GDIR1, ARPC2, CALD1, S10A6, RAP1B, CNN3, TPM4, IF2B2, PDLI7, CSPG4, MYH10, IQGA1, ZO1, PICAL, MACF1, CAD13, ARHG2, RALA, TPM1, TARA, GDIA, RAB23, PDLI1, FBLN2, CAV1, NEXN, LTOR1, CTND1, SUN2
oxidative stress	↑ up	—	PRDX2, GPX1
	↓ down	NCF1C, PRDX2, GSTM2, GSTP1, PRDX1, CATA, TXND5, GSTK1, THIO, SODM, PRDX5, PRDX3, GSTO1, PXL2A, GSTM3, GPX8, TRXR1, PRDX6	NCF4, THTM
inflammation	↑ up	—	FABP5, COX41
	↓ down	HPT, CLUS, IGKC, ILF2, IGHG1, CD44, C5AR1, CAPG, IKIP, HLAB, MIF, ILF3, HLAA, CATB, CATZ, CATS, APOB, FCGRN, ENOA, CD166, PHB2, HM13, CHIT1, HMOX2, STML2, ILEU, APOL2, PYRG1, TFR1, LKHA4, CD59, LEG1	CATZ, ERAP1, TXLNA, ILF2, APOE, PTGR1, NCF2, PA1B2
osteogenesis	↑ up		PLOD2, EIF3K, IDE
	↓ down	EIF2A, EIF3M, PPA5, IF4H, IF4A2, PLOD2, CO6A1, CO6A2, IF2B1, KPCA, CO5A1, CO3A1, EIF3F, CTHR1, CO1A2, CO1A1, MK01, TIMP3, SERPH, SPRC, EIF3E, PLOD3, NID1, PLST, LIS1	CO1A1, IF2B1, RCN3, P3H1, CRTAP, SULF1, CO6A3, CO6A1, RFIP5, HDGF, CCN1
angiogenesis	↑ up	—	PLD3
	↓ down	MAP11, MYOF, TFPI2	SYWC, PAI1

a The proteins shown present *P* ≤ 0.05 and a ratio >1.5 in either direction (UP, increased and DOWN, reduced).

**Figure 4:**
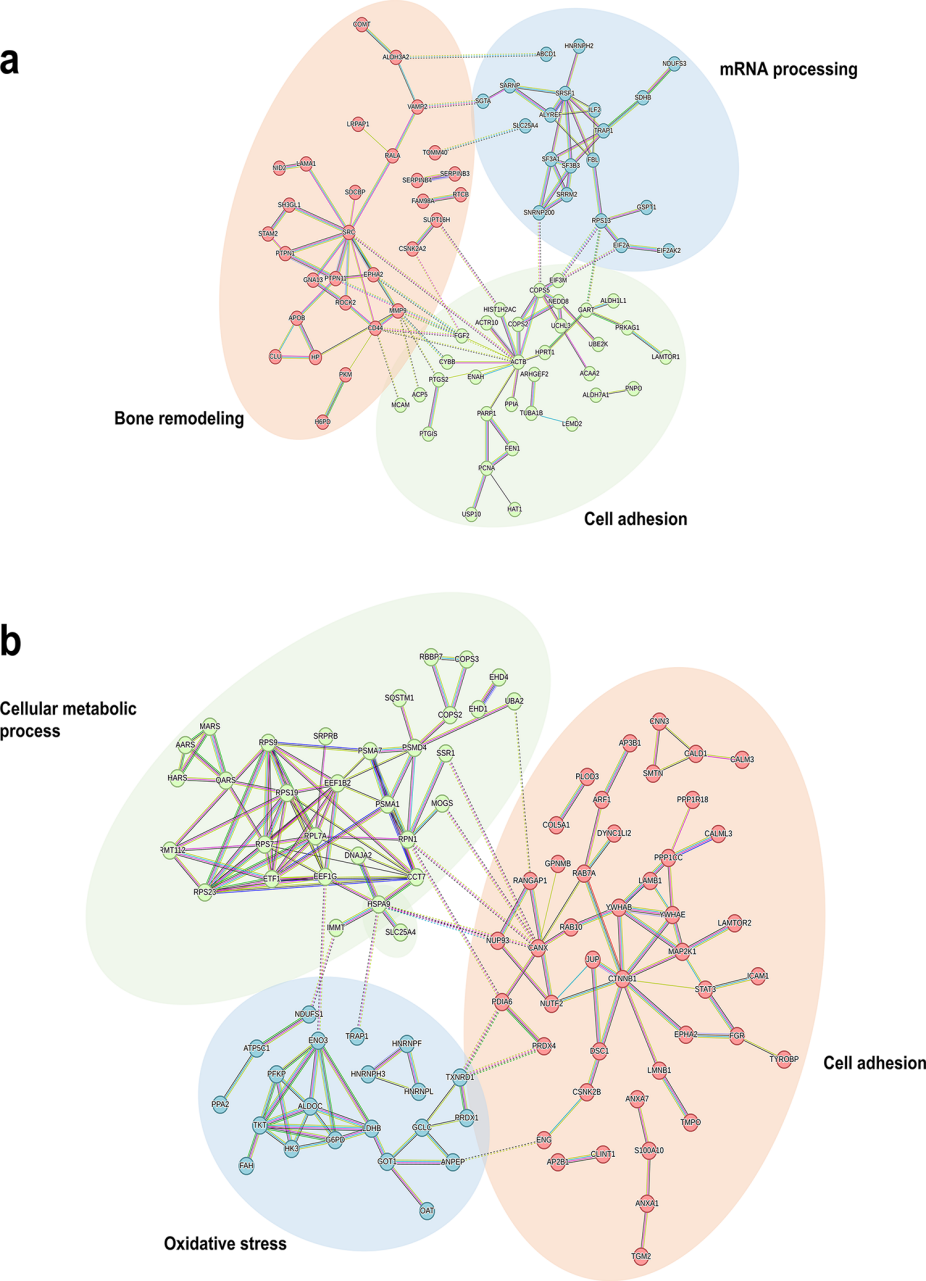
Protein interaction and cluster overview. STRING (v11.5) with K-means clustering to the first 150 differentially expressed proteins was employed. On 7 days (a), blue is for mRNA processing, green is for cell adhesion, and red is for the biosynthetic process. On 14 days (b), red is for cell adhesion, blue is for oxidative stress, and green is for the cellular metabolic process. Lines indicate interactions between proteins, and dashed lines indicate interactions inter-cluster.

To determine which protein clusters were significantly affected by MT in co-culture systems, STRING analysis was performed on the first 150 proteins expressed in the co-culture systems at 7 (Table S2) and 14 days (Table S3). After 7 days, three clusters associated with mRNA processing (40 proteins), cell adhesion (45 proteins), and bone remodeling (64 proteins) were identified (Figure 4a). At 14 days, three clusters associated with cell adhesion (69 proteins), oxidative stress (33 proteins), and cellular metabolic process (48 proteins) were identified (Figure 4b).

## 4 Discussion

The interaction between regenerative cells related to new bone formation and the immune system cells is a key factor for successful tissue regeneration and determines the fate of an implanted biomaterial. However, the characterization of the complex biomaterial-biological system interactions requires multidimensional investigation to elucidate the cell responses, which is not a trivial task. The use of proteomics allows obtaining the protein expression profiles of the cells exposed to the material and potentially overcomes the current challenges associated with traditional methods of in vitro characterization, where only a lower range of cell responses can be observed and measured with a limited number of experiments.^8^ It has been shown that results from different in vitro studies investigating the response of cells to biomaterials can be verified and explained by comprehensive proteomic studies.^10−12^ Here, we present the first characterization of the proteomic profile of co-culture systems composed of MSC (HUCPV) and immune cells (CD14^+^) on a sol–gel material.

The response of macrophages can present an immunomodulatory effect on MSC and further enhance the osteogenic differentiation of these cells that initially migrate to the bone injury site and differentiate into osteoblast-like cells.^3^ LC/MS–MS analysis of the HUCPV cells showed significant differences in the expression of only 69 and 87 proteins after 7 and 14 days of culture, respectively, in response to an MT-coated Ti-material compared to untreated Ti. PANTHER determined that integrin signaling cytoskeleton regulation by Rho GTPase and cadherin signaling were downregulated. These pathways are associated with cell adhesion, a process that triggers signaling responsible for cell differentiation, migration, and survival.^20,21^ Some of the proteins affected were integrin-linked protein kinase (ILK), integrin-α2 (ITA2), integrin-α11 (ITA11), integrin-β5 (ITB5), Ras homolog gene family, member C (RHOC), and cadherin-13 (CAD13). Integrins and cadherins are widely expressed receptors that anchor cells to the extracellular matrix (ECM) and activate countless signaling pathways that control cell cytoskeleton and signal transduction, regulating proliferation, differentiation, and migration.^22,23^ The microscopy results presented a decreased surface area covered by HUCPV cells seeded on MT, which correlates with the downregulation of proteins associated with this process. This is in accordance with previous studies^12,24^ that showed that MT does not improve cell adhesion and integrin expression in MC3T3-E1 osteoblastic cells when compared to Ti. In similarity to HUCPV cells, in co-cultures systems, a general downregulation of cell adhesion proteins such as integrins (ITA11, ITAV, ITA3, ITA2, ITB1, ITA5, ITB5), cadherins (CADH2, CAD13), actins (ACTB, ACTBL, ACTC, ACTN), myosins (MYH10, MYH9, MYO1C, MYL9, MYH10), RHOC, RHG01, and ROCK2, as well as two protein clusters, were found (via STRING). Also, an apparent decrease in the cell area in the co-cultures was observed when compared to Ti. In this type of system, the direct influence of macrophages in osteoblastic cell adhesion has been reported.^25^ In the present work, the greatest influence seems to come from the material rather than the interaction between cell types since these results can be correlated with the ones obtained in HUCPV monoculture.

When considering osteogenic protein expression, only nine proteins and one pathway (Wnt signaling) were found between MT and Ti on HUCPV cells, and no differences were detected in ALP activity at both time points. Martínez-Ibáñez et al.^16^ characterized MT in vitro with human adipose tissue-derived mesenchymal stem cells (AMSCs) showing that the material increased calcium-rich deposit formation. In vivo, it has been shown MT increases the formation of new bone trabeculae with higher relative length and density than Ti.^26,27^ However, as described by Araújo-Gomes et al.,^17,28^ MT does not induce changes ALP activity when compared to Ti, even though it increases the expression of IL-6 and osteocalcin (OCN) expression. In co-cultures, mainly collagens (CO5A1, CO3A1, CO6A1, CO6A2, CO1A2, CO1A1, CO6A3) were downregulated, while ALP was lower at 14 days. Collagen is the most abundant protein of the ECM and contributes to the regulation of hydroxyapatite deposition in bone, acting as a support for cells to attach and grow on through integrin-binding.^29^ This initiates and contributes to the commitment of MSCs toward the osteogenic lineage by leading to the activation of genes responsible for osteoblast differentiation and mineralization.^22^ Interestingly, the MT hybrid silica sol–gel nature gives this coating the ability to release silicon (Si), which has been described as a collagen I production promoter (as reviewed by O’Neill et al.^30^). However, it seems that the coating degradation enables optimal cell adhesion as shown by integrin expression downregulation, which may explain the decrease in collagen. With this, we can conclude that the osteogenic responses were affected by the reduction of cell adhesion (at least in the considered times).

On CD14^+^ cells, PANTHER analysis showed that MT led to an upregulation of integrin signaling, cytoskeleton regulation by Rho GTPase, Ras pathway, and cadherin signaling. Several integrins (ITA5, ITB2, ITAX, ITB5, ITB1, ITA11, ITAM) and proteins belonging to the Rho family GTPases (Rho, Rac, and Cdc42) were upregulated by MT on CD14^+^ cells. Cell division control protein 42 homolog (CDC42) and Ras-related C3 botulinum toxin substrate 2 (Rac2) are known for regulating the formation of lamellipodial and filopodial membrane protrusions through Arp2/3 complex-mediated actin polymerization.^21,31^ These proteins in association with Rho (RhoA, RhoB, and RhoC) are crucial for actomyosin remodeling and cytoskeletal dynamics.^23^ Actins (ACTBL, ACTN4, ACTN1, ACTC, ACTB, ACTN1) and myosins (MYL6, MYO1B, MYH9, MYOF1), critical players in cell motility, cell shape maintenance, cytoskeletal regulation and contraction, and cellular force maintenance,^32^ were also upregulated. Moreover, it has been suggested that Rho GTPases regulate cell cytoskeleton remodeling and migration in conjunction with 14-3-3 proteins, which indicates a possible regulation between the two groups of proteins.^33^ Thus, it was unsurprising to find that several proteins belonging to the 14-3-3 family (1433Z, 1433T, 1433G, 1433E, 1433F, 1433B) were upregulated at both time points on CD14^+^ cells. These proteins bind to the phosphorylated Ser/Thr residues of a wide range of proteins involved in cell signaling, transcription regulation, cytoskeleton remolding, DNA repair, and apoptosis.^33^ Interestingly, the effect of MT on the macrophage cell area can be associated with anti-inflammatory responses. Zhu et al.^34^ showed that a higher expression of M2 (anti-inflammatory) markers (CD206, IL-4, IL-10) correlated with a higher cell area facilitated macrophage filopodia formation and up-regulation of the Rho family (RhoA, Rac1, and CDC42) expression.

Moreover, only CD14^+^ and co-cultures showed significant differences in proteins associated with oxidative stress. Upon biomaterial implantation, oxidative stress is generated and reactive oxygen species (ROS) act as signals in many intracellular signaling pathways. On macrophages, peroxiredoxins (PRDX3, PRDX4, PRDX1, PRDX6), glutathione transferase (GSTO1), glutathione peroxidase (GPX1), glutathione reductase (GSHR), catalase (CATA), and superoxide dismutase (SODM) expression were increased. On co-cultures, some of these proteins (PRDX2, PRDX6, GSTM2, GSTP1, PRDX1, CATA, SODM, PRDX5, PRDX3, GSTO1, GSTM3, GPX8) were downregulated. These molecules are “scavengers” capable of shutting down oxidative chain reactions. ROS are critical for M1/M2 activation and polarization, and the modulation of the oxidative stress response by biomaterials can affect macrophage responses and new bone tissue deposition.^35^ The increase of ROS scavenging activity by bioactive coatings has been shown to decrease immune cell infiltration^36^ and is associated with the M2 phenotype in natural melanin/alginate hydrogels.^37^ The expression of proteins associated with inflammatory responses showed similar patterns in both conditions (CD14^+^ and co-cultures). Pathways such as interleukin signaling, inflammation by chemokine and cytokine, TGF-β signaling, T cell, and B cell activation were significantly affected. On CD14^+^ cells, apolipoproteins (APOE and APOL2) and galectins (LEG9, LEG3, LEG1) were upregulated. Apolipoproteins are known for exerting effects on inflammation; for example, APOE has immune modulatory properties, inducing macrophage polarization into the M2 phenotype.^38^ In line with this, galectin-3 (LEG3) promotes the alternative activation of macrophages, while galectin-9 (LEG9) induces Th2/M2 differentiation in immune cells, and galectin-1 facilitates inflammation resolution.^39^ In contrast, in the co-culture systems, there was a significant decrease in inflammatory protein expression. Few anti-inflammatory proteins were affected (LEG1, APOE, HPT, CLUS) and a greater number of proteins associated with pro-inflammatory responses were downregulated (C5AR1, ENOA, PHB2, IKIP, LKHA4). C5a anaphylatoxin chemotactic receptor 1 (C5AR1) is associated with the complement cascade being one of the two receptors to which complement factor C5a binds and has been associated with inflammatory disorders such as sepsis, rheumatoid arthritis, and psoriasis.^40^ The enzyme α-enolase (ENOA) stimulates the pro-inflammatory cytokine production (TNF-α, IL-1α/β, IFN-γ) and facilitates fibrinolysis by acting as a plasminogen receptor and degrading ECM.^41^ Other downregulated proteins were inhibitors of nuclear factor kappa-B kinase-interacting protein (IKIP), which is a key regulator of the nuclear factor kappa B (NF-kB) cascade,^42^ and leukotriene A_4_ hydrolase (LKHA4), an inductor of neutrophilic infiltration in many inflammatory diseases.^43^ In addition, three cathepsins (CATB, CATZ, CATS), which have significant roles in the immune responses and are related to the NF-kB pathway,^44^ were also significantly affected. Silica-based materials are known for inhibiting M1 polarization (pro-inflammatory) by reducing the expression of specific cell surface markers and pro-inflammatory cytokine production.^45−47^ MT has an anti-inflammatory potential decreasing IL7-R expression and tumor necrosis factor (TNF)-α release (M1 markers) in RAW264.7 cells while presenting lower affinity with complement system proteins.^27,48^ This correlates with in vivo *results* since it has been shown that MT reduces the relative density and the size of osteoclast-like and giant multinucleated cells was lower when compared to Ti.^26,27^ The overall results englobing cell adhesion, oxidative stress, and inflammation show the anti-inflammatory potential of MT and are in accordance with the previous results, giving an insight into the polarization mechanisms in response to the material. With this, we can infer that Si-based coatings alone may induce an anti-inflammatory response in CD14^+^ cells. On co-cultures, the effect may be more accentuated since MSCs can induce IL-10 production in macrophages in co-culture systems,^47^ which was verified in the significant increase of this cytokine concentration at 14 days.

The results obtained demonstrate the potential of proteomics to better understand the complex cell-material interactions as well as the interactions between cell lines, which may regulate the responses to the said material. With the present co-culture study, we showed that protein networks involved in cell adhesion seem to be mainly influenced by the material surface whereas inflammatory cascades were influenced by the cellular crosstalk and the material. However, more studies on far more complex systems are needed to ensure that the results observed in vitro can be translated into what will be obtained in vivo.

## 5 Conclusions

This work aimed to analyze through mass spectrometry the protein expression patterns of co-cultures exposed to a sol–gel coating for 7 and 14 days in vitro. The results show that MT decreased the expression of proteins associated with cell adhesion in HUCPV and co-cultures. No differences were found in osteogenesis in HUCPV; however, a decrease in collagen expression was detected in co-cultures. In CD14^+^ cells, an increment in proteins related to cell adhesion, the antioxidant system, and anti-inflammatory responses was detected in MT. On co-cultures, pro-inflammatory proteins were less expressed and an increase in IL-10 production was detected in MT. With these results, we showed that cell adhesion seems to be mainly influenced by the material. On the other hand, inflammation appears to be impacted by cellular crosstalk and the material since MT regulates inflammatory responses by inducing macrophage polarization toward an anti-inflammatory phenotype. Proteomics allowed us to identify the proteins and mechanisms associated with this anti-inflammatory behavior of MT. These findings indicate that proteomics has the potential to enhance our understanding of the intricate interactions between cells and materials, even in complex systems such as co-cultures.

## Supporting Information Available

List of proteins differentially expressed in HUCPV, CD14^+^, and co-culture systems exposed to MT in relation to Ti (PDF)
First 150 proteins differentially expressed in co-culture systems exposed to MT in relation to Ti after 7 days of assay (PDF)
First 150 proteins differentially expressed in co-culture systems exposed to MT in relation to Ti after 14 days of assay (PDF)
