# Supplementary material for: Proteomic Analysis of Mesenchymal Stem Cells and Monocyte Co-Cultures Exposed to a Bioactive Silica-Based Sol–Gel Coating
Source: ACS Biomater Sci Eng. 2023 May 19;9(6):3306–19. doi: 10.1021/acsbiomaterials.3c00254 (PMC10265575; doi:10.1021/acsbiomaterials.3c00254)
Supplement: Supplementary file 1 — ab3c00254_si_001.pdf [file ab3c00254_si_001.pdf]

Proteomic analysis of mesenchymal stem cells and monocytes co-cultures exposed to a bioactive silica-based sol-gel coating

Andreia Cerqueira[1*], Francisco Romero-Gavilán[1], Heike Helmholz[2], Mikel Azkargorta[3], Félix Elortza[3], Mariló Gurruchaga[4], Isabel Goñi[4], Regine Willumeit-Römer[2], Julio Suay[1]

[1]Department of Industrial Systems Engineering and Design, Universitat Jaume I, Av. Vicent Sos Baynat s/n, 12071 Castellón de la Plana, Spain

[2]Helmholtz-Zentrum Hereon Institute of Metallic Biomaterials, Max-Planck-St.1, Geesthacht D-21502, Germany

[3]Proteomics Platform, CIC bioGUNE, Basque Research and Technology Alliance (BRTA), CIBERehd, Bizkaia Science and Technology Park, 48160 Derio, Spain

[4]Departament of Science and Technology of Polymers, University of the Basque Country, P. M. de Lardizábal, 3, 20018 San Sebastián, Spain

*Corresponding author: Andreia Cerqueira. E-mail: lages@uji.es

Supplementary Information: 61 pages

Table S1. List of proteins differentially expressed in HUCPV, $CD14^+$, and co-culture systems exposed to MT in relation to Ti.

Table S1. Comparative analysis between the proteins differentially expressed in HUCPV, $CD14^{+}$, and co-culture systems exposed to MT in relation to Ti. Proteins with ANOVA $p < 0.05$ (yellow) and a ratio higher than 1.5 in either direction were considered as significantly different. Upregulated proteins are marked in red and downregulated proteins appear in green.

				**HUCPV**				**CD14+**				**Co-culture**			
				**7 days**		**14 days**		**7 days**		**14 days**		**7 days**		**14 days**	
Accession	Description	Peptides	Unique peptides	p value	Ratio	p value	Ratio	p value	Ratio	p value	Ratio	p value	Ratio	p value	Ratio
Q01469	FABP5_HUMAN Fatty acid-binding protein 5	5	5	7,24E-02	0,14	1,00E+00	1,00	5,36E-01	2,31	2,16E-01	3,67	3,67E-02	0,99	5,24E-03	12,89
P31151	S10A7_HUMAN Protein S100-A7	6	6	4,91E-01	2,24	6,18E-01	0,56	4,44E-02	31,76	6,01E-01	0,52	4,91E-01	0,77	2,63E-01	8,98
P43007	SATT_HUMAN Neutral amino acid transporter A	4	4	1,00E+00	1,00	4,55E-01	1,41	1,00E+00	1,00	8,28E-02	6,02	5,49E-02	0,99	3,62E-03	6,52
P46776	RL27A_HUMAN 60S ribosomal protein L27a	3	3	2,17E-01	0,16	7,29E-01	0,88	3,46E-01	1,39	5,87E-06	26,29	9,18E-02	0,98	8,75E-02	5,73
Q8NF91	SYNE1_HUMAN Nesprin-1	3	3	9,95E-01	1,02	1,88E-02	0,01	6,05E-01	0,65	9,27E-01	0,79	7,19E-01	0,63	3,92E-01	5,59
Q96IX5	ATPMD_HUMAN ATP synthase membrane subunit DAPIT, mitochondrial	3	3	7,54E-01	0,54	6,67E-01	1,14	8,60E-01	0,69	7,60E-01	1,11	2,04E-02	0,99	3,39E-02	5,37
O60493	SNX3_HUMAN Sorting nexin-3	2	2	2,71E-01	0,36	8,76E-03	0,08	1,00E+00	1,00	6,61E-01	0,69	1,80E-01	0,96	1,54E-01	4,72
Q9NZT1	CALL5_HUMAN Calmodulin-like protein 5	6	6	5,48E-01	0,50	5,45E-01	0,28	2,86E-01	2,97	1,87E-01	0,06	1,15E-01	0,98	5,04E-03	4,60
P49748	ACADV_HUMAN Very long-chain specific acyl-CoA dehydrogenase, mitochondrial	14	13	1,36E-01	0,16	9,15E-01	1,10	2,24E-01	1,77	4,26E-03	48,28	2,44E-02	0,99	2,46E-01	4,55
P33121	ACSL1_HUMAN Long-chain-fatty-acid--CoA ligase 1	12	12	5,87E-01	0,79	4,86E-01	1,29	1,00E+00	1,00	3,90E-06	193,40	1,08E-01	0,98	9,71E-02	4,45
Q9Y383	LC7L2_HUMAN Putative	2	2	6,58E-	1,57	1,84E-	1,63	1,00E+	1,00	1,00E+	1,00	8,19E-	1,00	2,14E-	4,42

	RNA-binding protein Luc7-like 2			01		03		00		00		03		01	
P20702	ITAX_HUMAN Integrin alpha-X	20	19	3,94E-01	1,60	4,78E-01	1,35	5,81E-01	1,47	5,41E-03	130,79	2,43E-01	0,93	7,34E-02	4,40
P53992	SC24C_HUMAN Protein transport protein Sec24C	4	4	1,62E-02	22,69	1,97E-02	1,94	8,54E-02	11,46	1,79E-01	2,66	9,64E-01	0,52	2,47E-01	3,57
P05091	ALDH2_HUMAN Aldehyde dehydrogenase, mitochondrial	11	11	8,95E-01	0,88	6,45E-02	0,38	7,08E-02	0,20	2,74E-03	42,67	1,78E-01	0,96	3,62E-03	3,40
Q9UDY4	DNJB4_HUMAN DnaJ homolog subfamily B member 4	6	5	2,79E-02	0,11	3,96E-01	0,46	6,20E-01	0,58	1,48E-01	2,32	1,43E-01	0,97	2,24E-01	3,23
Q9Y6N5	SQOR_HUMAN Sulfide:quinone oxidoreductase, mitochondrial	19	19	3,62E-01	0,60	9,35E-02	0,29	7,12E-02	41,04	1,01E-03	541,11	9,67E-01	0,52	3,29E-01	2,99
O15173	PGRC2_HUMAN Membrane-associated progesterone receptor component 2	6	6	2,87E-01	0,29	8,84E-02	0,30	6,77E-01	1,15	2,85E-02	3,79	4,39E-01	0,80	1,54E-01	2,87
O00764	PDXK_HUMAN Pyridoxal kinase	5	5	6,09E-01	2,16	3,24E-01	0,45	4,95E-01	1,64	1,47E-02	23,61	1,63E-01	0,96	1,77E-01	2,82
P40616	ARL1_HUMAN ADP-ribosylation factor-like protein 1	2	2	1,52E-01	0,17	7,01E-03	0,20	1,00E+00	1,00	1,00E+00	1,00	1,36E-01	0,97	1,97E-01	2,81
Q7Z3B1	NEGR1_HUMAN Neuronal growth regulator 1	2	2	6,47E-01	0,85	8,25E-01	1,25	1,00E+00	1,00	1,00E+00	1,00	1,00E+00	0,50	4,68E-01	2,79
P24752	THIL_HUMAN Acetyl-CoA acetyltransferase, mitochondrial	6	6	2,37E-02	0,03	2,83E-01	1,43	2,89E-01	4,41	4,13E-07	230,38	6,07E-01	0,70	9,27E-02	2,72
P19784	CSK22_HUMAN Casein kinase II subunit alpha	2	2	5,24E-01	2,82	2,57E-01	0,24	7,57E-02	34,85	1,00E+00	1,00	3,63E-03	1,00	1,07E-02	2,70
P84077	ARF1_HUMAN ADP-ribosylation factor 1	9	4	5,89E-01	0,58	1,26E-01	1,33	2,22E-01	5,44	6,03E-04	110,06	2,34E-01	0,93	1,19E-01	2,43
Q14019	COTL1_HUMAN Coactosin-like protein	7	7	5,77E-02	0,08	4,13E-02	0,55	1,49E-01	4,91	6,83E-03	27,09	1,84E-01	0,96	8,53E-03	2,39

P10599	THIO_HUMAN Thioredoxin	5	5	1,94E-01	0,15	4,20E-01	0,74	7,11E-01	0,52	2,60E-01	4,00	9,68E-03	1,00	4,41E-01	2,38
Q0VD83	APOBR_HUMAN Apolipoprotein B receptor	8	8	4,81E-02	19,49	2,63E-01	1,53	8,50E-01	1,45	1,95E-01	7,05	8,96E-01	0,55	2,16E-01	2,23
Q14677	EPN4_HUMAN Clathrin interactor 1	5	5	9,48E-01	0,94	2,74E-01	0,43	6,85E-01	1,32	4,95E-02	1,60	2,76E-03	1,00	4,19E-01	2,21
O00469	PLOD2_HUMAN Procollagen-lysine,2-oxoglutarate 5-dioxygenase 2	25	25	4,50E-01	0,31	7,67E-02	1,49	1,00E+00	1,00	7,66E-02	3,43	3,62E-03	1,00	4,58E-03	2,21
P48506	GSH1_HUMAN Glutamate--cysteine ligase catalytic subunit	9	9	6,61E-01	0,80	7,45E-01	1,30	2,34E-01	1,60	1,31E-02	17,84	2,77E-01	0,91	1,04E-01	2,18
Q9UBQ5	EIF3K_HUMAN Eukaryotic translation initiation factor 3 subunit K	2	2	5,11E-01	0,28	7,59E-01	0,93	1,89E-01	2,39	1,25E-01	3,08	2,19E-01	0,94	5,02E-03	2,14
Q96AQ6	PBIP1_HUMAN Pre-B-cell leukemia transcription factor-interacting protein 1	5	5	1,82E-02	0,12	3,51E-01	0,45	9,71E-01	0,97	1,00E+00	1,00	3,01E-02	0,99	1,48E-01	2,14
Q9Y5S1	TRPV2_HUMAN Transient receptor potential cation channel subfamily V member 2	5	5	9,96E-02	0,31	3,21E-01	0,51	1,00E+00	1,00	6,09E-05	53,66	4,82E-02	0,99	8,95E-02	2,11
P53007	TXTP_HUMAN Tricarboxylate transport protein, mitochondrial	3	3	1,92E-01	0,52	5,05E-01	0,61	8,71E-01	0,87	2,05E-01	3,74	3,51E-02	0,99	2,38E-01	2,08
Q8IV08	PLD3_HUMAN 5-3 exonuclease PLD3	4	4	8,56E-01	1,36	6,04E-01	0,63	5,90E-01	2,25	2,93E-03	61,61	1,05E-01	0,98	4,74E-02	2,05
P62906	RL10A_HUMAN 60S ribosomal protein L10a	10	10	1,84E-02	0,05	3,68E-01	1,49	6,55E-01	2,06	2,99E-01	4,67	2,21E-01	0,94	7,02E-03	2,04
P49773	HINT1_HUMAN Histidine triad nucleotide-binding protein 1	3	3	1,00E+00	1,00	2,22E-02	0,31	1,00E+00	1,00	1,00E+00	1,00	2,32E-01	0,93	2,59E-01	2,00

Q9NP72	RAB18_HUMAN Ras-related protein Rab-18	6	6	7,64E-01	0,67	5,14E-01	0,82	8,99E-01	0,90	4,54E-06	79,35	5,11E-02	0,99	2,29E-01	1,99
P21399	ACOC_HUMAN Cytoplasmic aconitate hydratase	7	7	5,85E-01	0,68	1,65E-01	0,21	6,49E-01	1,25	4,07E-02	4,04	3,37E-01	0,87	2,63E-01	1,97
P68871	HBB_HUMAN Hemoglobin subunit beta	7	3	1,66E-01	7,38	4,39E-02	4,58	2,95E-01	1,74	6,31E-02	0,54	3,07E-01	0,89	5,04E-01	1,95
P09668	CATH_HUMAN Pro-cathepsin H	2	2	1,00E+00	1,00	2,62E-02	0,12	1,00E+00	1,00	2,37E-05	47,56	1,12E-01	0,98	5,91E-01	1,94
P43353	AL3B1_HUMAN Aldehyde dehydrogenase family 3 member B1	2	2	9,67E-01	1,05	4,42E-01	0,80	1,00E+00	1,00	1,33E-02	39,51	1,00E+00	0,50	2,99E-01	1,86
Q9UI12	VATH_HUMAN V-type proton ATPase subunit H	4	4	6,94E-01	1,37	3,14E-02	0,22	1,00E+00	1,00	2,56E-05	55,99	7,06E-02	0,99	4,82E-01	1,86
Q6PIU2	NCEH1_HUMAN Neutral cholesterol ester hydrolase 1	11	10	3,04E-01	0,32	5,42E-01	0,72	1,77E-01	4,28	6,25E-04	164,00	2,34E-01	0,93	4,60E-01	1,80
Q16719	KYNU_HUMAN Kynureninase	3	3	1,18E-01	0,61	4,83E-02	0,35	2,91E-01	2,11	2,05E-02	49,80	7,45E-02	0,98	2,63E-01	1,79
O60784	TOM1_HUMAN Target of Myb protein 1	5	4	8,07E-01	1,22	8,25E-01	0,91	5,72E-01	2,60	6,76E-01	2,28	2,06E-02	0,99	5,63E-01	1,78
Q14956	GPNMB_HUMAN Transmembrane glycoprotein NMB	2	2	1,00E+00	1,00	5,34E-01	0,71	3,83E-01	4,22	1,04E-02	100,11	7,77E-03	1,00	7,10E-01	1,75
P30086	PEBP1_HUMAN Phosphatidylethanolamine-binding protein 1	6	6	2,65E-01	0,12	2,07E-01	0,73	3,54E-01	0,28	1,10E-03	36,40	2,08E-02	0,99	2,70E-01	1,74
P20645	MPRD_HUMAN Cation-dependent mannose-6-phosphate receptor	3	3	2,05E-01	0,34	3,30E-01	0,39	1,00E+00	1,00	1,97E-04	58,67	4,58E-02	0,99	2,71E-01	1,74
P11498	PYC_HUMAN Pyruvate carboxylase, mitochondrial	7	7	5,71E-01	0,40	6,16E-01	1,33	6,18E-01	1,74	4,68E-02	28,66	2,14E-04	1,00	3,58E-01	1,73
P30519	HMOX2_HUMAN Heme oxygenase 2	5	5	7,45E-02	0,12	8,17E-01	1,05	1,00E+00	1,00	3,14E-02	3,65	2,33E-02	0,99	3,57E-01	1,72

P26447	S10A4_HUMAN Protein S100-A4	2	2	2,90E-01	1,55	1,49E-01	0,53	9,54E-01	0,93	2,71E-04	113,37	1,39E-02	1,00	3,18E-01	1,72
Q96C23	GALM_HUMAN Aldose 1-epimerase	4	4	6,99E-01	1,34	7,07E-01	1,40	2,18E-01	1,54	3,48E-04	99,05	8,37E-01	0,58	4,66E-01	1,72
P08574	CY1_HUMAN Cytochrome c1, heme protein, mitochondrial	2	2	5,39E-01	0,74	8,99E-01	0,93	1,00E+00	1,00	7,36E-03	5,80	2,83E-02	0,99	2,73E-01	1,69
P24534	EF1B_HUMAN Elongation factor 1-beta	6	5	3,13E-01	0,11	3,26E-01	0,76	1,00E+00	1,00	2,83E-02	12,17	5,98E-06	1,00	3,65E-01	1,67
P14136	GFAP_HUMAN Glial fibrillary acidic protein	4	3	2,87E-01	0,30	2,58E-02	6,63	7,85E-01	0,80	1,11E-01	2,76	1,94E-03	1,00	4,56E-01	1,66
Q8TD55	PKHO2_HUMAN Pleckstrin homology domain-containing family member 2	3	3	9,34E-02	0,42	1,31E-02	0,13	1,00E+00	1,00	3,39E-05	71,37	5,08E-02	0,99	2,83E-01	1,65
P32119	PRDX2_HUMAN Peroxiredoxin-2	8	7	5,03E-01	0,36	6,90E-01	1,17	1,00E+00	1,00	1,08E-01	6,23	2,22E-02	0,99	2,79E-02	1,64
Q93050	VPP1_HUMAN V-type proton ATPase 116 kDa subunit a isoform 1	2	2	3,41E-01	0,36	8,25E-01	1,16	1,00E+00	1,00	1,94E-02	5,32	3,20E-05	1,00	4,75E-01	1,63
Q15388	TOM20_HUMAN Mitochondrial import receptor subunit T M20 homolog	2	2	8,20E-01	0,68	4,50E-01	1,25	1,00E+00	1,00	1,06E-01	4,97	1,18E-02	1,00	3,48E-03	1,63
Q96P70	IPO9_HUMAN Importin-9	2	2	4,27E-02	0,10	4,87E-01	1,17	1,00E+00	1,00	1,66E-01	2,05	2,01E-01	0,95	5,89E-01	1,63
O75521	ECI2_HUMAN Enoyl-CoA delta isomerase 2, mitochondrial	5	5	3,25E-01	4,96	2,79E-01	0,36	5,30E-01	0,27	2,50E-01	5,67	9,51E-01	0,52	1,64E-03	1,59
P25398	RS12_HUMAN 40S ribosomal protein S12	6	6	8,08E-01	0,68	9,46E-01	1,02	1,00E+00	1,00	3,79E-06	33,47	3,22E-04	1,00	2,43E-01	1,58
P14866	HNRPL_HUMAN Heterogeneous nuclear ribonucleoprotein L	7	7	8,53E-01	0,81	8,73E-01	0,88	1,05E-01	0,11	3,71E-02	6,95	5,60E-01	0,72	7,37E-01	1,57

Q58FF8	H90B2_HUMAN Putative heat shock protein HSP 90-beta 2	12	2	1,17E-01	0,12	9,72E-01	1,07	1,00E+00	1,00	2,06E-02	1,77	1,52E-01	0,97	1,71E-01	1,54
Q9BTU6	P4K2A_HUMAN Phosphatidylinositol 4-kinase type 2-alpha	8	8	1,73E-01	0,07	6,23E-01	1,63	7,85E-01	1,85	4,67E-02	35,70	9,93E-01	0,50	4,04E-01	1,54
Q99426	TBCB_HUMAN Tubulin-folding cofactor B	3	3	8,49E-01	0,70	3,05E-01	0,58	1,00E+00	1,00	4,56E-02	13,54	1,65E-02	0,99	5,75E-02	1,53
P07900-2	HS90A_HUMAN2 Isoform 2 of Heat shock protein HSP 90-alpha	41	22	2,68E-01	0,42	5,75E-01	1,05	2,95E-01	5,26	2,67E-03	6,59	2,59E-03	1,00	5,63E-02	1,53
P42677	RS27_HUMAN 40S ribosomal protein S27	2	1	1,55E-01	0,14	3,91E-02	1,95	1,00E+00	1,00	2,74E-01	1,46	6,73E-02	0,99	6,45E-01	1,52
P15531	NDKA_HUMAN Nucleoside diphosphate kinase A	9	4	3,17E-02	0,02	1,31E-01	0,72	8,05E-01	0,87	9,97E-02	3,02	9,79E-01	0,51	6,67E-01	1,51
P63220	RS21_HUMAN 40S ribosomal protein S21	2	2	2,65E-01	0,60	5,29E-01	1,16	8,43E-01	1,17	1,00E+00	1,00	4,04E-02	0,99	7,07E-01	1,51
P62304	RUXE_HUMAN Small nuclear ribonucleoprotein E	3	3	3,66E-01	0,27	2,80E-01	1,70	1,00E+00	1,00	6,33E-02	2,14	1,71E-01	0,96	3,63E-01	1,51
P24539	AT5F1_HUMAN ATP synthase F(0) complex subunit B1, mitochondrial	6	5	2,25E-01	0,23	4,47E-01	0,85	1,00E+00	1,00	1,72E-04	30,97	2,77E-01	0,91	3,72E-01	1,49
P16070	CD44_HUMAN CD44 antigen	7	7	2,17E-01	0,09	3,21E-01	0,85	8,80E-01	0,80	6,16E-04	100,18	3,60E-03	1,00	3,16E-02	1,48
P29350	PTN6_HUMAN Tyrosine-protein phosphatase non-receptor type 6	10	10	9,13E-01	1,09	1,75E-01	0,28	9,87E-01	0,98	1,39E-03	87,38	3,46E-01	0,87	6,15E-01	1,48
P23528	COF1_HUMAN Cofilin-1	11	6	2,24E-01	0,39	8,79E-01	1,02	1,14E-01	11,18	4,46E-02	28,45	2,65E-03	1,00	9,28E-02	1,48
P62269	RS18_HUMAN 40S ribosomal protein S18	10	10	5,88E-01	0,39	5,57E-01	0,71	2,53E-01	6,45	2,58E-04	10,48	6,55E-02	0,99	2,09E-01	1,48
Q06830	PRDX1_HUMAN Peroxiredoxin-1	15	12	2,27E-01	0,24	1,13E-01	1,22	5,14E-01	2,66	2,43E-04	15,87	2,25E-02	0,99	5,85E-02	1,48

Q00688	FKBP3_HUMAN Peptidyl-prolyl cis-trans isomerase FKBP3	2	2	6,20E-01	0,71	4,61E-02	5,92	1,00E+00	1,00	1,00E+00	1,00	1,00E+00	0,50	1,94E-01	1,47
P09382	LEG1_HUMAN Galectin-1	10	9	1,57E-01	0,09	3,34E-01	1,43	9,78E-01	1,04	2,16E-03	46,77	1,40E-02	1,00	7,41E-02	1,46
P30048	PRDX3_HUMAN Thioredoxin-dependent peroxide reductase, mitochondrial	4	4	4,45E-01	0,31	2,46E-01	0,80	1,00E+00	1,00	1,16E-03	79,38	7,65E-03	1,00	3,92E-02	1,43
P13667	PDIA4_HUMAN Protein disulfide-isomerase A4	33	33	1,81E-01	0,08	1,18E-01	1,34	1,29E-01	3,51	2,94E-04	51,77	2,87E-03	1,00	1,37E-01	1,43
Q02878	RL6_HUMAN 60S ribosomal protein L6	14	14	1,92E-01	0,11	4,07E-01	1,11	1,30E-01	3,02	1,22E-03	40,66	9,69E-03	1,00	9,02E-02	1,42
P01889	HLAB_HUMAN HLA class I histocompatibility antigen, B alpha chain	7	3	8,81E-01	1,07	1,38E-01	0,24	1,00E+00	1,00	3,41E-05	190,65	4,51E-02	0,99	4,13E-01	1,41
P33241	LSP1_HUMAN Lymphocyte-specific protein 1	3	3	1,00E+00	1,00	8,08E-02	2,19	1,00E+00	1,00	4,32E-03	73,12	1,04E-01	0,98	4,44E-01	1,41
Q6IBS0	TWF2_HUMAN Twinfilin-2	8	8	7,73E-01	0,62	3,80E-01	0,55	1,68E-01	2,14	4,87E-06	66,44	2,84E-03	1,00	2,96E-01	1,40
P68363	TBA1B_HUMAN Tubulin alpha-1B chain	26	0	1,75E-01	0,39	5,56E-01	1,10	5,41E-02	8,30	8,12E-05	15,51	2,96E-03	1,00	1,86E-02	1,40
P05783	K1C18_HUMAN Keratin, type I cytoskeletal 18	39	26	1,78E-01	0,18	3,22E-02	1,73	4,10E-01	10,37	9,79E-01	1,03	4,53E-02	0,99	1,71E-01	1,40
Q15366	PCBP2_HUMAN Poly(rC)-binding protein 2	7	5	2,41E-01	0,11	8,02E-01	1,07	1,00E+00	1,00	9,96E-03	11,56	6,15E-04	1,00	1,58E-01	1,40
P39019	RS19_HUMAN 40S ribosomal protein S19	7	7	6,76E-01	1,84	1,61E-01	1,25	2,06E-01	9,39	1,67E-03	0,08	2,62E-01	0,92	8,17E-02	1,38
Q92626	PXDN_HUMAN Peroxidasin homolog	5	5	3,44E-01	0,27	8,71E-01	0,89	3,27E-02	3,54	8,69E-01	0,84	3,81E-02	0,99	3,68E-01	1,38
Q9BTV4	TMM43_HUMAN Transmembrane protein 43	10	10	4,87E-02	16,07	2,41E-01	0,74	9,59E-02	2,75	7,94E-02	7,12	1,46E-01	0,97	6,82E-01	1,38

P08238	HS90B_HUMAN Heat shock protein HSP 90-beta	41	19	2,39E-01	0,34	6,95E-02	1,14	7,11E-01	1,59	3,46E-03	152,16	1,18E-02	1,00	7,27E-02	1,38
P62826	RAN_HUMAN GTP-binding nuclear protein Ran	11	11	3,14E-01	0,18	9,12E-01	0,99	7,90E-01	0,65	9,49E-04	7,45	1,13E-02	1,00	8,19E-02	1,37
Q9UL46	PSME2_HUMAN Proteasome activator complex subunit 2	6	6	1,21E-02	0,09	2,18E-01	0,57	4,46E-01	0,42	9,78E-05	36,23	8,74E-01	0,56	6,22E-01	1,37
P30050	RL12_HUMAN 60S ribosomal protein L12	4	4	1,09E-01	0,36	2,52E-01	1,26	8,85E-01	1,28	2,34E-05	40,13	5,03E-03	1,00	1,47E-01	1,37
P14174	MIF_HUMAN Macrophage migration inhibitory factor	2	2	5,44E-01	0,50	4,66E-01	0,83	8,48E-01	0,83	1,77E-02	14,29	1,82E-03	1,00	3,68E-01	1,37
P11215	ITAM_HUMAN Integrin alpha-M	24	23	7,45E-02	4,86	4,32E-01	1,67	6,88E-01	0,77	7,16E-07	372,54	6,86E-01	0,65	9,92E-02	1,37
P04899	GNAI2_HUMAN Guanine nucleotide-binding protein G(i) subunit alpha-2	8	5	1,78E-01	0,31	4,67E-01	0,95	3,24E-01	4,18	3,96E-03	64,54	3,13E-01	0,89	3,33E-01	1,36
P61421	VA0D1_HUMAN V-type proton ATPase subunit d 1	4	4	1,52E-01	0,31	5,77E-01	0,61	5,36E-02	2,37	1,75E-05	192,51	8,56E-02	0,98	7,47E-01	1,36
P62937	PPIA_HUMAN Peptidyl-prolyl cis-trans isomerase A	12	12	2,36E-01	0,19	7,34E-01	1,07	1,73E-01	10,09	9,79E-05	163,82	1,07E-03	1,00	2,24E-01	1,36
P02786	TFR1_HUMAN Transferrin receptor protein 1	20	20	5,24E-01	0,46	9,66E-01	1,01	5,82E-01	2,12	8,32E-04	169,64	1,53E-03	1,00	4,76E-02	1,36
Q9HB71	CYBP_HUMAN Calcyclin-binding protein	5	5	8,33E-02	2,77	5,74E-01	1,31	5,43E-01	3,78	2,26E-01	22,26	3,15E-02	0,99	3,31E-01	1,36
Q9NX63	MIC19_HUMAN MICOS complex subunit MIC19	4	4	9,60E-01	0,92	6,58E-02	1,47	9,40E-01	1,06	1,94E-02	13,34	5,00E-02	0,99	3,21E-01	1,35
P10909	CLUS_HUMAN Clusterin	3	3	1,00E+00	1,00	8,10E-01	1,12	1,00E+00	1,00	2,11E-01	1,66	2,17E-03	1,00	8,06E-01	1,35
P04181	OAT_HUMAN Ornithine aminotransferase, mitochondrial	6	6	7,36E-01	1,80	6,57E-01	0,90	1,00E+00	1,00	1,64E-02	12,03	4,29E-01	0,81	2,25E-01	1,35
P61009	SPCS3_HUMAN Signal peptidase complex subunit 3	2	2	3,11E-01	0,44	9,65E-01	1,03	4,41E-02	3,34	6,78E-01	0,79	1,19E-01	0,98	8,11E-01	1,35

P48307	TFPI2_HUMAN Tissue factor pathway inhibitor 2	3	3	3,23E-02	0,04	5,77E-01	1,18	1,00E+00	1,00	1,00E+00	1,00	2,50E-03	1,00	7,91E-01	1,34
P00338	LDHA_HUMAN L-lactate dehydrogenase A chain	21	20	3,80E-01	0,56	3,10E-01	1,09	4,64E-01	2,73	3,59E-02	18,30	2,40E-03	1,00	5,13E-02	1,34
Q6P587	FAHD1_HUMAN Acylpyruvase FAHD1, mitochondrial	2	2	4,91E-01	1,98	4,98E-01	0,49	1,00E+00	1,00	4,17E-02	3,44	1,55E-01	0,97	7,40E-01	1,33
P00505	AATM_HUMAN Aspartate aminotransferase, mitochondrial	10	10	3,08E-01	0,16	8,50E-01	1,04	8,71E-01	1,11	2,27E-05	147,86	2,72E-02	0,99	1,41E-01	1,33
Q96FW1	OTUB1_HUMAN Ubiquitin thioesterase OTUB1	4	4	3,54E-01	0,25	6,99E-01	1,07	1,00E+00	1,00	3,03E-02	11,42	5,49E-02	0,99	5,99E-01	1,33
P14625	ENPL_HUMAN Endoplasmin	56	53	1,52E-01	0,30	1,24E-02	1,22	1,29E-01	3,24	2,37E-05	16,94	2,04E-02	0,99	1,11E-01	1,32
P62987	RL40_HUMAN Ubiquitin-60S ribosomal protein L40	5	5	2,28E-01	0,26	6,42E-01	1,10	7,57E-01	1,77	1,50E-03	5,50	2,75E-02	0,99	2,64E-01	1,32
P40925	MDHC_HUMAN Malate dehydrogenase, cytoplasmic	14	14	1,87E-01	0,14	9,60E-01	0,99	8,57E-01	0,73	2,33E-05	363,28	7,08E-04	1,00	4,21E-01	1,31
P26038	MOES_HUMAN Moesin	45	35	2,04E-01	0,09	3,37E-01	0,84	1,42E-01	2,67	3,27E-05	22,15	3,59E-03	1,00	7,85E-02	1,31
P31930	QCR1_HUMAN Cytochrome b-c1 complex subunit 1, mitochondrial	6	6	2,60E-01	0,17	6,18E-01	1,10	1,00E+00	1,00	3,47E-06	92,01	1,77E-03	1,00	5,01E-01	1,30
Q15393	SF3B3_HUMAN Splicing factor 3B subunit 3	5	5	7,42E-01	1,73	9,41E-01	0,96	3,92E-01	3,31	2,10E-01	2,24	2,07E-02	0,99	7,07E-01	1,30
P62424	RL7A_HUMAN 60S ribosomal protein L7a	9	9	7,37E-02	0,04	3,60E-01	1,15	7,04E-02	3,14	1,54E-04	61,34	9,99E-04	1,00	3,26E-01	1,29
Q9H3N1	TMX1_HUMAN Thioredoxin-related transmembrane protein 1	4	4	9,08E-01	0,85	9,89E-01	1,00	2,58E-01	0,22	2,02E-04	22,27	1,09E-01	0,98	6,72E-02	1,29
Q969V3	NCLN_HUMAN Nicalin	6	6	4,26E-01	3,39	7,74E-01	0,74	8,41E-01	1,57	1,11E-02	252,40	8,14E-01	0,59	4,17E-01	1,28

P13639	EF2_HUMAN Elongation factor 2	41	41	1,72E-01	0,14	2,79E-01	1,09	1,82E-01	3,49	3,23E-04	71,08	1,11E-03	1,00	5,08E-02	1,28
P17066	HSP76_HUMAN Heat shock 70 kDa protein 6	5	1	3,23E-01	0,50	4,81E-01	1,16	5,87E-02	22,80	1,96E-03	79,78	5,81E-02	0,99	5,75E-02	1,27
P36222	CH3L1_HUMAN Chitinase-3-like protein 1	10	10	1,47E-01	0,45	7,88E-01	1,22	9,38E-02	2,69	5,85E-04	45,15	3,78E-02	0,99	8,04E-01	1,27
P10809	CH60_HUMAN 60 kDa heat shock protein, mitochondrial	29	29	1,95E-01	0,25	3,80E-01	1,20	7,79E-01	1,40	6,53E-05	244,95	1,10E-04	1,00	6,40E-02	1,27
P06454	PTMA_HUMAN Prothymosin alpha	2	2	3,60E-01	0,24	2,01E-01	1,27	9,25E-01	0,91	1,09E-03	38,13	2,97E-03	1,00	1,67E-01	1,26
P62851	RS25_HUMAN 40S ribosomal protein S25	3	3	2,39E-01	0,10	1,10E-01	1,23	1,00E+00	1,00	4,72E-05	76,05	1,44E-02	1,00	6,67E-02	1,25
P23526	SAHH_HUMAN Adenosylhomocysteinase	10	10	2,38E-01	0,17	7,39E-01	0,88	4,15E-01	0,40	4,15E-02	11,38	3,38E-03	1,00	6,03E-01	1,25
P52566	GDIR2_HUMAN Rho GDP-dissociation inhibitor 2	2	2	1,00E+00	1,00	8,72E-01	1,06	1,00E+00	1,00	2,87E-05	54,60	1,00E+00	0,50	6,08E-01	1,25
P05556	ITB1_HUMAN Integrin beta-1	20	19	3,14E-01	0,38	6,97E-01	1,07	9,82E-02	4,43	5,82E-03	7,86	1,52E-04	1,00	8,38E-02	1,25
Q13488	VPP3_HUMAN V-type proton ATPase 116 kDa subunit a isoform 3	7	7	3,01E-01	2,82	9,71E-01	1,02	1,01E-01	2,14	1,06E-03	66,89	8,80E-02	0,98	7,70E-01	1,25
P68371	TBB4B_HUMAN Tubulin beta-4B chain	27	1	1,35E-01	0,19	3,86E-01	1,09	1,71E-01	7,60	3,87E-05	49,40	2,30E-03	1,00	4,29E-02	1,24
P78417	GSTO1_HUMAN Glutathione S-transferase omega-1	11	11	4,27E-01	0,25	3,13E-01	1,23	5,95E-01	1,34	4,99E-07	330,77	2,32E-06	1,00	5,75E-01	1,24
P69905	HBA_HUMAN Hemoglobin subunit alpha	6	3	4,56E-02	5,20	4,90E-01	1,39	1,70E-02	3,50	8,65E-01	0,94	7,65E-02	0,98	7,05E-01	1,24
P13796	PLSL_HUMAN Plastin-2	34	26	1,82E-01	0,11	1,07E-01	0,67	1,40E-01	21,33	1,94E-04	185,92	2,08E-02	0,99	3,81E-01	1,24
P09525	ANXA4_HUMAN Annexin A4	13	13	4,22E-01	0,26	4,25E-03	0,70	1,00E+00	1,00	1,36E-07	128,18	4,90E-07	1,00	6,99E-01	1,23
Q5JRX3	PREP_HUMAN Presequence	5	5	2,68E-	0,59	8,13E-	0,84	1,00E+	1,00	2,03E-	4,09	2,96E-	0,99	8,54E-	1,23

	protease, mitochondrial			01		01		00		01		02		01	
P46783	RS10_HUMAN 40S ribosomal protein S10	4	4	1,12E-01	0,07	3,14E-01	1,48	4,05E-01	3,58	3,90E-02	11,54	7,34E-01	0,63	4,08E-01	1,23
Q08211	DHX9_HUMAN ATP-dependent RNA helicase A	15	15	2,27E-02	0,10	3,48E-01	0,52	9,88E-01	0,99	1,80E-01	2,00	5,10E-01	0,76	3,03E-01	1,23
Q15008	PSMD6_HUMAN 26S proteasome non-ATPase regulatory subunit 6	8	8	2,39E-01	0,15	3,92E-01	0,72	8,52E-01	0,81	9,19E-02	11,69	3,13E-01	0,89	7,37E-01	1,23
Q9H3P7	GCP60_HUMAN Golgi resident protein GCP60	6	6	6,01E-01	0,44	6,00E-01	1,07	1,00E+00	1,00	7,94E-05	16,27	9,28E-05	1,00	5,11E-01	1,23
P00918	CAH2_HUMAN Carbonic anhydrase 2	6	6	6,27E-01	0,68	9,93E-01	1,01	9,28E-01	0,95	1,15E-05	206,93	3,45E-02	0,99	5,41E-01	1,22
P04075	ALDOA_HUMAN Fructose-bisphosphate aldolase A	27	27	1,89E-01	0,24	7,38E-01	1,03	7,64E-02	16,00	4,92E-04	12,19	2,19E-03	1,00	4,15E-02	1,22
Q9BS26	ERP44_HUMAN Endoplasmic reticulum resident protein 44	9	9	5,70E-01	0,39	4,07E-01	1,29	3,09E-01	0,21	7,63E-03	11,55	1,68E-02	0,99	2,93E-01	1,22
P27797	CALR_HUMAN Calreticulin	23	23	6,86E-01	0,55	1,81E-01	0,82	2,15E-01	0,27	1,88E-04	102,50	4,55E-02	0,99	2,11E-01	1,22
P54920	SNAA_HUMAN Alpha-soluble NSF attachment protein	6	5	5,37E-01	0,34	2,33E-01	0,82	4,98E-01	0,23	1,07E-04	62,25	4,52E-02	0,99	3,12E-01	1,21
O00231	PSD11_HUMAN 26S proteasome non-ATPase regulatory subunit 11	17	17	2,37E-01	0,24	7,72E-01	0,91	1,23E-01	3,94	1,01E-02	11,84	3,95E-01	0,83	3,27E-01	1,20
P43304	GPDM_HUMAN Glycerol-3-phosphate dehydrogenase, mitochondrial	8	8	1,75E-01	0,63	9,78E-01	1,01	9,36E-01	0,95	3,46E-03	16,01	1,52E-02	1,00	7,95E-01	1,20
P32969	RL9_HUMAN 60S ribosomal protein L9	9	9	3,44E-01	0,15	5,91E-01	1,20	7,87E-02	5,15	5,73E-04	27,47	8,19E-02	0,98	5,05E-01	1,20
P29373	RABP2_HUMAN Cellular retinoic acid-binding protein 2	5	5	1,49E-01	0,53	4,29E-01	0,69	1,00E+00	1,00	1,92E-03	33,31	2,37E-02	0,99	7,57E-01	1,20

Q15907	RB11B_HUMAN Ras-related protein Rab-11B	9	9	3,20E-01	0,17	7,87E-01	0,93	7,71E-01	1,49	1,03E-02	26,71	5,01E-04	1,00	3,86E-01	1,20
P61026	RAB10_HUMAN Ras-related protein Rab-10	3	2	1,81E-01	0,14	1,46E-01	0,66	5,55E-01	2,17	1,25E-02	20,30	6,57E-01	0,67	5,35E-01	1,19
Q9NR30	DDX21_HUMAN Nucleolar RNA helicase 2	3	3	9,59E-01	1,07	9,90E-01	1,01	5,41E-01	0,52	9,65E-03	9,63	6,06E-01	0,70	9,06E-01	1,18
P55072	TERA_HUMAN Transitional endoplasmic reticulum ATPase	41	41	2,42E-01	0,19	9,20E-01	0,99	1,83E-01	0,22	5,90E-04	100,26	2,57E-03	1,00	1,12E-01	1,17
Q13200	PSMD2_HUMAN 26S proteasome non-ATPase regulatory subunit 2	18	18	3,30E-01	0,30	3,58E-01	0,81	7,45E-01	0,79	1,93E-02	3,40	4,87E-04	1,00	2,46E-01	1,17
Q9BSJ8	ESYT1_HUMAN Extended synaptotagmin-1	12	12	3,01E-01	0,65	5,86E-01	1,13	1,75E-02	16,51	2,72E-03	21,84	3,27E-01	0,88	5,02E-01	1,17
P04844	RPN2_HUMAN Dolichyl-diphosphooligosaccharide--protein glycosyltransferase subunit 2	18	18	3,53E-01	0,39	8,42E-01	0,95	8,30E-01	1,51	4,71E-03	104,02	1,33E-02	1,00	5,73E-01	1,16
P08865	RSSA_HUMAN 40S ribosomal protein SA	15	15	1,87E-01	0,19	5,31E-02	1,30	3,38E-01	3,38	1,22E-07	169,98	2,66E-03	1,00	2,17E-01	1,16
P35268	RL22_HUMAN 60S ribosomal protein L22	5	5	2,08E-01	0,08	5,56E-01	1,05	1,00E+00	1,00	2,04E-03	29,37	6,68E-08	1,00	1,49E-01	1,16
P61247	RS3A_HUMAN 40S ribosomal protein S3a	14	13	1,36E-01	0,08	7,56E-01	0,94	8,47E-01	0,83	3,53E-01	2,77	3,63E-02	0,99	7,27E-01	1,15
P16671	CD36_HUMAN Platelet glycoprotein 4	6	6	6,66E-01	1,37	8,02E-01	1,11	1,00E+00	1,00	7,39E-04	22,29	1,00E+00	0,50	9,06E-01	1,15
P07858	CATB_HUMAN Cathepsin B	11	11	3,12E-01	0,23	9,20E-01	0,98	2,19E-01	9,62	2,13E-03	228,06	1,43E-02	1,00	6,27E-01	1,15
P07437	TBB5_HUMAN Tubulin beta chain	25	5	1,69E-01	0,12	8,04E-01	0,98	4,66E-02	22,42	3,62E-03	237,19	4,26E-03	1,00	9,37E-02	1,14
P63244	RACK1_HUMAN Receptor of activated protein C kinase 1	9	9	2,44E-01	0,28	3,14E-01	1,23	7,25E-01	0,78	8,49E-03	29,18	2,33E-01	0,93	6,31E-01	1,14

P60903	S10AA_HUMAN Protein S100-A10	4	4	2,02E-01	0,13	1,23E-01	0,44	1,00E+00	1,00	6,14E-01	1,67	2,33E-02	0,99	6,06E-01	1,13
P62750	RL23A_HUMAN 60S ribosomal protein L23a	7	7	8,81E-01	1,30	2,06E-01	1,25	1,00E+00	1,00	2,25E-02	21,48	7,14E-03	1,00	4,71E-01	1,13
P80723	BASP1_HUMAN Brain acid soluble protein 1	6	6	7,17E-01	0,55	5,65E-01	1,16	7,30E-01	0,71	5,79E-02	5,31	1,88E-03	1,00	4,99E-01	1,13
P09960	LKHA4_HUMAN Leukotriene A-4 hydrolase	15	15	4,30E-01	0,28	4,16E-01	1,15	4,59E-01	3,35	2,41E-04	177,39	8,10E-05	1,00	2,12E-01	1,13
Q15084	PDIA6_HUMAN Protein disulfide-isomerase A6	20	20	2,78E-01	0,21	8,40E-01	0,97	3,09E-02	15,71	3,51E-05	227,97	7,43E-03	1,00	6,14E-02	1,13
P20340	RAB6A_HUMAN Ras-related protein Rab-6A	7	7	2,92E-01	0,11	5,22E-01	0,96	9,26E-01	0,87	1,37E-02	63,65	6,88E-03	1,00	4,06E-01	1,13
Q9NUJ1	ABHDA_HUMAN Mycophenolic acid acyl-glucuronide esterase, mitochondrial	4	4	4,20E-01	0,74	6,31E-01	0,72	1,68E-01	3,24	8,53E-02	1,68	4,13E-02	0,99	6,06E-01	1,13
P45974	UBP5_HUMAN Ubiquitin carboxyl-terminal hydrolase 5	10	10	1,38E-01	0,09	4,20E-01	0,72	6,09E-02	2,85	1,35E-01	1,87	9,38E-05	1,00	7,53E-01	1,12
P51149	RAB7A_HUMAN Ras-related protein Rab-7a	13	13	8,65E-01	0,77	9,37E-01	1,01	7,59E-01	1,65	4,51E-04	220,40	9,32E-02	0,98	4,18E-01	1,12
P31943	HNRH1_HUMAN Heterogeneous nuclear ribonucleoprotein H	11	7	2,96E-01	0,15	6,20E-01	0,93	8,80E-01	0,89	6,88E-06	99,97	3,79E-03	1,00	5,82E-01	1,12
P68104	EF1A1_HUMAN Elongation factor 1-alpha 1	20	20	1,37E-01	0,26	1,42E-01	1,13	1,14E-01	22,32	2,00E-03	8,18	1,20E-03	1,00	2,30E-02	1,12
Q9UBS4	DJB11_HUMAN DnaJ homolog subfamily B member 11	3	3	1,37E-01	0,15	8,45E-02	0,84	8,15E-01	1,25	7,73E-02	8,24	4,37E-02	0,99	7,37E-01	1,12
Q00325	MPCP_HUMAN Phosphate carrier protein, mitochondrial	4	4	2,93E-01	0,19	8,82E-01	1,03	2,80E-02	22,70	6,39E-03	78,10	3,85E-02	0,99	1,74E-01	1,12
O43707	ACTN4_HUMAN Alpha-actinin-4	52	29	1,72E-01	0,07	6,47E-03	1,58	6,02E-01	0,71	6,78E-04	46,84	3,54E-03	1,00	5,54E-01	1,12

P37802	TAGL2_HUMAN Transgelin-2	15	14	5,07E-01	0,34	6,25E-01	0,92	9,46E-02	5,61	3,92E-05	47,24	8,84E-03	1,00	2,62E-01	1,12
P09972	ALDOC_HUMAN Fructose-bisphosphate aldolase C	3	3	5,49E-01	0,19	6,79E-02	0,82	1,37E-01	10,51	8,48E-02	19,92	7,76E-04	1,00	3,80E-01	1,12
P62820	RAB1A_HUMAN Ras-related protein Rab-1A	10	5	4,45E-01	0,18	2,98E-01	0,80	8,91E-01	1,17	1,37E-02	26,46	1,65E-02	0,99	7,71E-01	1,12
Q02978	M2OM_HUMAN Mitochondrial 2-oxoglutarate/malate carrier protein	2	2	1,90E-01	0,14	5,75E-01	1,15	2,18E-01	4,57	1,17E-06	69,27	6,79E-07	1,00	3,75E-01	1,12
O00754	MA2B1_HUMAN Lysosomal alpha-mannosidase	6	6	6,98E-02	5,21	5,01E-02	0,35	3,67E-01	0,48	1,25E-03	41,24	8,90E-01	0,55	9,36E-01	1,11
Q13885	TBB2A_HUMAN Tubulin beta-2A chain	25	5	8,32E-02	0,08	7,21E-01	1,10	7,84E-01	0,77	3,87E-04	19,52	1,16E-02	1,00	4,70E-01	1,11
P60174	TPIS_HUMAN Triosephosphate isomerase	18	18	2,87E-01	0,36	5,34E-01	1,13	4,41E-02	28,15	1,69E-05	205,87	9,32E-04	1,00	3,31E-01	1,11
P25788	PSA3_HUMAN Proteasome subunit alpha type-3	5	5	2,95E-01	0,15	5,94E-01	1,06	7,17E-01	1,66	1,27E-02	11,20	8,52E-07	1,00	2,61E-01	1,11
P11142	HSP7C_HUMAN Heat shock cognate 71 kDa protein	38	29	3,15E-01	0,33	2,03E-01	1,03	6,79E-01	1,82	1,20E-02	42,39	1,20E-03	1,00	4,75E-01	1,10
P50552	VASP_HUMAN Vasodilator-stimulated phosphoprotein	4	4	1,61E-01	0,48	5,83E-01	0,64	6,94E-01	1,28	1,28E-01	1,61	2,76E-02	0,99	9,52E-01	1,10
Q16698	DECR_HUMAN 2,4-dienoyl-CoA reductase, mitochondrial	7	7	8,27E-01	1,27	2,41E-01	0,85	5,61E-01	0,57	2,82E-06	96,46	9,67E-02	0,98	8,31E-01	1,10
P40926	MDHM_HUMAN Malate dehydrogenase, mitochondrial	16	16	2,40E-01	0,11	4,88E-01	1,11	2,67E-01	3,70	5,74E-05	36,25	4,67E-03	1,00	6,63E-01	1,09
P29218	IMPA1_HUMAN Inositol monophosphatase 1	3	3	2,35E-01	0,38	4,39E-01	0,87	1,00E+00	1,00	2,14E-01	3,62	1,25E-02	1,00	4,46E-01	1,09
P46781	RS9_HUMAN 40S ribosomal protein S9	8	8	6,28E-01	1,90	9,85E-01	1,01	1,00E+00	1,00	5,64E-02	8,15	4,86E-02	0,99	6,05E-01	1,09
P11047	LAMC1_HUMAN Laminin subunit gamma-1	13	13	5,75E-01	0,40	4,44E-01	0,72	9,20E-01	0,92	5,21E-03	13,00	2,11E-02	0,99	7,91E-01	1,09

P40227	TCPZ_HUMAN T-complex protein 1 subunit zeta	17	14	5,56E-01	0,58	8,56E-01	0,97	1,00E-01	0,10	2,79E-02	16,14	5,12E-03	1,00	5,93E-01	1,09
Q04917	1433F_HUMAN 14-3-3 protein eta	10	9	9,07E-01	0,81	5,28E-01	0,84	4,16E-01	0,25	8,95E-06	60,55	2,36E-03	1,00	7,89E-01	1,09
P51571	SSRD_HUMAN Translocon-associated protein subunit delta	5	5	2,07E-01	0,11	9,12E-01	1,04	6,31E-01	0,59	3,04E-01	3,39	1,64E-02	0,99	7,47E-01	1,09
P32455	GBP1_HUMAN Guanylate-binding protein 1	6	5	4,54E-02	0,06	8,14E-01	1,05	1,00E+00	1,00	1,00E+00	1,00	1,40E-01	0,97	9,55E-01	1,08
O00299	CLIC1_HUMAN Chloride intracellular channel protein 1	12	12	1,76E-01	0,06	1,57E-01	1,21	6,69E-01	2,37	1,99E-03	153,70	4,78E-03	1,00	4,50E-01	1,08
P61978	HNRPK_HUMAN Heterogeneous nuclear ribonucleoprotein K	16	16	1,36E-01	0,08	9,37E-01	1,02	6,62E-01	1,59	2,66E-03	25,70	1,13E-03	1,00	5,93E-01	1,08
P28066	PSA5_HUMAN Proteasome subunit alpha type-5	11	11	4,41E-01	0,34	4,86E-02	0,48	2,51E-01	2,99	2,02E-03	12,44	3,78E-01	0,85	8,64E-01	1,08
P07737	PROF1_HUMAN Profilin-1	13	10	2,32E-01	0,25	5,11E-01	1,20	1,72E-01	9,30	4,91E-03	53,20	1,14E-03	1,00	7,41E-01	1,08
P62241	RS8_HUMAN 40S ribosomal protein S8	5	5	5,49E-01	0,27	8,61E-01	1,03	9,75E-01	1,05	4,22E-06	87,05	5,64E-02	0,99	6,89E-01	1,08
Q9BQE5	APOL2_HUMAN Apolipoprotein L2	4	4	5,32E-01	0,59	3,26E-01	0,85	1,00E+00	1,00	3,53E-04	15,78	7,99E-03	1,00	7,48E-01	1,08
P62140	PP1B_HUMAN Serine/threonine-protein phosphatase PP1-beta catalytic subunit	13	5	1,05E-01	0,12	6,41E-01	1,17	1,00E+00	1,00	1,58E-02	9,02	2,30E-03	1,00	8,40E-01	1,07
P35237	SPB6_HUMAN Serpin B6	14	13	3,81E-01	0,46	3,05E-01	0,61	1,22E-01	9,76	1,76E-02	10,28	1,25E-01	0,97	7,25E-01	1,07
P38571	LICH_HUMAN Lysosomal acid lipase/cholesteryl ester hydrolase	4	4	7,11E-01	1,12	4,97E-01	0,67	5,48E-01	1,37	2,86E-02	7,44	1,00E+00	0,50	8,33E-01	1,07
P60709	ACTB_HUMAN Actin, cytoplasmic 1	31	1	1,83E-01	0,41	1,13E-01	1,29	1,85E-02	7,04	3,96E-04	8,85	2,82E-03	1,00	5,34E-01	1,07

P14618	KPYM_HUMAN Pyruvate kinase PKM	40	39	8,94E-02	0,37	5,70E-01	0,93	1,30E-01	7,09	4,66E-03	46,88	8,96E-03	1,00	6,21E-01	1,07
P27348	1433T_HUMAN 14-3-3 protein theta	12	10	4,85E-01	0,28	7,80E-01	1,02	3,78E-02	2,74	1,73E-04	36,13	4,61E-03	1,00	7,60E-01	1,06
P10321	HLAC_HUMAN HLA class I histocompatibility antigen, C alpha chain	6	2	5,78E-01	0,53	8,63E-01	0,98	1,00E+00	1,00	1,84E-06	152,98	5,60E-02	0,99	8,76E-01	1,06
Q15365	PCBP1_HUMAN Poly(rC)-binding protein 1	9	7	2,80E-01	0,13	6,94E-01	1,11	2,21E-01	3,72	3,72E-02	18,39	1,00E-03	1,00	5,48E-01	1,06
P60468	SC61B_HUMAN Protein transport protein Sec61 subunit beta	3	3	2,03E-01	0,14	4,77E-01	1,13	7,22E-01	0,70	2,26E-02	6,63	5,32E-06	1,00	7,03E-01	1,06
P11413	G6PD_HUMAN Glucose-6-phosphate 1-dehydrogenase	21	21	6,76E-03	0,06	5,32E-01	0,89	1,66E-02	36,82	1,25E-03	107,23	3,37E-05	1,00	8,20E-01	1,06
P13693	TCTP_HUMAN Translationally-controlled tumor protein	5	5	2,49E-01	0,18	6,41E-01	0,88	3,04E-01	1,35	2,64E-02	18,49	2,42E-02	0,99	7,95E-01	1,06
P60842	IF4A1_HUMAN Eukaryotic initiation factor 4A-I	20	11	1,89E-01	0,12	6,81E-01	0,95	2,92E-01	0,34	7,68E-03	37,64	7,02E-03	1,00	4,77E-01	1,05
Q92945	FUBP2_HUMAN Far upstream element-binding protein 2	10	9	6,43E-01	0,49	2,75E-01	1,13	8,09E-01	0,76	4,04E-02	9,70	7,11E-02	0,99	7,19E-01	1,05
P55084	ECHB_HUMAN Trifunctional enzyme subunit beta, mitochondrial	14	14	2,68E-01	0,25	4,13E-01	0,61	2,61E-01	1,80	3,51E-04	96,75	3,37E-02	0,99	9,53E-01	1,05
Q9Y5K8	VATD_HUMAN V-type proton ATPase subunit D	3	3	6,10E-01	0,69	7,57E-01	1,28	5,70E-01	0,51	6,07E-03	48,90	9,13E-01	0,54	7,68E-01	1,05
Q01518	CAP1_HUMAN Adenylyl cyclase-associated protein 1	28	27	2,36E-01	0,13	6,74E-02	0,89	7,02E-01	2,01	2,89E-03	15,89	4,55E-03	1,00	6,39E-01	1,05
P25705	ATPA_HUMAN ATP synthase subunit alpha, mitochondrial	28	27	3,18E-01	0,36	8,62E-01	0,97	7,84E-02	4,83	3,81E-05	57,44	7,90E-03	1,00	5,27E-01	1,05

Q13177	PAK2_HUMAN Serine/threonine-protein kinase PAK 2	8	8	3,56E-01	0,36	2,56E-01	0,38	1,00E+00	1,00	1,64E-02	6,47	1,56E-03	1,00	8,33E-01	1,04
P40939	ECHA_HUMAN Trifunctional enzyme subunit alpha, mitochondrial	23	23	4,44E-01	0,35	8,75E-01	0,96	2,06E-01	5,74	5,60E-05	247,18	9,01E-03	1,00	8,79E-01	1,04
P39656	OST48_HUMAN Dolichyl-diphosphooligosaccharide--protein glycosyltransferase 48 kDa subunit	10	10	8,38E-01	0,72	5,40E-01	0,81	1,00E+00	1,00	2,32E-06	57,28	1,82E-02	0,99	5,39E-01	1,04
Q9Y4P3	TBL2_HUMAN Transducin beta-like protein 2	3	3	2,33E-01	0,17	3,10E-01	1,15	1,00E+00	1,00	1,97E-02	6,55	2,07E-06	1,00	6,53E-01	1,04
Q96HE7	ERO1A_HUMAN ERO1-like protein alpha OS	5	5	2,86E-01	0,19	3,25E-01	1,16	9,83E-01	1,02	1,20E-03	23,62	1,97E-05	1,00	8,03E-01	1,04
O60701	UGDH_HUMAN UDP-glucose 6-dehydrogenase	17	17	1,85E-01	0,09	8,06E-01	1,04	3,72E-02	5,21	3,53E-01	0,54	5,27E-03	1,00	7,50E-01	1,04
P42704	LPPRC_HUMAN Leucine-rich PPR motif-containing protein, mitochondrial	22	22	5,19E-02	0,06	9,35E-02	0,63	2,86E-01	0,18	3,80E-03	48,93	2,64E-01	0,92	9,20E-01	1,04
P60660	MYL6_HUMAN Myosin light polypeptide 6	9	9	1,46E-01	0,07	5,97E-01	0,84	5,18E-01	1,43	6,22E-05	32,04	2,69E-02	0,99	8,57E-01	1,03
P46782	RS5_HUMAN 40S ribosomal protein S5	4	4	9,26E-02	0,05	3,98E-01	1,12	7,96E-01	0,76	2,26E-02	18,45	2,81E-07	1,00	8,93E-01	1,03
P23396	RS3_HUMAN 40S ribosomal protein S3	13	13	3,82E-01	0,18	2,47E-01	1,25	6,23E-01	0,68	1,03E-04	8,59	3,57E-03	1,00	9,00E-01	1,02
Q14764	MVP_HUMAN Major vault protein	39	39	3,04E-01	0,18	2,36E-01	0,87	9,29E-02	0,23	2,92E-02	13,76	1,84E-03	1,00	8,34E-01	1,02
P07237	PDIA1_HUMAN Protein disulfide-isomerase	38	37	1,33E-01	0,24	3,61E-02	0,91	4,34E-01	3,09	8,90E-04	33,61	3,38E-03	1,00	8,05E-01	1,02
P62805	H4_HUMAN Histone H4	12	12	2,23E-01	0,32	2,13E-03	2,00	2,40E-01	8,56	4,66E-04	5,33	3,13E-02	0,99	9,23E-01	1,02
P21980	TGM2_HUMAN Protein-glutamine gamma-	26	26	7,37E-01	0,69	2,66E-02	1,56	2,81E-01	8,64	1,48E-02	33,99	8,28E-01	0,58	9,28E-01	1,02

	glutamyltransferase 2														
P11021	BIP_HUMAN Endoplasmic reticulum chaperone BiP	40	39	1,89E-01	0,11	2,72E-01	0,97	1,04E-01	4,58	1,90E-03	68,25	1,15E-02	1,00	8,28E-01	1,02
P21589	5NTD_HUMAN 5-nucleotidase	10	10	8,67E-01	0,74	6,30E-02	1,71	1,00E+00	1,00	1,37E-02	4,30	4,12E-02	0,99	8,99E-01	1,02
P50579	MAP2_HUMAN Methionine aminopeptidase 2	2	2	1,94E-01	0,10	2,83E-01	2,27	1,33E-01	19,51	4,48E-02	45,89	1,75E-03	1,00	8,36E-01	1,02
P35613	BASI_HUMAN Basigin	7	6	5,76E-01	0,41	3,89E-01	0,66	3,12E-01	2,60	7,69E-04	62,52	5,40E-02	0,99	9,58E-01	1,02
P04406	G3P_HUMAN Glyceraldehyde-3-phosphate dehydrogenase	23	23	1,73E-01	0,41	4,46E-01	0,93	9,89E-02	4,19	1,00E-03	7,25	2,29E-03	1,00	9,42E-01	1,01
P07355	ANXA2_HUMAN Annexin A2	40	40	1,99E-01	0,42	5,03E-02	0,84	7,38E-02	10,51	9,18E-05	8,41	2,38E-04	1,00	8,47E-01	1,01
P26373	RL13_HUMAN 60S ribosomal protein L13	8	8	1,81E-01	0,06	1,13E-01	1,26	2,36E-01	3,11	3,63E-04	52,30	4,36E-02	0,99	9,65E-01	1,01
Q9NTK5	OLA1_HUMAN	7	6	4,47E-01	0,30	5,98E-01	0,85	3,30E-01	0,32	7,44E-03	10,61	1,23E-03	1,00	9,36E-01	1,01
P59998	ARPC4_HUMAN Actin-related protein 2/3 complex subunit 4	6	6	5,03E-01	0,25	1,94E-01	1,20	3,93E-01	3,79	5,67E-04	112,94	7,37E-06	1,00	9,80E-01	1,01
P62258	1433E_HUMAN 14-3-3 protein epsilon	19	18	4,60E-01	0,32	4,93E-01	0,89	6,29E-01	0,57	5,21E-05	80,04	3,20E-04	1,00	9,82E-01	1,00
O75915	PRAF3_HUMAN PRA1 family protein 3	3	3	4,22E-01	0,41	5,24E-01	0,56	4,13E-01	0,26	3,99E-02	24,67	6,11E-01	0,69	9,96E-01	1,00
P00367	DHE3_HUMAN Glutamate dehydrogenase 1, mitochondrial	16	15	6,87E-01	0,74	2,10E-01	1,16	1,69E-01	2,74	2,25E-03	51,97	9,55E-02	0,98	9,76E-01	1,00
Q14103	HNRPD_HUMAN Heterogeneous nuclear ribonucleoprotein D0	6	6	8,79E-01	0,74	7,99E-01	0,96	4,32E-02	29,41	7,42E-01	1,49	1,83E-08	1,00	9,80E-01	1,00
O00182	LEG9_HUMAN Galectin-9	4	4	1,00E+	1,00	6,98E-	0,56	1,77E-	4,67	1,08E-	145,6	1,00E+	0,50	9,96E-	1,00

				00		02		01		05	5	00		01	
Q16647	PTGIS_HUMAN Prostacyclin synthase	2	2	1,32E-01	0,44	2,31E-02	0,24	1,00E+00	1,00	1,00E+00	1,00	1,02E-02	1,00	1,00E+00	1,00
Q6VY07	PACS1_HUMAN Phosphofurin acidic cluster sorting protein 1	3	3	1,66E-01	0,29	2,38E-02	6,50	1,00E+00	1,00	1,00E+00	1,00	1,00E+00	0,50	1,00E+00	1,00
Q9Y2G5	OFUT2_HUMAN GDP-fucose protein O-fucosyltransferase 2	2	2	2,09E-01	0,29	2,53E-02	2,40	9,53E-01	0,95	5,81E-01	0,66	1,00E+00	0,50	1,00E+00	1,00
Q9Y6U3	ADSV_HUMAN Adseverin	2	2	2,83E-01	0,59	4,13E-02	0,45	1,00E+00	1,00	1,00E+00	1,00	1,00E+00	0,50	1,00E+00	1,00
Q9H9A6	LRC40_HUMAN Leucine-rich repeat-containing protein 40	2	2	3,07E-01	0,42	1,63E-02	18,60	1,00E+00	1,00	1,00E+00	1,00	1,00E+00	0,50	1,00E+00	1,00
O43681	GET3_HUMAN ATPase GET3	3	2	3,47E-01	0,67	7,58E-01	0,77	1,34E-01	3,78	1,00E+00	1,00	7,71E-02	0,98	1,00E+00	1,00
O00160	MYO1F_HUMAN Unconventional myosin-If	3	2	3,63E-01	0,51	8,32E-01	0,93	1,00E+00	1,00	3,63E-02	3,94	1,00E+00	0,50	1,00E+00	1,00
P07099	HYEP_HUMAN Epoxide hydrolase 1	4	4	7,16E-01	0,82	7,12E-01	0,79	1,00E+00	1,00	3,79E-02	8,99	1,00E+00	0,50	1,00E+00	1,00
Q9H2U2	IPYR2_HUMAN Inorganic pyrophosphatase 2, mitochondrial	3	3	7,64E-01	0,75	3,60E-01	0,50	8,93E-01	0,88	2,21E-06	42,54	2,96E-02	0,99	1,00E+00	1,00
P48147	PPCE_HUMAN Prolyl endopeptidase	3	3	7,95E-01	0,54	6,89E-01	1,61	8,88E-01	1,37	8,55E-01	1,36	1,00E+00	0,50	1,00E+00	1,00
P61225	RAP2B_HUMAN Ras-related protein Rap-2b	4	4	1,00E+00	1,00	9,78E-01	1,02	7,36E-01	0,73	3,72E-04	24,73	1,00E+00	0,50	1,00E+00	1,00
O43914	TYOBP_HUMAN TYRO protein tyrosine kinase-binding protein	2	2	1,00E+00	1,00	8,35E-01	0,95	1,00E+00	1,00	2,54E-06	120,68	1,00E+00	0,50	1,00E+00	1,00
P15090	FABP4_HUMAN Fatty acid-binding protein, adipocyte	2	2	1,00E+00	1,00	4,13E-01	0,49	2,85E-01	0,12	2,48E-03	0,02	1,00E+00	0,50	1,00E+00	1,00

Q9UHL4	DPP2_HUMAN Dipeptidyl peptidase 2	2	2	1,00E+00	1,00	3,44E-01	0,58	1,00E+00	1,00	1,33E-02	6,38	1,00E+00	0,50	1,00E+00	1,00
Q9UI08	EVL_HUMAN Ena/VASP-like protein	2	2	1,00E+00	1,00	1,00E+00	1,00	1,00E+00	1,00	1,97E-02	19,17	1,00E+00	0,50	1,00E+00	1,00
P55209	NP1L1_HUMAN Nucleosome assembly protein 1-like 1	6	5	4,76E-01	0,23	1,81E-01	0,60	9,51E-01	1,04	2,27E-02	12,76	1,04E-03	1,00	9,89E-01	1,00
O15511	ARPC5_HUMAN Actin-related protein 2/3 complex subunit 5	3	3	7,62E-01	1,59	5,93E-01	1,19	3,31E-01	3,03	1,86E-06	304,40	6,34E-03	1,00	9,59E-01	1,00
O75348	VATG1_HUMAN V-type proton ATPase subunit G 1	3	3	2,65E-01	0,16	8,90E-01	1,05	2,20E-01	7,53	2,45E-04	81,23	8,20E-05	1,00	9,85E-01	0,99
Q9Y5M8	SRPRB_HUMAN Signal recognition particle receptor subunit beta	7	7	8,74E-01	1,33	6,97E-01	0,94	3,03E-01	0,28	1,45E-02	12,52	8,33E-03	1,00	9,50E-01	0,99
P05023	AT1A1_HUMAN Sodium/potassium-transporting ATPase subunit alpha-1	18	18	1,54E-01	0,09	9,78E-01	1,00	1,75E-01	3,76	5,58E-07	248,78	8,75E-03	1,00	9,72E-01	0,99
P61981	1433G_HUMAN 14-3-3 protein gamma	12	9	5,76E-01	0,34	1,30E-01	0,85	1,00E+00	1,00	2,78E-05	91,45	6,85E-03	1,00	9,54E-01	0,99
P11310	ACADM_HUMAN Medium-chain specific acyl-CoA dehydrogenase, mitochondrial	6	6	6,22E-02	0,13	6,86E-01	0,80	1,00E+00	1,00	6,52E-04	45,32	6,16E-03	1,00	9,94E-01	0,99
P50990	TCPQ_HUMAN T-complex protein 1 subunit theta	26	26	3,94E-01	0,25	3,26E-01	0,74	1,05E-01	6,66	1,80E-04	21,27	6,11E-06	1,00	9,66E-01	0,99
P29401	TKT_HUMAN Transketolase	23	23	6,85E-02	0,04	7,39E-01	0,93	2,98E-01	6,09	9,22E-04	180,69	7,46E-04	1,00	9,61E-01	0,99
P12814	ACTN1_HUMAN Alpha-actinin-1	63	37	1,19E-01	0,17	2,40E-01	1,12	4,64E-02	4,24	1,15E-06	35,93	4,90E-03	1,00	9,03E-01	0,99
Q6DD88	ATLA3_HUMAN Atlastin-3	11	11	2,86E-01	0,22	1,88E-02	0,56	2,06E-01	1,43	3,31E-02	9,39	9,96E-03	1,00	8,85E-01	0,98
P62841	RS15_HUMAN 40S ribosomal protein S15	2	2	1,30E-01	9,52	3,46E-01	0,59	4,01E-04	0,05	7,00E-01	0,79	1,58E-02	1,00	9,23E-01	0,98

P35579	MYH9_HUMAN Myosin-9	143	127	8,25E-02	0,07	2,01E-01	1,12	5,36E-02	8,28	8,73E-05	48,97	2,15E-02	0,99	8,02E-01	0,98
Q9Y394	DHRS7_HUMAN Dehydrogenase/reductase SDR family member 7	4	4	4,63E-01	0,36	5,50E-01	0,92	1,00E+00	1,00	3,50E-05	34,86	1,50E-01	0,97	9,35E-01	0,98
P13489	RINI_HUMAN Ribonuclease inhibitor	5	5	7,22E-01	1,77	3,90E-01	0,90	3,90E-01	5,55	2,29E-06	288,05	7,32E-01	0,63	8,75E-01	0,98
P21796	VDAC1_HUMAN Voltage-dependent anion-selective channel protein 1	10	10	6,62E-01	0,39	2,49E-01	0,80	8,84E-02	6,74	9,85E-04	178,49	2,32E-02	0,99	8,44E-01	0,98
P06576	ATPB_HUMAN ATP synthase subunit beta, mitochondrial	28	28	2,66E-01	0,21	4,79E-01	1,07	3,29E-02	6,43	2,29E-03	129,54	1,61E-03	1,00	6,88E-01	0,97
P63104	1433Z_HUMAN 14-3-3 protein zeta/delta	22	19	1,67E-01	0,28	8,40E-01	0,97	4,18E-02	7,02	1,64E-03	138,96	1,96E-02	0,99	8,35E-01	0,97
P23246	SFPQ_HUMAN Splicing factor, proline- and glutamine-rich	11	11	2,59E-01	0,13	6,22E-01	1,11	8,25E-01	0,83	6,91E-04	11,42	6,37E-04	1,00	8,94E-01	0,97
O75964	ATP5L_HUMAN ATP synthase subunit g, mitochondrial	5	5	1,80E-01	0,12	7,53E-01	1,11	3,64E-01	3,28	4,22E-03	54,43	9,86E-03	1,00	8,75E-01	0,97
Q9UJ70	NAGK_HUMAN N-acetyl-D-glucosamine kinase	9	9	7,65E-01	1,56	4,17E-02	1,62	5,54E-01	3,06	7,97E-04	89,39	2,44E-02	0,99	8,46E-01	0,97
Q9UI30	TR112_HUMAN Multifunctional methyltransferase subunit TRM112-like protein	3	3	4,43E-02	0,11	8,12E-02	3,15	2,13E-01	2,87	1,66E-02	15,34	5,49E-02	0,99	9,73E-01	0,97
Q9UHD8	SEPT9_HUMAN Septin-9	15	15	3,43E-01	0,17	5,58E-01	1,08	6,56E-01	0,70	8,23E-05	39,58	5,29E-02	0,99	8,63E-01	0,97
P62081	RS7_HUMAN 40S ribosomal protein S7	7	7	1,16E-01	0,08	5,13E-01	1,43	9,00E-02	5,02	1,97E-04	8,35	2,18E-02	0,99	8,64E-01	0,96
P38117	ETFB_HUMAN Electron transfer flavoprotein subunit	6	6	6,44E-01	0,45	2,93E-01	1,17	8,99E-01	0,87	1,24E-01	12,43	4,28E-06	1,00	7,30E-01	0,96

	beta														
P34897	GLYM_HUMAN Serine hydroxymethyltransferase, mitochondrial	10	10	5,92E-01	0,44	6,59E-01	1,18	9,68E-01	1,04	2,14E-01	4,07	4,51E-06	1,00	8,90E-01	0,96
P49189	AL9A1_HUMAN 4-trimethylaminobutyraldehyde dehydrogenase	13	13	2,28E-01	0,13	6,01E-02	0,55	4,63E-01	1,63	3,47E-02	14,45	6,80E-05	1,00	9,38E-01	0,96
P07384	CAN1_HUMAN Calpain-1 catalytic subunit	10	10	7,36E-01	1,50	3,50E-01	0,49	6,69E-01	0,71	4,80E-03	20,19	4,69E-02	0,99	9,58E-01	0,96
P45880	VDAC2_HUMAN Voltage-dependent anion-selective channel protein 2	6	6	2,90E-01	0,17	1,48E-01	1,16	6,32E-01	2,09	6,24E-06	231,83	1,65E-02	0,99	7,20E-01	0,96
P11279	LAMP1_HUMAN Lysosome-associated membrane glycoprotein 1	4	4	6,62E-01	0,43	3,07E-01	1,87	3,61E-01	6,33	4,48E-03	9,96	1,66E-02	0,99	6,93E-01	0,96
P34932	HSP74_HUMAN Heat shock 70 kDa protein 4	20	18	5,96E-01	0,38	5,75E-01	0,78	2,75E-01	2,85	2,12E-01	5,91	7,84E-03	1,00	9,13E-01	0,95
P78371	TCPB_HUMAN T-complex protein 1 subunit beta	26	26	1,38E-01	0,05	8,57E-01	0,99	1,13E-01	3,49	2,55E-02	5,18	4,29E-02	0,99	7,81E-01	0,95
Q99541	PLIN2_HUMAN Perilipin-2	10	10	1,25E-01	0,30	6,98E-01	0,81	3,86E-02	8,14	1,18E-03	88,15	1,47E-02	1,00	7,24E-01	0,95
P38646	GRP75_HUMAN Stress-70 protein, mitochondrial	30	29	3,66E-01	0,26	8,19E-01	1,03	5,93E-01	0,58	3,60E-04	233,85	6,57E-04	1,00	3,56E-01	0,95
P31946	1433B_HUMAN 14-3-3 protein beta/alpha	18	13	9,98E-01	1,00	5,71E-01	0,90	2,00E-01	0,30	1,65E-03	48,60	7,12E-03	1,00	8,28E-01	0,95
Q15942	ZYX_HUMAN Zyxin	14	14	1,80E-01	0,08	2,81E-03	0,73	2,38E-01	2,87	2,36E-04	98,31	2,99E-04	1,00	7,82E-01	0,95
O15460	P4HA2_HUMAN Prolyl 4-hydroxylase subunit alpha-2	18	18	4,21E-01	0,44	5,67E-01	0,85	4,72E-02	0,12	2,84E-01	0,28	7,76E-03	1,00	7,54E-01	0,95
Q14847	LASP1_HUMAN LIM and SH3 domain protein 1	10	10	1,52E-01	0,37	2,85E-01	0,81	1,65E-01	2,81	3,42E-03	2,82	3,76E-07	1,00	6,75E-01	0,94
P14780	MMP9_HUMAN Matrix	11	11	4,41E-	0,71	8,80E-	1,13	1,53E-	1,61	8,77E-	77,91	2,27E-	1,00	9,41E-	0,94

	metalloproteinase-9			01		01		01		04		03		01	
P21333	FLNA_HUMAN Filamin-A	126	115	1,44E-01	0,19	5,76E-01	1,05	5,72E-02	5,29	1,93E-03	416,97	2,12E-03	1,00	4,49E-01	0,94
P09211	GSTP1_HUMAN Glutathione S-transferase P	11	11	2,92E-01	0,12	9,56E-02	0,78	7,94E-01	1,21	1,28E-03	20,94	6,77E-03	1,00	8,47E-01	0,94
Q15717	ELAV1_HUMAN ELAV-like protein 1	5	5	3,57E-01	0,22	3,32E-01	0,79	1,00E+00	1,00	2,15E-01	5,71	1,39E-07	1,00	7,47E-01	0,94
P62277	RS13_HUMAN 40S ribosomal protein S13	7	7	1,27E-01	0,08	1,45E-01	0,57	6,13E-02	2,64	6,05E-01	2,25	2,00E-02	0,99	7,82E-01	0,94
P19105	ML12A_HUMAN Myosin regulatory light chain 12A	7	3	3,74E-01	0,21	1,39E-01	0,76	9,69E-01	1,05	1,36E-02	8,40	4,48E-02	0,99	6,28E-01	0,94
P30837	AL1B1_HUMAN Aldehyde dehydrogenase X, mitochondrial	9	8	4,39E-01	0,33	8,08E-01	0,96	9,86E-01	0,99	2,66E-01	1,68	1,36E-02	1,00	7,54E-01	0,94
P10644	KAP0_HUMAN cAMP-dependent protein kinase type I-alpha regulatory subunit	6	6	7,58E-01	0,65	2,84E-01	0,77	8,66E-01	1,17	1,02E-05	45,82	3,59E-06	1,00	6,46E-01	0,94
O75083	WDR1_HUMAN WD repeat-containing protein 1	23	22	8,03E-02	0,07	2,56E-01	0,86	5,21E-01	1,81	4,81E-04	141,35	4,81E-03	1,00	4,06E-01	0,94
P20339	RAB5A_HUMAN Ras-related protein Rab-5A	5	3	4,75E-01	0,75	3,48E-01	0,75	6,00E-01	1,30	1,00E+00	1,00	1,00E+00	0,50	8,13E-01	0,93
P50479	PDLI4_HUMAN PDZ and LIM domain protein 4	7	7	2,47E-01	0,20	2,27E-01	0,38	1,26E-01	2,86	4,55E-04	10,83	4,38E-07	1,00	7,10E-01	0,93
P23284	PPIB_HUMAN Peptidyl-prolyl cis-trans isomerase B	14	14	3,70E-01	0,26	9,81E-01	1,01	8,54E-01	0,73	2,83E-04	54,69	4,79E-04	1,00	7,73E-01	0,93
P22392	NDKB_HUMAN Nucleoside diphosphate kinase B	10	6	2,91E-01	0,19	2,96E-01	0,82	5,10E-01	2,52	3,53E-04	72,32	2,01E-03	1,00	5,78E-01	0,93
P49755	TMEDA_HUMAN Transmembrane emp24 domain-containing protein 10	11	11	1,72E-01	0,10	1,24E-01	0,58	9,70E-01	0,93	1,73E-04	106,27	1,21E-01	0,97	7,13E-01	0,93
O00560	SDCB1_HUMAN Syntenin-1	3	2	6,64E-01	0,67	8,16E-01	1,03	1,00E+00	1,00	3,50E-07	68,76	1,79E-02	0,99	8,15E-01	0,93

O15260	SURF4_HUMAN Surfeit locus protein 4	2	2	1,78E-01	0,05	1,94E-01	0,39	3,51E-01	1,91	2,40E-02	17,86	5,58E-02	0,99	8,79E-01	0,92
P12236	ADT3_HUMAN ADP/ATP translocase 3	8	4	2,88E-01	0,14	4,40E-01	0,69	1,00E+00	1,00	2,78E-07	179,08	1,62E-01	0,96	4,91E-01	0,92
P10155	RO60_HUMAN 60 kDa SS-A/Ro ribonucleoprotein	3	3	8,17E-01	0,69	7,55E-01	0,88	1,00E+00	1,00	5,98E-03	32,61	1,00E+00	0,50	8,44E-01	0,92
P51153	RAB13_HUMAN Ras-related protein Rab-13	3	2	8,99E-01	1,23	1,98E-01	0,30	7,59E-01	0,69	3,05E-02	8,80	1,52E-02	1,00	9,33E-01	0,92
P17301	ITA2_HUMAN Integrin alpha-2	19	19	2,83E-01	0,16	6,69E-03	0,53	7,88E-01	1,34	4,22E-01	1,57	2,43E-02	0,99	6,48E-01	0,92
O75390	CISY_HUMAN Citrate synthase, mitochondrial	10	10	4,70E-01	0,50	6,73E-01	0,88	5,55E-03	0,05	1,17E-02	24,83	1,77E-03	1,00	5,79E-01	0,92
Q53GQ0	DHB12_HUMAN Very-long-chain 3-oxoacyl-CoA reductase	4	4	2,83E-01	0,16	2,08E-01	0,47	1,00E+00	1,00	9,07E-05	57,89	1,20E-01	0,98	9,27E-01	0,92
P0DP25	CALM3_HUMAN Calmodulin-3	8	8	8,24E-02	0,28	9,64E-03	0,67	9,18E-02	17,14	1,92E-02	5,35	1,06E-04	1,00	6,56E-01	0,92
P68032	ACTC_HUMAN Actin, alpha cardiac muscle 1	29	1	2,52E-01	0,34	8,40E-02	1,25	1,28E-01	16,74	6,14E-04	10,45	8,37E-03	1,00	6,93E-01	0,92
Q8N257	H2B3B_HUMAN Histone H2B type 3-B	6	2	9,01E-01	1,25	2,57E-02	1,56	1,00E+00	1,00	1,67E-01	12,04	9,43E-05	1,00	7,37E-01	0,92
P36543	VATE1_HUMAN V-type proton ATPase subunit E 1	5	4	2,14E-01	0,16	1,35E-01	1,57	2,37E-01	7,45	7,10E-07	279,77	1,53E-01	0,97	9,52E-01	0,91
P05388	RLA0_HUMAN 60S acidic ribosomal protein P0	9	9	9,55E-02	0,15	9,62E-01	1,00	9,80E-01	0,97	3,10E-02	14,48	4,67E-03	1,00	6,12E-01	0,91
P48735	IDHP_HUMAN Isocitrate dehydrogenase [NADP], mitochondrial	11	10	6,63E-01	0,53	5,59E-01	1,29	9,65E-01	0,96	9,88E-05	148,44	2,05E-04	1,00	6,75E-01	0,91
P61158	ARP3_HUMAN Actin-related protein 3	20	19	1,86E-01	0,12	5,64E-01	0,89	7,56E-02	8,53	7,71E-05	120,29	1,69E-02	0,99	6,44E-01	0,91
P04839	CY24B_HUMAN Cytochrome b-245 heavy	5	5	4,59E-01	0,73	6,47E-01	1,49	1,00E+00	1,00	1,62E-05	75,43	1,68E-02	0,99	9,04E-01	0,91

	chain														
P14314	GLU2B_HUMAN Glucosidase 2 subunit beta	14	14	2,06E-01	0,08	1,62E-01	0,80	8,76E-01	1,17	1,70E-02	8,98	4,25E-04	1,00	5,94E-01	0,91
P35232	PHB_HUMAN Prohibitin	14	14	1,84E-01	0,09	3,99E-01	0,92	7,33E-01	1,29	1,29E-06	160,81	4,74E-04	1,00	5,62E-01	0,90
P13010	XRCC5_HUMAN X-ray repair cross-complementing protein 5	14	14	4,74E-02	0,10	6,96E-01	0,70	1,00E+00	1,00	9,98E-02	3,46	7,16E-02	0,99	8,16E-01	0,90
P18206	VINC_HUMAN Vinculin	47	47	3,53E-01	0,19	2,99E-01	0,91	2,29E-01	0,24	5,12E-03	6,75	1,37E-05	1,00	2,66E-01	0,90
P37837	TALDO_HUMAN Transaldolase	14	14	2,84E-01	0,28	6,98E-01	0,96	9,07E-02	12,65	2,79E-04	115,80	1,46E-04	1,00	1,98E-01	0,90
P04632	CPNS1_HUMAN Calpain small subunit 1	5	5	2,43E-01	0,14	2,99E-02	0,80	2,11E-01	3,11	1,55E-02	16,66	1,06E-01	0,98	6,64E-01	0,90
O75131	CPNE3_HUMAN Copine-3	6	6	6,47E-01	1,56	9,36E-01	1,11	7,34E-01	0,53	1,19E-02	12,53	9,59E-01	0,52	8,66E-01	0,90
P15559	NQO1_HUMAN NAD(P)H dehydrogenase [quinone] 1	9	9	2,76E-01	0,16	6,87E-01	0,93	9,22E-01	0,92	6,91E-03	11,40	9,98E-03	1,00	3,89E-01	0,90
P07954	FUMH_HUMAN Fumarate hydratase, mitochondrial	9	9	5,50E-01	0,35	1,54E-01	1,15	7,86E-01	0,76	1,06E-03	67,69	1,04E-05	1,00	5,33E-01	0,90
P06733	ENOA_HUMAN Alpha-enolase	34	32	1,83E-01	0,33	2,55E-01	0,85	5,72E-02	8,85	8,11E-04	13,12	1,52E-04	1,00	2,25E-02	0,90
Q16881	TRXR1_HUMAN Thioredoxin reductase 1, cytoplasmic	16	16	1,78E-01	0,09	2,95E-01	0,62	8,64E-01	1,12	1,12E-03	87,71	5,98E-04	1,00	8,47E-01	0,89
Q9UJU6	DBNL_HUMAN Drebrin-like protein	4	4	3,56E-02	0,07	7,78E-01	0,97	2,77E-01	0,25	3,76E-02	0,50	8,94E-03	1,00	3,73E-01	0,89
O95394	AGM1_HUMAN Phosphoacetylglucosamine mutase	7	7	3,54E-01	0,65	3,56E-02	0,33	1,00E+00	1,00	1,59E-01	3,07	3,70E-06	1,00	5,69E-01	0,89
P09601	HMOX1_HUMAN Heme oxygenase 1	2	2	1,00E+00	1,00	4,88E-01	1,76	1,00E+00	1,00	7,87E-03	40,97	1,00E+00	0,50	7,19E-01	0,89

O15143	ARC1B_HUMAN Actin-related protein 2/3 complex subunit 1B	8	8	2,51E-02	0,03	5,50E-02	1,35	7,18E-01	1,53	2,20E-08	206,14	2,44E-01	0,93	2,21E-01	0,89
Q9HDC9	APMAP_HUMAN Adipocyte plasma membrane-associated protein	10	10	6,15E-01	0,39	6,00E-01	1,11	5,02E-01	0,55	1,47E-03	104,43	6,71E-03	1,00	6,42E-01	0,89
O75396	SC22B_HUMAN Vesicle-trafficking protein SEC22b	10	10	2,37E-01	0,16	1,48E-01	0,79	5,25E-01	1,80	1,74E-03	110,61	6,05E-03	1,00	4,68E-01	0,89
P62917	RL8_HUMAN 60S ribosomal protein L8	6	6	3,29E-01	0,21	7,98E-01	1,04	1,00E+00	1,00	1,13E-02	8,99	1,95E-02	0,99	5,73E-01	0,89
P31153	METK2_HUMAN S-adenosylmethionine synthase isoform type-2	6	6	9,30E-01	1,19	2,72E-01	1,12	9,36E-02	4,48	1,30E-05	33,77	9,91E-03	1,00	4,19E-01	0,88
P13674	P4HA1_HUMAN Prolyl 4-hydroxylase subunit alpha-1	25	25	2,00E-01	0,10	1,39E-03	0,76	6,23E-01	0,57	5,44E-01	1,45	6,18E-04	1,00	2,16E-01	0,88
P02545	LMNA_HUMAN Prelamin-A/C	39	37	2,58E-01	0,25	8,58E-01	0,97	3,93E-01	0,66	5,57E-03	22,45	2,76E-03	1,00	2,56E-01	0,88
P04216	THY1_HUMAN Thy-1 membrane glycoprotein	4	4	8,84E-01	1,16	4,32E-02	0,66	4,98E-01	4,44	1,66E-01	3,61	7,96E-01	0,60	7,18E-01	0,88
P46777	RL5_HUMAN 60S ribosomal protein L5	15	15	5,78E-01	0,42	6,72E-01	0,88	5,84E-01	0,62	7,91E-03	7,32	9,90E-02	0,98	7,43E-01	0,88
P20073	ANXA7_HUMAN Annexin A7	8	8	3,46E-02	3,64	3,96E-01	0,43	5,23E-01	2,35	4,01E-01	4,04	1,18E-04	1,00	6,14E-01	0,87
Q9Y490	TLN1_HUMAN Talin-1	84	84	3,29E-01	0,25	1,46E-01	0,93	4,26E-01	2,19	2,55E-05	113,57	1,78E-05	1,00	1,43E-01	0,87
P54578	UBP14_HUMAN Ubiquitin carboxyl-terminal hydrolase 14	9	7	7,94E-01	1,37	1,22E-01	0,53	2,48E-01	0,29	4,15E-02	11,12	1,52E-04	1,00	5,99E-01	0,87
P52597	HNRPF_HUMAN Heterogeneous nuclear ribonucleoprotein F	5	2	1,94E-01	0,14	4,43E-01	1,37	7,90E-01	1,14	6,71E-03	9,24	4,93E-01	0,77	6,03E-01	0,86
Q9NR28	DBLOH_HUMAN Diablo homolog, mitochondrial	3	3	4,27E-01	0,31	9,88E-02	0,80	6,34E-01	0,50	1,06E-02	12,15	1,19E-01	0,98	4,55E-01	0,86

Q14974	IMB1_HUMAN Importin subunit beta-1	17	17	6,17E-01	0,47	1,72E-01	1,15	4,58E-04	39,95	3,42E-01	3,38	7,25E-02	0,98	3,34E-01	0,86
P18124	RL7_HUMAN 60S ribosomal protein L7	10	10	2,06E-01	0,06	5,03E-01	1,23	6,59E-01	0,66	1,00E-05	62,62	2,29E-01	0,93	6,04E-01	0,86
P61160	ARP2_HUMAN Actin-related protein 2	12	12	5,61E-02	0,04	6,95E-01	0,96	2,69E-01	4,42	9,84E-04	97,22	6,01E-03	1,00	3,51E-01	0,86
P04156	PRIO_HUMAN Major prion protein	3	3	3,91E-01	0,24	4,07E-04	0,56	1,00E+00	1,00	1,00E+00	1,00	1,91E-01	0,95	1,63E-01	0,86
P08758	ANXA5_HUMAN Annexin A5	27	27	1,38E-01	0,13	3,24E-03	0,70	3,27E-02	9,20	5,44E-03	26,37	3,30E-03	1,00	9,65E-02	0,86
Q8WUM4	PDC6I_HUMAN Programmed cell death 6-interacting protein	19	19	4,00E-01	0,34	4,01E-01	0,85	2,41E-01	3,98	5,97E-03	31,13	6,28E-04	1,00	6,45E-01	0,85
P20618	PSB1_HUMAN Proteasome subunit beta type-1	9	8	5,07E-01	0,39	2,75E-01	0,76	6,19E-01	0,71	2,39E-02	13,91	9,39E-03	1,00	5,32E-01	0,85
Q7KZF4	SND1_HUMAN Staphylococcal nuclease domain-containing protein 1	31	31	1,23E-01	0,06	9,61E-01	1,01	2,00E-01	0,41	6,39E-03	17,20	1,31E-03	1,00	5,03E-01	0,85
Q99880	H2B1L_HUMAN Histone H2B type 1-L	7	3	9,42E-02	0,22	5,17E-01	1,21	8,04E-01	1,52	2,23E-02	34,20	1,77E-03	1,00	6,23E-01	0,85
P18085	ARF4_HUMAN ADP-ribosylation factor 4	8	4	1,87E-01	0,12	3,41E-01	1,08	6,61E-02	6,03	2,32E-05	27,28	5,53E-04	1,00	1,51E-01	0,85
Q9BS40	LXN_HUMAN Latexin	2	2	1,77E-01	0,26	5,65E-01	0,82	1,00E+00	1,00	2,28E-02	13,03	5,17E-02	0,99	4,86E-01	0,85
Q9NQC3	RTN4_HUMAN Reticulon-4	9	9	3,54E-01	0,16	5,91E-02	0,45	2,52E-01	4,72	9,67E-04	100,73	4,44E-04	1,00	6,14E-01	0,84
P08962	CD63_HUMAN CD63 antigen	2	2	3,71E-01	0,26	3,58E-02	1,94	1,00E+00	1,00	1,36E-06	186,74	1,68E-01	0,96	8,75E-01	0,84
P14927	QCR7_HUMAN Cytochrome b-c1 complex subunit 7	3	3	1,26E-01	0,37	4,17E-01	2,29	7,57E-02	2,58	3,99E-02	3,09	1,66E-01	0,96	6,42E-01	0,83
P10412	H14_HUMAN Histone H1.4	9	3	4,73E-01	0,25	1,98E-01	1,36	7,60E-02	19,26	4,59E-04	7,48	3,76E-05	1,00	6,96E-01	0,83

Q99832	TCPH_HUMAN T-complex protein 1 subunit eta	16	15	4,30E-01	0,24	6,19E-01	0,86	7,16E-01	1,20	6,01E-05	47,55	2,71E-04	1,00	5,25E-01	0,83
P04083	ANXA1_HUMAN Annexin A1	23	23	1,08E-01	0,22	6,15E-03	0,80	1,30E-01	12,04	6,53E-03	34,26	1,44E-03	1,00	3,86E-02	0,83
P31939	PUR9_HUMAN Bifunctional purine biosynthesis protein PURH	14	14	1,92E-02	0,12	2,66E-01	0,45	6,44E-01	1,44	8,20E-01	0,81	3,96E-01	0,83	5,08E-01	0,82
Q71DI3	H32_HUMAN Histone H3.2	3	3	2,53E-01	0,21	8,56E-01	1,13	5,87E-01	0,48	1,24E-02	25,27	3,75E-02	0,99	5,94E-01	0,82
Q8N2K0	ABD12_HUMAN Lysophosphatidylserine lipase ABHD12	4	4	1,00E+00	1,00	3,49E-01	2,66	1,00E+00	1,00	2,35E-02	24,01	4,12E-03	1,00	5,29E-01	0,82
Q15436	SC23A_HUMAN Protein transport protein Sec23A	13	13	8,07E-03	0,02	6,65E-01	0,87	1,00E+00	1,00	8,05E-02	2,47	3,88E-02	0,99	2,90E-01	0,82
P16401	H15_HUMAN Histone H1.5	6	6	2,42E-01	0,12	2,31E-01	1,42	6,65E-01	2,08	1,87E-06	78,56	1,71E-04	1,00	5,98E-01	0,82
Q9H0U4	RAB1B_HUMAN Ras-related protein Rab-1B	9	3	6,89E-01	0,60	5,28E-01	0,55	1,00E+00	1,00	1,61E-04	11,34	1,47E-01	0,97	6,45E-01	0,81
Q9Y678	COPG1_HUMAN Coatomer subunit gamma-1	18	18	5,89E-02	0,03	2,43E-01	0,85	2,33E-01	2,18	3,45E-02	3,10	7,99E-03	1,00	5,26E-01	0,81
Q10567	AP1B1_HUMAN AP-1 complex subunit beta-1	10	3	1,37E-01	0,24	6,77E-01	0,86	1,00E+00	1,00	2,08E-05	31,73	8,07E-03	1,00	3,93E-01	0,81
P16152	CBR1_HUMAN Carbonyl reductase [NADPH] 1	10	10	6,31E-01	0,43	7,94E-02	0,72	8,69E-01	0,81	4,88E-05	54,10	1,02E-02	1,00	1,52E-01	0,81
Q16401	PSMD5_HUMAN 26S proteasome non-ATPase regulatory subunit 5	6	6	3,67E-01	0,56	7,89E-01	1,39	1,00E+00	1,00	4,85E-02	2,14	3,45E-02	0,99	5,44E-01	0,80
P49411	EFTU_HUMAN Elongation factor Tu, mitochondrial	11	11	2,23E-01	0,14	3,25E-01	0,77	8,00E-01	0,82	9,40E-07	143,19	1,09E-01	0,98	5,55E-01	0,80
Q7Z2W4	ZCCHV_HUMAN Zinc finger CCCH-type antiviral protein 1	4	4	9,67E-01	1,05	5,97E-01	0,80	5,77E-01	0,51	3,34E-02	8,07	4,98E-02	0,99	8,36E-01	0,80
O43684	BUB3_HUMAN Mitotic	4	4	3,47E-	0,18	9,26E-	0,95	3,96E-	0,47	8,18E-	1,19	2,87E-	0,99	8,03E-	0,79

	checkpoint protein BUB3			02		01		01		01		02		01	
P60228	EIF3E_HUMAN Eukaryotic translation initiation factor 3 subunit E	7	7	5,03E-01	0,44	2,98E-01	0,77	1,00E+00	1,00	1,72E-02	6,24	6,29E-03	1,00	3,26E-01	0,79
Q13636	RAB31_HUMAN Ras-related protein Rab-31	2	2	5,81E-01	0,81	6,84E-03	0,45	1,00E+00	1,00	4,30E-02	2,61	1,88E-02	0,99	5,51E-01	0,79
Q16658	FSCN1_HUMAN Fascin	21	21	1,34E-01	0,09	3,19E-01	0,89	2,96E-02	2,77	1,37E-01	2,16	3,28E-03	1,00	4,83E-01	0,79
P07910	HNRPC_HUMAN Heterogeneous nuclear ribonucleoproteins C1/C2	7	7	1,40E-01	0,08	6,38E-03	1,83	3,91E-01	0,40	1,89E-02	27,70	3,19E-02	0,99	6,47E-01	0,79
Q9UN86	G3BP2_HUMAN Ras GTPase-activating protein-binding protein 2	2	2	2,50E-02	0,07	2,17E-04	0,07	8,37E-01	0,89	1,00E+00	1,00	6,50E-01	0,67	8,13E-01	0,79
P53367	ARFP1_HUMAN Arfaptin-1	4	4	4,39E-02	3,35	3,16E-01	0,75	3,80E-01	1,55	5,41E-01	1,18	4,80E-01	0,78	8,81E-02	0,79
P04792	HSPB1_HUMAN Heat shock protein beta-1	13	13	1,39E-01	0,09	1,33E-01	0,88	4,80E-02	13,71	6,04E-04	53,83	6,18E-03	1,00	6,82E-02	0,79
Q05655	KPCD_HUMAN Protein kinase C delta type	2	2	1,00E+00	1,00	1,00E+00	1,00	1,00E+00	1,00	2,22E-05	62,07	1,00E+00	0,50	6,22E-01	0,78
P51659	DHB4_HUMAN Peroxisomal multifunctional enzyme type 2	17	17	3,63E-02	14,78	5,71E-01	1,13	7,59E-01	0,56	2,87E-04	154,27	6,13E-01	0,69	2,89E-01	0,78
P16615	AT2A2_HUMAN Sarcoplasmic/endoplasmic reticulum calcium ATPase 2	15	12	3,82E-01	0,23	9,48E-01	1,02	4,49E-01	1,93	2,57E-04	80,07	2,94E-02	0,99	3,53E-01	0,78
Q9NR31	SAR1A_HUMAN GTP-binding protein SAR1a	3	3	6,88E-01	0,51	2,44E-01	1,29	1,00E+00	1,00	4,65E-05	37,58	7,13E-02	0,99	7,80E-01	0,77
P13284	GILT_HUMAN Gamma-interferon-inducible lysosomal thiol reductase	3	3	4,81E-01	0,63	9,92E-01	0,99	2,65E-01	2,13	1,52E-02	42,55	6,42E-02	0,99	2,03E-01	0,77
Q15019	SEPT2_HUMAN Septin-2	11	11	1,42E-01	0,29	2,90E-01	0,78	1,45E-02	19,57	2,35E-01	1,81	1,09E-01	0,98	1,38E-01	0,77

P08648	ITA5_HUMAN Integrin alpha-5	11	11	4,72E-01	0,33	3,81E-01	0,83	4,00E-02	24,34	1,03E-01	3,62	1,56E-02	1,00	3,14E-01	0,77
P27824	CALX_HUMAN Calnexin	22	22	5,72E-01	0,51	4,61E-01	0,71	3,32E-01	4,77	2,57E-04	229,57	9,22E-02	0,98	3,37E-01	0,77
Q09666	AHNK_HUMAN Neuroblast differentiation-associated protein AHNAK	194	193	2,56E-01	0,13	7,30E-03	0,70	6,68E-01	0,62	3,41E-05	79,64	2,28E-03	1,00	1,81E-01	0,77
P36578	RL4_HUMAN 60S ribosomal protein L4	15	14	1,28E-01	0,10	4,24E-02	0,79	7,23E-01	0,77	5,76E-05	40,70	2,66E-02	0,99	5,26E-02	0,77
P17900	SAP3_HUMAN Ganglioside GM2 activator	3	3	3,09E-01	0,66	9,34E-01	0,95	1,00E+00	1,00	7,43E-05	105,52	1,00E+00	0,50	2,40E-01	0,76
P60953	CDC42_HUMAN Cell division control protein 42 homolog	8	7	1,03E-01	0,09	1,28E-01	0,76	1,39E-01	14,02	9,00E-03	54,08	6,18E-02	0,99	3,91E-01	0,76
O60763	USO1_HUMAN General vesicular transport factor p115	21	21	3,68E-01	2,81	9,25E-01	0,98	5,37E-01	0,46	3,47E-04	17,97	2,23E-03	1,00	1,28E-01	0,76
P50395	GDIB_HUMAN Rab GDP dissociation inhibitor beta	24	16	2,71E-01	0,35	1,04E-01	0,81	9,42E-01	0,91	5,74E-03	71,51	1,42E-02	1,00	1,39E-01	0,75
P30740	ILEU_HUMAN Leukocyte elastase inhibitor	8	7	7,87E-02	0,22	3,31E-01	0,38	3,56E-01	0,47	1,14E-04	104,76	1,89E-02	0,99	5,57E-01	0,75
P0DMV9	HS71B_HUMAN Heat shock 70 kDa protein 1B	17	9	7,71E-01	0,56	4,25E-01	1,18	5,46E-01	0,47	9,54E-03	33,47	7,70E-03	1,00	3,70E-01	0,75
Q86UX7	URP2_HUMAN Fermitin family homolog 3	12	12	9,40E-01	0,95	1,68E-01	2,58	4,15E-01	0,46	2,29E-05	322,24	8,79E-03	1,00	7,67E-01	0,75
P50914	RL14_HUMAN 60S ribosomal protein L14	4	4	2,62E-01	0,12	7,29E-01	0,92	7,33E-01	0,64	2,41E-05	84,56	2,00E-03	1,00	3,73E-01	0,74
P14550	AK1A1_HUMAN Aldo-keto reductase family 1 member A1	10	10	1,73E-01	0,10	9,18E-01	0,95	1,79E-01	0,19	3,28E-06	177,45	9,72E-01	0,51	2,35E-01	0,74
P18669	PGAM1_HUMAN Phosphoglycerate mutase 1	19	19	1,04E-01	0,12	4,98E-01	0,90	5,97E-01	2,32	2,12E-06	288,64	1,28E-03	1,00	4,01E-02	0,74
P19367	HXK1_HUMAN Hexokinase-1	16	15	4,51E-01	0,29	7,19E-02	0,62	7,60E-01	1,36	6,19E-03	17,36	2,37E-02	0,99	2,66E-01	0,73

P31949	S10AB_HUMAN Protein S100-A11	5	5	4,87E-02	0,26	3,44E-01	0,78	3,78E-02	49,68	9,15E-03	79,64	2,18E-02	0,99	1,53E-01	0,73
P11940	PABP1_HUMAN Polyadenylate-binding protein 1	23	14	9,31E-01	0,88	6,25E-01	1,11	9,83E-01	0,98	2,56E-03	28,48	5,21E-02	0,99	1,43E-01	0,73
P62244	RS15A_HUMAN 40S ribosomal protein S15a	5	5	7,32E-01	1,27	5,42E-02	0,82	4,12E-01	0,26	6,70E-02	8,26	3,97E-04	1,00	6,73E-01	0,73
O60684	IMA7_HUMAN Importin subunit alpha-7	4	3	4,20E-01	0,60	4,48E-02	0,13	1,00E+00	1,00	1,39E-01	1,87	1,00E+00	0,50	5,50E-01	0,73
P22234	PUR6_HUMAN Multifunctional protein ADE2	13	13	5,60E-01	0,44	7,59E-01	1,13	8,95E-01	1,07	3,11E-04	6,96	1,40E-01	0,97	5,08E-01	0,72
P20700	LMNB1_HUMAN Lamin-B1	11	8	2,46E-01	0,22	7,97E-01	0,89	4,76E-01	0,46	4,92E-03	20,87	2,60E-01	0,92	5,34E-01	0,72
P46821	MAP1B_HUMAN Microtubule-associated protein 1B	50	49	1,18E-01	0,17	8,56E-02	0,52	1,94E-02	0,10	9,93E-02	0,17	4,88E-04	1,00	3,18E-01	0,72
Q14203	DCTN1_HUMAN Dynactin subunit 1	12	12	5,92E-01	0,60	1,88E-01	0,66	3,89E-01	0,44	3,07E-03	13,88	4,14E-06	1,00	2,41E-01	0,72
Q68CZ2	TENS3_HUMAN Tensin-3	3	3	3,76E-01	0,37	3,45E-01	0,65	7,37E-01	0,74	1,46E-02	5,85	7,55E-01	0,62	6,60E-01	0,72
P62913	RL11_HUMAN 60S ribosomal protein L11	8	7	3,30E-01	0,17	1,93E-01	1,25	1,99E-02	36,49	1,58E-06	241,23	3,26E-01	0,88	2,54E-02	0,72
P08670	VIME_HUMAN Vimentin	70	64	1,10E-01	0,18	6,71E-01	1,07	9,46E-02	6,74	6,44E-03	63,08	1,99E-03	1,00	2,26E-01	0,72
P51572	BAP31_HUMAN B-cell receptor-associated protein 31	10	10	5,13E-01	0,38	7,88E-01	0,96	7,58E-01	0,74	2,62E-06	149,28	1,42E-01	0,97	1,84E-01	0,71
P46060	RAGP1_HUMAN Ran GTPase-activating protein 1	6	6	9,13E-01	0,85	7,65E-01	0,90	2,31E-01	0,20	5,21E-01	1,61	2,37E-02	0,99	7,74E-01	0,71
P35606	COPB2_HUMAN Coatomer subunit beta	15	15	5,40E-01	2,37	5,89E-01	0,90	1,18E-02	3,19	2,75E-01	0,56	7,75E-03	1,00	2,11E-01	0,71
Q9HD20	AT131_HUMAN Manganese-transporting ATPase 13A1	4	4	1,30E-01	0,33	8,48E-02	0,31	6,86E-01	0,71	9,48E-03	8,40	1,46E-02	1,00	1,91E-01	0,71

Q01650	LAT1_HUMAN Large neutral amino acids transporter small subunit 1	3	3	3,74E-02	0,05	8,45E-01	1,04	1,00E+00	1,00	1,00E+00	1,00	1,17E-02	1,00	1,55E-02	0,71
Q9NZ01	TECR_HUMAN Very-long-chain enoyl-CoA reductase	2	2	2,03E-01	0,51	2,68E-01	0,47	1,00E+00	1,00	3,84E-02	8,90	1,00E+00	0,50	8,15E-02	0,71
P83111	LACTB_HUMAN Serine beta-lactamase-like protein LACTB, mitochondrial	8	8	5,63E-01	0,78	2,16E-01	0,35	2,35E-01	1,61	8,18E-06	46,79	4,42E-02	0,99	6,38E-01	0,71
P30101	PDIA3_HUMAN Protein disulfide-isomerase A3	36	36	2,87E-01	0,45	5,12E-01	1,12	2,73E-01	1,52	5,00E-03	6,02	5,26E-02	0,99	4,20E-01	0,71
P10619	PPGB_HUMAN Lysosomal protective protein	3	3	3,41E-01	0,15	2,48E-01	1,22	6,10E-01	2,12	1,47E-03	50,20	5,92E-01	0,70	7,58E-01	0,71
P52209	6PGD_HUMAN 6-phosphogluconate dehydrogenase, decarboxylating	17	16	3,59E-01	0,20	3,62E-01	0,67	5,24E-01	3,47	3,10E-04	45,40	8,35E-02	0,98	1,89E-02	0,71
P52272	HNRPM_HUMAN Heterogeneous nuclear ribonucleoprotein M	18	18	1,18E-01	0,26	5,30E-01	1,09	2,96E-01	4,28	2,19E-03	46,25	1,32E-02	1,00	2,60E-01	0,70
P36776	LONM_HUMAN Lon protease homolog, mitochondrial	14	13	4,34E-01	2,61	4,21E-01	0,51	6,16E-01	0,60	1,29E-04	23,29	1,50E-02	1,00	5,25E-01	0,70
P12268	IMDH2_HUMAN Inosine-5-monophosphate dehydrogenase 2	7	7	2,23E-01	0,25	9,86E-01	0,99	5,71E-02	21,07	1,21E-02	21,99	2,78E-02	0,99	2,77E-01	0,70
Q9Y2J2	E41L3_HUMAN Band 4.1-like protein 3	5	5	2,52E-01	0,47	3,30E-01	1,68	1,00E+00	1,00	1,06E-02	21,06	1,00E+00	0,50	6,57E-01	0,70
Q06210	GFPT1_HUMAN Glutamine--fructose-6-phosphate aminotransferase [isomerizing] 1	13	11	2,07E-01	0,15	2,55E-02	0,55	3,71E-01	2,56	2,39E-01	0,31	2,78E-02	0,99	6,69E-02	0,70
Q562R1	ACTBL_HUMAN Beta-actin-like protein 2	10	3	3,18E-02	0,04	6,64E-01	1,52	6,22E-01	2,27	6,09E-06	88,47	2,05E-02	0,99	8,02E-01	0,70

Q16543	CDC37_HUMAN Hsp90 co-chaperone Cdc37	9	9	2,80E-01	0,22	8,60E-02	0,64	7,87E-02	5,32	5,53E-05	22,44	1,67E-02	0,99	3,06E-01	0,70
Q9UGL1	KDM5B_HUMAN Lysine-specific demethylase 5B	3	2	5,25E-01	0,38	6,35E-01	0,62	6,51E-01	0,71	1,05E-04	30,20	1,00E+00	0,50	5,18E-01	0,70
O43795	MYO1B_HUMAN Unconventional myosin-Ib	3	3	6,50E-02	9,70	1,42E-01	1,28	5,93E-01	0,38	2,17E-02	34,06	9,26E-01	0,53	8,11E-01	0,70
P12931	SRC_HUMAN Proto-oncogene tyrosine-protein kinase Src	2	2	1,00E+00	1,00	9,01E-01	1,03	1,00E+00	1,00	4,40E-02	3,50	6,08E-03	1,00	1,26E-01	0,70
P28838	AMPL_HUMAN Cytosol aminopeptidase	11	11	3,57E-01	0,35	4,52E-01	0,82	6,44E-01	0,55	8,13E-03	15,70	6,63E-01	0,66	2,98E-01	0,69
P04040	CATA_HUMAN Catalase	11	10	3,38E-01	2,73	2,82E-01	1,59	4,66E-01	0,35	4,23E-02	14,51	7,38E-03	1,00	3,32E-01	0,69
P04843	RPN1_HUMAN Dolichyl-diphosphooligosaccharide--protein glycosyltransferase subunit 1	22	22	2,95E-01	0,25	8,78E-01	1,02	8,87E-01	1,30	8,58E-04	3,50	2,11E-03	1,00	1,83E-01	0,69
P52907	CAZA1_HUMAN F-actin-capping protein subunit alpha-1	12	9	2,13E-01	0,11	6,20E-01	1,10	1,00E+00	1,00	8,02E-07	138,75	3,35E-05	1,00	1,39E-01	0,69
Q99878	H2A1J_HUMAN Histone H2A type 1-J	6	0	2,37E-02	0,25	7,89E-02	1,63	4,09E-02	94,25	1,19E-03	233,04	6,76E-01	0,66	3,18E-02	0,69
Q9ULV4	COR1C_HUMAN Coronin-1C	8	8	1,67E-01	0,07	1,79E-01	0,66	7,07E-01	0,68	2,90E-02	22,52	1,20E-01	0,97	2,61E-01	0,69
Q99536	VAT1_HUMAN Synaptic vesicle membrane protein VAT-1 homolog	14	14	2,00E-01	0,18	5,57E-02	0,66	2,65E-01	6,03	2,78E-04	211,45	3,62E-04	1,00	6,39E-02	0,68
P18084	ITB5_HUMAN Integrin beta-5	6	5	2,99E-01	0,37	3,59E-02	0,40	8,13E-01	1,23	1,24E-03	23,18	3,72E-02	0,99	7,17E-01	0,68
P09467	F16P1_HUMAN Fructose-1,6-bisphosphatase 1	12	11	5,95E-01	0,55	5,47E-01	1,72	7,93E-02	45,41	9,36E-06	823,59	8,67E-01	0,56	5,71E-01	0,68
P02751	FINC_HUMAN Fibronectin	102	102	3,04E-02	0,06	8,04E-01	1,15	2,60E-01	0,20	2,72E-01	2,69	2,02E-04	1,00	5,33E-01	0,68

P22314	UBA1_HUMAN Ubiquitin-like modifier-activating enzyme 1	25	25	2,96E-01	0,11	4,31E-01	1,08	8,88E-01	0,88	7,61E-04	73,04	7,79E-03	1,00	2,34E-01	0,68
P00403	COX2_HUMAN Cytochrome c oxidase subunit 2	2	2	1,09E-01	0,14	5,69E-01	0,52	1,00E+00	1,00	1,00E-06	99,81	5,35E-02	0,99	4,80E-01	0,68
Q00610	CLH1_HUMAN Clathrin heavy chain 1	48	48	2,04E-02	0,30	4,51E-01	0,88	5,35E-01	0,33	1,86E-04	21,36	1,81E-03	1,00	2,14E-02	0,67
P46778	RL21_HUMAN 60S ribosomal protein L21	3	3	3,21E-01	0,22	8,07E-01	1,04	8,02E-01	0,81	1,15E-02	14,62	3,18E-02	0,99	1,82E-01	0,67
O14558	HSPB6_HUMAN Heat shock protein beta-6	4	4	1,68E-01	0,07	8,09E-01	0,94	8,66E-01	0,88	1,73E-06	72,53	5,52E-01	0,73	1,36E-01	0,67
Q15637	SF01_HUMAN Splicing factor 1	2	2	5,49E-03	0,08	9,23E-01	0,91	6,79E-01	0,65	5,56E-01	2,40	5,57E-02	0,99	8,11E-01	0,67
Q15293	RCN1_HUMAN Reticulocalbin-1	9	9	2,98E-02	0,05	5,57E-01	0,77	6,60E-01	0,67	4,65E-01	0,54	4,87E-05	1,00	4,70E-01	0,67
O94973	AP2A2_HUMAN AP-2 complex subunit alpha-2	7	4	1,21E-01	0,40	2,35E-01	0,41	1,00E+00	1,00	9,87E-03	3,68	6,98E-02	0,99	1,40E-01	0,67
P13797	PLST_HUMAN Plastin-3	30	21	3,53E-02	0,02	7,70E-02	0,73	3,50E-01	0,59	9,47E-01	1,04	2,63E-04	1,00	1,35E-01	0,66
P42765	THIM_HUMAN 3-ketoacyl-CoA thiolase, mitochondrial	6	6	1,71E-01	0,33	2,93E-01	0,45	8,48E-01	1,14	7,04E-06	86,83	4,31E-03	1,00	6,36E-01	0,66
P28070	PSB4_HUMAN Proteasome subunit beta type-4	7	7	9,37E-01	0,88	2,36E-03	0,42	7,23E-01	0,60	3,83E-01	2,66	1,89E-01	0,95	2,36E-01	0,66
P15153	RAC2_HUMAN Ras-related C3 botulinum toxin substrate 2	6	1	1,00E+00	1,00	6,05E-01	0,75	1,00E+00	1,00	3,96E-06	241,03	1,00E+00	0,50	3,41E-01	0,66
P06744	G6PI_HUMAN Glucose-6-phosphate isomerase	13	9	1,73E-01	0,11	7,90E-01	0,96	2,96E-01	5,20	3,67E-06	163,96	3,05E-04	1,00	6,04E-02	0,66
P61353	RL27_HUMAN 60S ribosomal protein L27	7	7	9,21E-01	1,20	7,60E-01	1,07	9,64E-01	0,96	2,41E-01	6,35	7,92E-02	0,98	4,81E-02	0,66
P04066	FUCO_HUMAN Tissue alpha-L-fucosidase	7	7	9,80E-01	1,03	2,31E-01	1,90	3,03E-01	2,29	1,19E-02	141,59	8,78E-02	0,98	4,44E-01	0,65
Q16795	NDUA9_HUMAN NADH	2	2	3,73E-	0,13	5,02E-	1,90	1,20E-	3,36	2,14E-	3,49	7,62E-	0,98	6,33E-	0,65

	dehydrogenase [ubiquinone] 1 alpha subcomplex subunit 9, mitochondrial			02		01		01		01		02		01	
Q15836	VAMP3_HUMAN Vesicle-associated membrane protein 3	5	1	5,89E-01	0,36	7,97E-02	0,81	5,16E-01	1,38	1,04E-06	163,11	5,82E-03	1,00	1,56E-01	0,65
P10301	RRAS_HUMAN Ras-related protein R-Ras	2	2	1,25E-01	0,18	3,49E-02	0,20	1,00E+00	1,00	1,00E+00	1,00	1,34E-01	0,97	4,99E-01	0,64
O95336	6PGL_HUMAN 6-phosphogluconolactonase	8	8	6,76E-01	0,51	1,87E-02	0,62	2,92E-01	0,26	1,44E-02	19,26	2,49E-01	0,93	1,01E-01	0,64
P40121	CAPG_HUMAN Macrophage-capping protein	14	14	1,70E-01	7,00	1,38E-01	0,71	2,70E-01	6,75	8,31E-03	140,92	3,67E-02	0,99	1,28E-01	0,64
P00558	PGK1_HUMAN Phosphoglycerate kinase 1	24	24	2,67E-01	0,12	4,39E-01	0,87	5,66E-01	2,11	7,10E-07	105,98	1,07E-04	1,00	1,06E-02	0,64
Q15006	EMC2_HUMAN ER membrane protein complex subunit 2	7	6	1,53E-01	0,15	4,80E-01	1,40	5,68E-01	0,46	7,87E-04	15,15	2,85E-01	0,91	3,59E-01	0,64
Q14697	GANAB_HUMAN Neutral alpha-glucosidase AB	31	31	3,14E-01	0,44	4,31E-01	0,94	4,21E-01	2,44	7,21E-04	28,71	1,92E-01	0,95	2,36E-02	0,64
Q13813	SPTN1_HUMAN Spectrin alpha chain, non-erythrocytic 1	62	61	1,98E-01	0,24	4,42E-01	0,81	9,88E-01	0,98	3,11E-01	2,33	2,51E-01	0,92	4,04E-02	0,64
Q13404	UB2V1_HUMAN Ubiquitin-conjugating enzyme E2 variant 1	7	2	1,31E-01	0,12	1,11E-01	0,44	9,10E-01	1,11	2,71E-02	5,35	2,25E-02	0,99	2,32E-01	0,64
Q96AY3	FKB10_HUMAN Peptidyl-prolyl cis-trans isomerase FKBP10	21	21	5,83E-02	0,06	2,37E-02	0,52	5,82E-01	1,69	8,51E-01	0,82	4,07E-03	1,00	1,02E-01	0,64
Q92820	GGH_HUMAN Gamma-glutamyl hydrolase	5	5	3,79E-03	0,05	1,06E-01	1,26	9,53E-01	0,95	3,88E-02	1,75	2,29E-01	0,94	1,27E-01	0,64
O75874	IDHC_HUMAN Isocitrate dehydrogenase [NADP] cytoplasmic	14	13	1,05E-01	0,07	1,86E-01	0,74	1,14E-01	4,56	6,26E-06	272,86	9,01E-05	1,00	8,40E-02	0,63

Q9H3U1	UN45A_HUMAN Protein unc-45 homolog A	3	3	1,54E-01	0,45	5,69E-01	0,66	6,79E-01	0,65	1,00E+00	1,00	1,00E+00	0,50	6,68E-01	0,63
O60749	SNX2_HUMAN Sorting nexin-2	8	5	5,15E-01	0,41	2,80E-01	0,77	8,61E-01	1,20	1,11E-06	91,43	1,30E-02	1,00	1,77E-01	0,63
P04439	HLAA_HUMAN HLA class I histocompatibility antigen, A alpha chain	9	5	3,61E-01	0,24	4,60E-01	0,58	3,78E-01	2,76	9,52E-04	155,49	1,44E-03	1,00	1,08E-01	0,63
Q00839	HNRPU_HUMAN Heterogeneous nuclear ribonucleoprotein U	13	13	3,52E-01	0,25	5,25E-01	1,10	8,06E-01	1,15	1,26E-06	95,01	6,95E-06	1,00	4,23E-02	0,63
P62873	GBB1_HUMAN Guanine nucleotide-binding protein G(I)/G(S)/G(T) subunit beta-1	6	6	3,27E-01	0,19	3,89E-01	0,65	1,00E+00	1,00	3,54E-04	57,84	1,66E-02	0,99	3,22E-01	0,62
Q9P0J0	NDUAD_HUMAN NADH dehydrogenase [ubiquinone] 1 alpha subcomplex subunit 13	2	2	2,89E-01	0,37	4,53E-02	0,19	1,00E+00	1,00	1,00E+00	1,00	1,00E+00	0,50	6,33E-01	0,62
P05387	RLA2_HUMAN 60S acidic ribosomal protein P2	6	6	5,19E-01	0,24	9,49E-01	0,98	2,54E-01	2,46	2,56E-02	5,70	7,26E-02	0,98	5,43E-01	0,62
P30041	PRDX6_HUMAN Peroxiredoxin-6	18	18	1,46E-01	0,12	2,50E-02	0,75	8,63E-01	1,12	2,31E-04	5,94	3,26E-05	1,00	8,69E-02	0,62
P07339	CATD_HUMAN Cathepsin D	17	16	8,86E-01	0,81	4,30E-02	0,58	1,64E-01	6,60	3,15E-03	21,22	4,73E-01	0,78	1,08E-01	0,62
P08575	PTPRC_HUMAN Receptor-type tyrosine-protein phosphatase C	14	14	7,69E-01	0,77	3,42E-01	0,46	7,45E-01	1,54	2,56E-05	30,32	8,56E-01	0,57	4,26E-01	0,61
Q9H4G4	GAPR1_HUMAN Golgi-associated plant pathogenesis-related protein 1	3	3	8,38E-01	0,80	6,08E-01	1,08	1,73E-01	3,31	4,76E-05	62,80	7,14E-03	1,00	1,92E-01	0,61
Q9UBR2	CATZ_HUMAN Cathepsin Z	6	6	1,21E-01	2,98	1,83E-01	4,97	5,57E-01	0,46	1,42E-03	97,31	5,82E-06	1,00	3,91E-02	0,61
Q15257	PTPA_HUMAN Serine/threonine-protein phosphatase 2A activator	3	3	1,28E-01	0,23	5,69E-01	0,84	1,00E+00	1,00	4,68E-02	1,87	1,00E+00	0,50	4,57E-02	0,61

Q15149	PLEC_HUMAN Plectin	181	179	1,49E-01	0,05	7,35E-02	0,72	7,51E-01	1,26	6,13E-04	153,39	1,78E-04	1,00	1,70E-02	0,61
P21926	CD9_HUMAN CD9 antigen	3	3	5,53E-01	1,84	2,22E-01	0,27	7,21E-01	0,50	3,67E-02	14,36	4,37E-01	0,80	3,67E-01	0,61
Q9Y3B3	TMED7_HUMAN Transmembrane emp24 domain-containing protein 7	5	5	3,39E-01	0,35	7,19E-01	0,91	5,79E-01	0,71	4,31E-02	5,48	3,59E-02	0,99	3,12E-01	0,60
P50213	IDH3A_HUMAN Isocitrate dehydrogenase [NAD] subunit alpha, mitochondrial	4	4	9,27E-01	1,12	2,05E-01	0,37	1,00E+00	1,00	6,46E-06	44,23	1,11E-01	0,98	5,60E-01	0,60
O15145	ARPC3_HUMAN Actin-related protein 2/3 complex subunit 3	6	6	4,57E-01	0,27	4,89E-01	0,79	1,00E+00	1,00	1,53E-01	1,66	6,44E-02	0,99	2,79E-01	0,60
P54819	KAD2_HUMAN Adenylate kinase 2, mitochondrial	3	3	1,11E-01	0,10	2,28E-01	0,73	9,27E-01	1,05	5,36E-03	10,75	4,03E-01	0,83	7,14E-01	0,60
Q08431	MFGM_HUMAN Lactadherin	4	4	9,23E-01	1,15	1,15E-02	0,12	6,53E-01	0,57	5,80E-01	0,45	8,11E-02	0,98	5,79E-01	0,59
Q02543	RL18A_HUMAN 60S ribosomal protein L18a	2	2	1,98E-01	0,38	3,07E-03	2,23	6,28E-01	1,25	6,46E-02	1,73	6,90E-02	0,99	1,19E-01	0,59
P61088	UBE2N_HUMAN Ubiquitin-conjugating enzyme E2 N	7	7	4,39E-01	0,38	1,24E-01	0,47	6,70E-01	1,72	2,73E-02	5,75	1,17E-02	1,00	3,86E-01	0,59
Q9UHX1	PUF60_HUMAN Poly(U)-binding-splicing factor PUF60	3	3	6,77E-02	0,46	7,94E-01	1,28	1,00E+00	1,00	1,00E+00	1,00	1,00E+00	0,50	2,08E-01	0,59
P08123	CO1A2_HUMAN Collagen alpha-2(I) chain	36	36	1,45E-01	0,08	6,54E-01	0,94	8,53E-01	0,86	1,22E-01	4,22	1,41E-03	1,00	6,11E-02	0,59
O15498	YKT6_HUMAN Synaptobrevin homolog YKT6	3	3	2,61E-01	0,40	5,10E-01	0,76	8,01E-01	0,78	6,62E-01	1,50	9,90E-02	0,98	4,27E-02	0,59
Q92783	STAM1_HUMAN Signal transducing adapter molecule 1	3	2	1,06E-01	0,02	7,27E-01	1,17	7,30E-01	3,02	7,31E-01	1,12	4,10E-02	0,99	7,08E-01	0,59
Q13576	IQGA2_HUMAN Ras GTPase-activating-like protein	11	10	2,05E-01	1,87	8,46E-01	1,10	4,61E-01	1,60	1,04E-03	14,76	3,18E-01	0,88	1,81E-01	0,58

	IQGAP2															
Q9NVA2	SEP11_HUMAN Septin-11	11	9	7,72E-02	0,07	3,03E-01	0,74	7,36E-01	0,50	6,72E-01	0,59	5,77E-02	0,99	4,93E-02	0,58
P23141	EST1_HUMAN Liver carboxylesterase 1	7	7	5,39E-01	0,53	5,70E-01	0,65	2,87E-01	2,83	2,09E-02	39,39	1,09E-01	0,98	3,79E-01	0,58
Q9NRV9	HEBP1_HUMAN Heme-binding protein 1	5	5	7,56E-01	1,32	7,18E-01	0,93	8,08E-01	1,14	5,02E-04	20,22	1,24E-02	1,00	6,61E-01	0,58
Q9UHQ9	NB5R1_HUMAN NADH-cytochrome b5 reductase 1	3	3	7,19E-01	0,63	9,04E-01	1,09	1,00E+00	1,00	1,49E-02	21,12	4,01E-02	0,99	3,98E-01	0,58
P12956	XRCC6_HUMAN X-ray repair cross-complementing protein 6	20	19	3,60E-01	0,20	4,44E-01	1,18	2,38E-01	8,28	2,27E-03	32,67	3,60E-04	1,00	3,09E-01	0,58
P43490	NAMPT_HUMAN Nicotinamide phosphoribosyltransferase	8	8	5,63E-01	0,44	6,29E-01	0,72	5,73E-01	0,31	1,04E-03	47,79	1,63E-02	0,99	3,91E-01	0,58
P06396	GELS_HUMAN Gelsolin	26	9	8,63E-01	0,78	2,92E-02	0,69	9,98E-01	1,00	5,90E-03	20,82	7,24E-03	1,00	1,02E-02	0,58
P22695	QCR2_HUMAN Cytochrome b-c1 complex subunit 2, mitochondrial	7	7	1,43E-01	0,24	3,04E-01	0,77	4,58E-01	0,42	4,42E-04	41,17	3,05E-06	1,00	3,57E-01	0,58
O43175	SERA_HUMAN D-3-phosphoglycerate dehydrogenase	11	11	2,34E-01	0,07	8,74E-03	0,73	1,00E+00	1,00	1,09E-01	3,10	1,50E-06	1,00	4,56E-03	0,58
P00352	AL1A1_HUMAN Retinal dehydrogenase 1	10	9	2,36E-01	1,42	4,00E-01	1,67	7,26E-01	0,81	9,24E-03	38,41	3,18E-01	0,88	3,61E-01	0,57
P47756	CAPZB_HUMAN F-actin-capping protein subunit beta	11	11	7,86E-01	1,72	3,00E-01	1,26	1,00E+00	1,00	1,04E-03	78,33	1,25E-02	1,00	5,03E-01	0,57
P06280	AGAL_HUMAN Alpha-galactosidase A	7	7	1,00E+00	1,00	1,79E-02	0,36	1,00E+00	1,00	2,58E-02	9,82	7,90E-01	0,60	2,45E-01	0,56
Q07812	BAX_HUMAN Apoptosis regulator BAX	5	5	1,50E-01	0,10	1,27E-01	0,79	5,81E-02	2,48	7,58E-02	3,60	5,61E-02	0,99	4,79E-02	0,56
P11586	C1TC_HUMAN C-1-	13	12	3,09E-	0,18	7,15E-	0,90	7,88E-	0,88	9,07E-	13,31	7,06E-	1,00	3,96E-	0,56

	tetrahydrofolate synthase, cytoplasmic			01		01		01		03		03		01	
Q5TZA2	CROCC_HUMAN Rootletin	2	2	1,18E-01	15,42	2,16E-03	9,23	3,62E-01	4,93	2,15E-01	2,70	1,79E-01	0,96	6,65E-01	0,56
O75340	PDCD6_HUMAN Programmed cell death protein 6	2	2	8,20E-01	0,72	7,50E-02	0,13	8,17E-05	0,02	3,13E-01	3,45	1,00E+00	0,50	1,77E-01	0,56
O95831	AIFM1_HUMAN Apoptosis-inducing factor 1, mitochondrial	10	9	3,45E-02	0,28	6,52E-01	0,79	1,14E-01	3,45	1,20E-02	17,85	5,65E-03	1,00	2,03E-01	0,56
Q12906	ILF3_HUMAN Interleukin enhancer-binding factor 3	14	14	1,98E-01	0,13	6,94E-01	0,87	1,08E-01	2,74	4,86E-02	3,28	3,67E-05	1,00	2,52E-01	0,56
Q9Y263	PLAP_HUMAN Phospholipase A-2-activating protein	3	3	7,84E-01	0,91	4,03E-02	0,40	1,00E+00	1,00	1,00E+00	1,00	2,72E-03	1,00	5,65E-01	0,56
P13473	LAMP2_HUMAN Lysosome-associated membrane glycoprotein 2	4	4	5,56E-01	0,60	5,61E-01	0,79	3,93E-01	1,53	1,32E-03	51,31	3,51E-02	0,99	4,55E-01	0,56
P50995	ANX11_HUMAN Annexin A11	13	13	2,05E-01	0,09	5,22E-01	0,89	6,46E-01	0,76	3,11E-02	64,40	2,43E-06	1,00	1,02E-01	0,55
P30040	ERP29_HUMAN Endoplasmic reticulum resident protein 29	12	12	2,15E-01	0,08	1,71E-01	0,81	6,11E-01	1,41	1,96E-05	135,12	5,41E-08	1,00	2,12E-02	0,55
P84085	ARF5_HUMAN ADP-ribosylation factor 5	7	2	2,79E-01	0,48	1,46E-02	0,20	1,00E+00	1,00	1,00E+00	1,00	1,00E+00	0,50	5,88E-01	0,55
O15400	STX7_HUMAN Syntaxin-7	5	5	2,16E-01	0,22	3,43E-01	0,83	1,00E+00	1,00	4,86E-06	80,01	4,44E-02	0,99	2,64E-02	0,55
P47755	CAZA2_HUMAN F-actin-capping protein subunit alpha-2	8	6	3,55E-02	0,06	3,56E-01	1,39	4,79E-01	0,33	1,51E-05	86,67	1,77E-01	0,96	3,03E-01	0,55
P05455	LA_HUMAN Lupus La protein	8	8	1,08E-02	0,02	1,93E-01	0,66	1,00E+00	1,00	1,00E+00	1,00	7,62E-02	0,98	4,61E-01	0,55

Q07020	RL18_HUMAN 60S ribosomal protein L18	7	7	4,70E-01	0,22	3,31E-01	1,13	6,04E-01	2,42	1,46E-03	31,55	3,48E-05	1,00	3,34E-01	0,54
P13686	PPA5_HUMAN Tartrate-resistant acid phosphatase type 5	4	4	1,80E-01	0,24	3,95E-01	0,57	5,75E-01	0,78	9,01E-06	66,47	1,10E-02	1,00	3,24E-01	0,54
Q96C19	EFHD2_HUMAN EF-hand domain-containing protein D2	3	3	1,00E+00	1,00	9,23E-01	0,94	1,00E+00	1,00	3,87E-01	3,46	5,59E-02	0,99	1,95E-01	0,54
Q9HC38	GLOD4_HUMAN Glyoxalase domain-containing protein 4	2	2	4,70E-01	0,49	4,39E-01	0,54	1,00E+00	1,00	4,48E-02	12,57	1,00E+00	0,50	5,62E-01	0,54
Q96KP4	CNDP2_HUMAN Cytosolic non-specific dipeptidase	12	12	6,63E-01	0,71	6,38E-01	1,13	1,19E-01	8,80	3,46E-02	5,87	1,99E-01	0,95	1,13E-01	0,53
P52565	GDIR1_HUMAN Rho GDP-dissociation inhibitor 1	11	11	2,53E-01	0,12	2,78E-01	0,68	4,75E-01	3,92	1,35E-05	81,68	9,86E-04	1,00	2,91E-02	0,53
P26599	PTBP1_HUMAN Polypyrimidine tract-binding protein 1	14	14	2,23E-03	0,03	6,89E-01	1,11	6,25E-01	1,28	8,45E-03	7,89	3,56E-02	0,99	3,83E-01	0,53
O15144	ARPC2_HUMAN Actin-related protein 2/3 complex subunit 2	14	14	5,15E-01	0,43	5,06E-01	0,85	9,21E-02	4,24	7,41E-06	175,87	4,21E-02	0,99	4,39E-02	0,53
Q9HCU5	PREB_HUMAN Prolactin regulatory element-binding protein	2	2	2,58E-01	0,67	2,90E-02	0,18	1,00E+00	1,00	1,00E+00	1,00	1,00E+00	0,50	4,18E-01	0,53
Q05682	CALD1_HUMAN Caldesmon	31	31	2,67E-01	0,11	3,47E-01	0,80	1,97E-01	2,21	5,06E-01	1,74	5,12E-04	1,00	1,42E-02	0,52
P84095	RHOG_HUMAN Rho-related GTP-binding protein RhoG	6	5	7,44E-01	1,17	5,05E-01	0,52	1,28E-01	2,33	1,57E-04	98,63	5,06E-01	0,76	4,10E-01	0,52
Q9NUQ9	CYRIB_HUMAN CYFIP-related Rac1 interactor B	3	2	2,00E-01	0,55	2,04E-01	0,36	9,06E-01	1,08	4,41E-04	50,53	1,68E-02	0,99	2,82E-01	0,51
Q9BVK6	TMED9_HUMAN Transmembrane emp24 domain-containing protein 9	9	8	4,71E-02	0,04	5,49E-01	0,67	1,00E+00	1,00	4,22E-01	2,80	7,52E-02	0,98	1,47E-01	0,51
P10253	LYAG_HUMAN Lysosomal alpha-glucosidase	6	6	4,40E-01	0,58	5,81E-01	0,66	5,99E-02	12,29	3,79E-02	24,65	2,47E-02	0,99	2,68E-01	0,51

Q96JY6	PDLI2_HUMAN PDZ and LIM domain protein 2	2	2	1,37E-01	0,18	3,21E-02	0,19	3,73E-01	1,54	1,90E-01	1,87	1,78E-02	0,99	6,56E-02	0,51
Q9UBG0	MRC2_HUMAN C-type mannose receptor 2	12	12	1,51E-01	0,19	2,47E-02	0,55	9,00E-01	0,91	1,00E+00	1,00	2,45E-01	0,93	5,61E-02	0,51
Q9BZQ8	NIBA1_HUMAN Protein Niban 1	3	3	2,50E-02	0,23	5,50E-01	0,50	1,00E+00	1,00	6,85E-02	3,44	6,73E-02	0,99	6,54E-01	0,51
O75165	DJC13_HUMAN DnaJ homolog subfamily C member 13	5	5	1,72E-01	0,35	8,38E-01	0,85	1,00E+00	1,00	1,00E+00	1,00	7,14E-02	0,99	1,86E-01	0,50
Q9BWM7	SFXN3_HUMAN Sideroflexin-3	11	11	9,27E-01	0,94	1,93E-01	0,38	3,93E-01	1,41	1,21E-05	132,14	1,28E-02	1,00	5,75E-01	0,50
P61224	RAP1B_HUMAN Ras-related protein Rap-1b	8	2	2,88E-01	0,32	8,73E-01	0,97	6,24E-01	2,19	3,43E-02	23,37	2,64E-01	0,92	3,19E-02	0,50
P37108	SRP14_HUMAN Signal recognition particle 14 kDa protein	3	3	3,04E-01	0,53	1,09E-02	0,56	1,00E+00	1,00	2,17E-01	1,45	3,06E-01	0,89	2,68E-02	0,50
Q15080	NCF4_HUMAN Neutrophil cytosol factor 4	2	2	8,04E-01	0,93	4,60E-01	0,67	1,00E+00	1,00	1,07E-01	4,93	1,00E+00	0,50	3,52E-02	0,50
P52789	HXK2_HUMAN Hexokinase-2	6	4	3,61E-01	0,43	6,31E-01	0,80	4,27E-01	0,51	1,90E-02	13,76	8,89E-03	1,00	2,91E-01	0,50
Q9HB40	RISC_HUMAN Retinoid-inducible serine carboxypeptidase	4	3	5,78E-01	0,53	5,75E-01	0,67	2,43E-01	3,75	2,09E-03	51,50	1,00E+00	0,50	3,87E-01	0,50
Q16891	MIC60_HUMAN MICOS complex subunit MIC60	14	13	2,18E-01	0,13	2,47E-01	0,88	4,53E-01	0,54	3,95E-03	39,06	1,19E-04	1,00	2,76E-01	0,50
Q02790	FKBP4_HUMAN Peptidyl-prolyl cis-trans isomerase FKBP4	3	2	6,10E-01	2,49	2,58E-01	0,26	9,04E-01	0,87	1,13E-02	6,06	1,43E-01	0,97	2,04E-01	0,49
Q15417	CNN3_HUMAN Calponin-3	12	11	2,75E-01	0,16	8,84E-03	0,54	8,17E-01	1,15	7,55E-01	0,77	2,24E-05	1,00	1,38E-02	0,49
P25787	PSA2_HUMAN Proteasome subunit alpha type-2	5	4	4,36E-01	0,28	3,23E-01	0,35	1,00E+00	1,00	1,56E-02	21,09	8,09E-02	0,98	1,68E-01	0,49

P11216	PYGB_HUMAN Glycogen phosphorylase, brain form	11	8	3,68E-02	0,08	1,56E-01	0,28	6,16E-01	1,50	9,40E-02	2,31	5,11E-01	0,76	3,65E-01	0,48
Q9NY33	DPP3_HUMAN Dipeptidyl peptidase 3	6	6	4,18E-01	0,35	2,99E-01	0,35	1,00E+00	1,00	6,34E-03	13,34	1,86E-01	0,95	1,28E-01	0,48
P29317	EPHA2_HUMAN Ephrin type-A receptor 2	5	5	4,95E-01	0,62	4,53E-02	0,31	1,00E+00	1,00	6,01E-02	2,37	1,29E-02	1,00	4,84E-01	0,47
P61970	NTF2_HUMAN Nuclear transport factor 2	2	2	3,39E-01	0,50	1,95E-01	0,30	1,00E+00	1,00	1,00E+00	1,00	1,60E-02	1,00	2,63E-02	0,47
P08754	GNAI3_HUMAN Guanine nucleotide-binding protein G(i) subunit alpha	4	1	1,13E-01	0,14	8,47E-01	0,94	1,00E+00	1,00	9,97E-03	25,37	7,00E-02	0,99	2,45E-01	0,47
P00387	NB5R3_HUMAN NADH-cytochrome b5 reductase 3	13	13	1,09E-01	0,10	4,04E-02	0,62	1,92E-01	2,37	1,71E-02	15,44	1,06E-03	1,00	1,33E-02	0,47
P23381	SYWC_HUMAN Tryptophan--tRNA ligase, cytoplasmic	13	13	2,40E-01	0,13	1,01E-01	0,71	3,17E-01	3,08	7,91E-07	205,57	1,51E-02	1,00	4,77E-03	0,46
P05141	ADT2_HUMAN ADP/ATP translocase 2	8	3	1,58E-01	0,07	6,03E-01	1,07	6,59E-01	1,66	3,53E-04	201,93	1,39E-02	1,00	5,58E-01	0,46
Q15843	NEDD8_HUMAN NEDD8	2	2	2,04E-01	0,31	6,46E-01	0,82	1,00E+00	1,00	1,00E+00	1,00	1,92E-02	0,99	4,07E-01	0,45
Q92747	ARC1A_HUMAN Actin-related protein 2/3 complex subunit 1A	3	2	1,41E-01	0,15	4,30E-01	0,48	1,00E+00	1,00	2,06E-01	4,13	1,74E-02	0,99	5,40E-01	0,45
Q14108	SCRB2_HUMAN Lysosome membrane protein 2	8	8	2,91E-01	0,27	4,84E-01	0,72	1,00E+00	1,00	2,09E-06	104,81	4,43E-02	0,99	7,65E-02	0,45
P09110	THIK_HUMAN 3-ketoacyl-CoA thiolase, peroxisomal	4	4	2,53E-01	3,00	2,63E-02	0,66	3,72E-01	4,72	4,06E-06	84,00	1,00E+00	0,50	5,03E-01	0,45
O95782	AP2A1_HUMAN AP-2 complex subunit alpha-1	9	6	1,29E-01	0,17	4,16E-02	0,39	8,40E-01	0,84	3,66E-01	2,84	2,77E-03	1,00	2,05E-02	0,45
Q9UGI8	TES_HUMAN Testin	5	5	2,53E-01	0,21	4,85E-01	0,61	6,15E-01	0,63	7,64E-06	18,64	8,59E-01	0,57	3,22E-01	0,45
Q15165	PON2_HUMAN Serum paraoxonase/arylesterase 2	3	3	1,43E-01	9,76	4,08E-01	0,43	1,00E+00	1,00	3,42E-02	2,08	1,50E-01	0,97	2,97E-01	0,45

P67936	TPM4_HUMAN Tropomyosin alpha-4 chain	27	14	1,94E-01	0,08	1,95E-01	0,73	6,93E-01	0,74	3,04E-02	4,79	5,71E-03	1,00	6,56E-03	0,45
P21281	VATB2_HUMAN V-type proton ATPase subunit B, brain isoform	10	10	2,05E-01	0,33	9,60E-01	1,02	1,18E-01	1,98	2,07E-05	239,69	1,66E-01	0,96	5,29E-01	0,44
P30043	BLVRB_HUMAN Flavin reductase (NADPH)	4	4	1,45E-01	0,24	6,12E-01	0,74	7,35E-01	0,71	1,02E-02	32,39	5,98E-02	0,99	4,96E-01	0,44
P01111	RASN_HUMAN GTPase NRas	4	4	1,93E-01	0,13	6,02E-01	1,56	1,00E+00	1,00	2,19E-02	9,76	1,36E-02	1,00	1,82E-01	0,44
P21730	C5AR1_HUMAN C5a anaphylatoxin chemotactic receptor 1	2	2	1,54E-01	6,47	4,42E-01	0,34	2,68E-01	0,09	2,40E-03	133,08	1,44E-02	1,00	3,16E-01	0,44
O15031	PLXB2_HUMAN Plexin-B2	5	5	6,97E-01	1,59	5,99E-03	0,15	7,58E-01	1,37	3,07E-02	7,05	1,51E-01	0,97	5,69E-01	0,44
Q99798	ACON_HUMAN Aconitate hydratase, mitochondrial	15	14	2,19E-01	0,23	1,07E-01	0,43	9,61E-01	1,03	1,75E-06	120,39	1,96E-03	1,00	4,02E-02	0,43
Q12931	TRAP1_HUMAN Heat shock protein 75 kDa, mitochondrial	5	5	2,88E-01	0,41	9,45E-01	0,95	1,00E+00	1,00	3,90E-02	5,13	5,52E-03	1,00	4,62E-01	0,43
Q9NVI7	ATD3A_HUMAN ATPase family AAA domain-containing protein 3A	6	2	9,40E-03	0,09	2,03E-01	0,65	1,00E+00	1,00	1,41E-01	2,06	2,90E-02	0,99	4,12E-01	0,43
Q96M27	PRRC1_HUMAN Protein PRRC1	4	4	2,44E-01	0,14	6,34E-01	0,90	3,17E-01	0,21	2,73E-01	0,31	8,66E-02	0,98	2,54E-02	0,43
P17050	NAGAB_HUMAN Alpha-N-acetylgalactosaminidase	3	3	1,36E-01	0,17	4,51E-01	0,50	1,01E-01	2,53	7,05E-02	7,63	2,39E-03	1,00	2,40E-01	0,43
Q9H3S7	PTN23_HUMAN Tyrosine-protein phosphatase non-receptor type 23	2	2	2,42E-01	0,41	4,82E-02	3,43	1,00E+00	1,00	2,85E-01	1,92	7,80E-02	0,98	2,18E-01	0,43
P22626	ROA2_HUMAN Heterogeneous nuclear ribonucleoproteins A2/B1	14	14	4,79E-01	0,47	3,55E-01	0,79	8,64E-01	1,12	3,79E-03	46,95	8,20E-02	0,98	1,35E-01	0,43
P61916	NPC2_HUMAN NPC intracellular cholesterol	2	2	5,09E-01	2,33	5,77E-01	1,76	6,67E-01	0,57	8,53E-03	29,90	6,97E-02	0,99	2,89E-01	0,43

	transporter 2														
Q13561	DCTN2_HUMAN Dynactin subunit 2	11	11	6,27E-01	0,48	8,52E-01	1,08	6,46E-01	0,70	2,79E-02	9,50	1,47E-02	1,00	2,27E-01	0,42
Q15363	TMED2_HUMAN Transmembrane emp24 domain-containing protein 2	3	3	1,51E-01	0,11	1,03E-01	0,48	3,93E-01	0,53	2,70E-01	2,28	4,40E-02	0,99	1,24E-01	0,42
O15127	SCAM2_HUMAN Secretory carrier-associated membrane protein 2	3	3	2,41E-01	0,70	3,45E-01	0,46	1,33E-01	2,99	9,89E-07	108,90	1,00E+00	0,50	4,80E-01	0,42
P33176	KINH_HUMAN Kinesin-1 heavy chain	19	19	1,95E-01	0,17	2,24E-02	0,63	6,59E-01	1,19	4,26E-01	0,53	2,22E-03	1,00	5,20E-02	0,42
P17174	AATC_HUMAN Aspartate aminotransferase, cytoplasmic	5	5	3,74E-01	0,18	9,04E-02	0,60	1,00E+00	1,00	1,97E-02	16,70	3,28E-03	1,00	1,78E-01	0,42
Q96N66	MBOA7_HUMAN Lysophospholipid acyltransferase 7	2	2	7,68E-01	0,67	6,75E-01	0,85	1,00E+00	1,00	1,52E-01	4,14	2,05E-02	0,99	3,72E-01	0,42
P46926	GNPI1_HUMAN Glucosamine-6-phosphate isomerase 1	3	3	4,88E-01	0,50	5,27E-01	0,60	3,57E-01	0,27	1,93E-02	16,24	1,35E-01	0,97	4,78E-01	0,42
P56134	ATPK_HUMAN ATP synthase subunit f, mitochondrial	2	2	1,07E-01	0,18	3,03E-01	0,47	2,39E-02	5,07	1,27E-03	34,12	1,04E-01	0,98	3,96E-01	0,42
P26640	SYVC_HUMAN Valine--tRNA ligase	12	12	5,68E-01	0,41	9,53E-02	0,43	1,56E-01	0,60	1,63E-02	10,26	1,06E-01	0,98	5,63E-01	0,42
Q9HB07	MYG1_HUMAN UPF0160 protein MYG1, mitochondrial	3	3	3,91E-01	0,36	1,91E-01	0,47	1,00E+00	1,00	1,00E+00	1,00	3,83E-02	0,99	1,83E-02	0,42
P49588	SYAC_HUMAN Alanine--tRNA ligase, cytoplasmic	24	24	3,90E-01	0,26	5,77E-02	0,57	3,75E-02	0,25	3,01E-01	3,42	2,27E-03	1,00	2,38E-02	0,42
P25786	PSA1_HUMAN Proteasome subunit alpha type-1	10	9	3,30E-01	0,18	3,78E-01	1,51	2,28E-01	0,16	3,04E-02	18,04	2,06E-01	0,95	9,34E-02	0,41
P49589	SYCC_HUMAN Cysteine--tRNA ligase, cytoplasmic	10	10	5,27E-01	0,41	3,67E-02	0,54	1,58E-01	3,79	8,43E-01	1,18	5,25E-02	0,99	5,31E-03	0,40

Q9Y6C9	MTCH2_HUMAN Mitochondrial carrier homolog 2	4	4	1,62E-01	0,34	4,71E-01	0,75	1,00E+00	1,00	3,47E-05	32,27	8,94E-02	0,98	2,04E-01	0,40
P61106	RAB14_HUMAN Ras-related protein Rab-14	6	6	2,13E-01	0,11	1,50E-01	0,74	7,28E-01	0,72	8,03E-06	121,32	1,99E-02	0,99	8,13E-02	0,40
Q9H4A4	AMPB_HUMAN Aminopeptidase B	4	4	4,95E-01	0,48	3,29E-01	0,45	1,00E+00	1,00	1,54E-05	42,12	7,70E-01	0,61	2,87E-01	0,40
Q9NYU2	UGGG1_HUMAN UDP-glucose:glycoprotein glucosyltransferase 1	19	18	8,54E-01	0,79	5,10E-01	0,70	9,64E-01	1,03	4,86E-03	8,93	2,73E-02	0,99	3,41E-02	0,40
Q9UIB8	SLAF5_HUMAN SLAM family member 5	6	6	2,45E-01	0,53	5,48E-02	2,85	1,00E+00	1,00	6,97E-04	30,61	1,32E-01	0,97	1,70E-01	0,40
Q9NZM1	MYOF_HUMAN Myoferlin	46	44	6,04E-01	0,52	1,19E-01	0,56	8,51E-01	0,86	8,04E-03	11,38	1,88E-02	0,99	1,16E-01	0,40
P25774	CATS_HUMAN Cathepsin S	5	5	1,95E-01	0,06	2,04E-01	5,17	7,25E-01	2,05	3,30E-04	310,42	1,26E-05	1,00	3,01E-01	0,39
O14828	SCAM3_HUMAN Secretory carrier-associated membrane protein 3	5	5	5,08E-01	0,34	1,78E-01	0,80	1,00E+00	1,00	2,51E-06	54,78	4,45E-02	0,99	3,06E-01	0,39
P29144	TPP2_HUMAN Tripeptidyl-peptidase 2	3	2	2,55E-01	2,66	4,40E-02	0,21	8,30E-01	0,86	1,38E-01	1,71	1,34E-02	1,00	3,06E-01	0,39
O15305	PMM2_HUMAN Phosphomannomutase 2	2	2	6,19E-01	0,55	8,50E-02	0,78	1,00E+00	1,00	1,15E-04	11,84	1,33E-01	0,97	5,30E-02	0,39
P46939	UTRO_HUMAN Utrophin	4	4	1,44E-01	0,12	4,06E-02	0,21	1,00E+00	1,00	1,00E+00	1,00	9,06E-02	0,98	3,39E-01	0,38
P08195	4F2_HUMAN 4F2 cell-surface antigen heavy chain	22	22	1,07E-01	0,12	5,61E-01	0,87	1,36E-02	9,57	5,94E-02	6,49	7,75E-03	1,00	6,59E-02	0,38
Q08188	TGM3_HUMAN Protein-glutamine gamma-glutamyltransferase E	4	4	4,31E-01	0,48	2,07E-01	0,45	4,47E-01	1,94	5,00E-01	0,30	1,13E-01	0,98	1,98E-02	0,38
Q8TCT9	HM13_HUMAN Minor histocompatibility antigen H13	7	6	1,39E-01	0,25	4,82E-01	0,78	1,00E+00	1,00	1,14E-03	93,49	3,44E-02	0,99	3,26E-01	0,38

P61254	RL26_HUMAN 60S ribosomal protein L26	6	6	3,16E-01	3,39	3,83E-01	0,55	2,79E-02	0,02	1,21E-01	0,43	1,88E-01	0,95	1,66E-01	0,38
O95373	IPO7_HUMAN Importin-7	4	4	3,36E-02	0,07	3,55E-01	0,59	9,44E-01	0,94	7,73E-02	4,99	1,02E-01	0,98	4,36E-01	0,37
O75643	U520_HUMAN U5 small nuclear ribonucleoprotein 200 kDa helicase	4	4	3,22E-02	0,39	9,23E-01	0,89	1,00E+00	1,00	1,19E-01	0,33	1,98E-02	0,99	3,10E-01	0,37
P17844	DDX5_HUMAN Probable ATP-dependent RNA helicase DDX5	13	9	2,19E-01	0,15	5,84E-01	0,79	5,52E-01	0,53	2,94E-02	4,10	2,51E-03	1,00	2,11E-01	0,37
P49720	PSB3_HUMAN Proteasome subunit beta type-3	4	4	2,11E-01	0,11	1,58E-01	0,61	1,22E-01	6,93	5,15E-02	9,81	3,21E-06	1,00	8,36E-03	0,37
P07602	SAP_HUMAN Prosaposin	9	9	3,22E-01	0,23	4,06E-01	0,45	4,91E-01	1,33	1,63E-05	204,96	3,82E-01	0,84	4,41E-01	0,37
Q71U36	TBA1A_HUMAN Tubulin alpha-1A chain	26	1	3,08E-01	0,16	7,45E-01	1,24	3,38E-02	18,27	4,70E-04	50,19	7,94E-02	0,98	4,90E-01	0,36
Q9Y6M1	IF2B2_HUMAN Insulin-like growth factor 2 mRNA-binding protein 2	9	8	1,86E-01	0,20	2,85E-01	0,47	2,54E-01	0,34	1,09E-01	0,42	4,35E-01	0,81	1,56E-02	0,36
P07203	GPX1_HUMAN Glutathione peroxidase 1	4	4	3,43E-01	0,34	3,01E-01	0,63	1,00E+00	1,00	6,18E-03	79,33	6,63E-02	0,99	1,11E-02	0,36
P61221	ABCE1_HUMAN ATP-binding cassette sub-family E member 1	4	4	6,08E-01	0,47	5,20E-01	1,87	9,62E-01	0,94	3,92E-02	0,09	7,40E-01	0,62	5,57E-01	0,36
P21283	VATC1_HUMAN V-type proton ATPase subunit C 1	5	5	1,55E-03	0,15	8,42E-01	1,10	8,57E-02	8,67	5,59E-04	33,42	4,67E-02	0,99	1,56E-01	0,35
P07741	APT_HUMAN Adenine phosphoribosyltransferase	5	5	1,98E-01	0,08	1,74E-01	0,68	1,90E-01	3,82	4,18E-05	60,36	1,19E-01	0,98	5,52E-01	0,35
P62854	RS26_HUMAN 40S ribosomal protein S26	2	2	2,91E-01	2,04	4,23E-01	0,62	1,00E+00	1,00	3,30E-02	1,70	1,76E-01	0,96	7,82E-02	0,34
Q13283	G3BP1_HUMAN Ras GTPase-activating protein-binding protein 1	7	7	1,83E-01	0,33	3,67E-01	0,42	5,28E-01	1,27	1,30E-02	3,29	6,07E-02	0,99	3,68E-01	0,34

Q99436	PSB7_HUMAN Proteasome subunit beta type-7	5	5	2,35E-01	0,22	2,12E-01	0,26	8,40E-01	0,85	1,91E-02	6,16	6,73E-02	0,99	5,45E-02	0,34
Q16555	DPYL2_HUMAN Dihydropyrimidinase-related protein 2	14	13	1,64E-01	0,12	1,92E-01	0,37	1,08E-01	1,87	1,35E-04	28,25	1,56E-02	1,00	7,71E-03	0,34
Q99715	COCA1_HUMAN Collagen alpha-1(XII) chain	77	76	1,73E-02	0,01	1,33E-01	0,75	7,42E-01	0,72	2,50E-01	0,59	1,29E-01	0,97	1,25E-01	0,34
P35625	TIMP3_HUMAN Metalloproteinase inhibitor 3	5	5	2,18E-01	0,24	1,35E-02	0,20	9,23E-01	0,92	6,97E-01	0,65	4,73E-02	0,99	1,52E-01	0,34
Q15691	MARE1_HUMAN Microtubule-associated protein RP/EB family member 1	11	10	2,09E-01	0,09	3,00E-01	1,13	1,00E+00	1,00	1,49E-06	67,80	4,68E-02	0,99	5,16E-02	0,34
P62701	RS4X_HUMAN 40S ribosomal protein S4, X isoform	9	9	2,75E-01	0,11	7,63E-01	0,91	5,18E-01	1,50	6,89E-01	0,71	2,00E-02	0,99	4,51E-02	0,34
P51148	RAB5C_HUMAN Ras-related protein Rab-5C	6	4	6,27E-01	1,68	5,51E-01	0,82	6,15E-01	1,65	6,50E-06	95,10	6,90E-02	0,99	1,11E-01	0,34
Q8N1G4	LRC47_HUMAN Leucine-rich repeat-containing protein 47	4	4	2,79E-01	0,42	4,00E-01	0,58	8,42E-01	1,12	3,96E-02	1,57	1,31E-01	0,97	1,89E-01	0,34
Q9H223	EHD4_HUMAN EH domain-containing protein 4	7	6	4,39E-01	0,35	2,67E-01	0,71	6,56E-01	0,57	3,15E-06	73,47	4,50E-01	0,80	3,11E-01	0,33
Q9BR76	COR1B_HUMAN Coronin-1B	6	6	6,58E-02	0,21	6,23E-01	0,58	1,00E+00	1,00	2,67E-02	24,98	5,75E-02	0,99	4,12E-01	0,33
Q9NZ08	ERAP1_HUMAN Endoplasmic reticulum aminopeptidase 1	3	3	7,88E-02	0,25	5,44E-02	0,41	1,00E+00	1,00	2,25E-02	12,88	1,00E+00	0,50	1,04E-02	0,33
P50570	DYN2_HUMAN Dynamin-2	10	10	2,38E-02	6,07	6,48E-01	1,13	8,41E-01	1,36	2,59E-01	4,66	9,28E-01	0,53	2,23E-01	0,33
P35998	PRS7_HUMAN 26S proteasome regulatory subunit 7	14	14	2,46E-01	0,25	8,01E-01	0,88	6,19E-01	1,35	4,42E-02	7,65	1,50E-04	1,00	2,85E-01	0,33

P17931	LEG3_HUMAN Galectin-3	7	7	3,35E-01	0,46	3,64E-01	0,45	7,92E-01	1,28	9,24E-08	309,46	4,81E-02	0,99	2,81E-01	0,32
Q3ZCM7	TBB8_HUMAN Tubulin beta-8 chain	9	1	1,00E+00	1,00	3,66E-02	0,42	1,00E+00	1,00	1,00E+00	1,00	1,00E+00	0,50	1,89E-01	0,32
P38606	VATA_HUMAN V-type proton ATPase catalytic subunit A	17	17	5,21E-01	0,48	3,01E-01	0,84	5,05E-01	1,88	1,23E-05	261,22	9,19E-02	0,98	2,56E-01	0,32
Q8WWI1	LMO7_HUMAN LIM domain only protein 7	23	23	1,82E-01	0,36	1,67E-02	0,31	9,61E-01	0,95	7,36E-01	0,69	1,08E-01	0,98	1,13E-01	0,32
P30419	NMT1_HUMAN Glycylpeptide N-tetradecanoyltransferase 1	3	3	7,47E-01	0,63	8,01E-01	0,92	3,53E-02	2,06	1,00E+00	1,00	3,57E-03	1,00	2,75E-01	0,32
Q7Z570	Z804A_HUMAN Zinc finger protein 804A	2	2	4,68E-02	0,13	4,66E-01	0,63	1,00E+00	1,00	1,55E-01	3,01	1,15E-01	0,98	1,94E-01	0,32
P23368	MAOM_HUMAN NAD-dependent malic enzyme, mitochondrial	8	7	3,46E-01	0,43	6,17E-01	0,59	1,13E-01	3,09	1,25E-03	57,87	9,71E-01	0,51	8,75E-02	0,32
Q04446	GLGB_HUMAN 1,4-alpha-glucan-branching enzyme	7	7	2,51E-01	0,48	7,64E-02	0,52	9,14E-01	0,91	5,93E-03	4,93	8,27E-04	1,00	4,20E-02	0,32
Q00765	REEP5_HUMAN Receptor expression-enhancing protein 5	3	3	6,77E-01	0,58	3,90E-01	0,51	1,52E-02	22,36	1,05E-01	9,72	1,04E-01	0,98	2,61E-01	0,31
Q9NR12	PDLI7_HUMAN PDZ and LIM domain protein 7	12	11	2,81E-01	0,11	6,17E-01	0,86	2,24E-01	2,91	2,64E-01	1,92	1,18E-01	0,98	7,82E-03	0,31
Q96CX2	KCD12_HUMAN BTB/POZ domain-containing protein KCTD12	3	3	9,36E-01	1,03	1,41E-01	2,81	1,78E-01	2,59	2,83E-02	12,62	8,74E-03	1,00	2,15E-01	0,31
Q99653	CHP1_HUMAN Calcineurin B homologous protein 1	4	4	4,11E-01	0,23	5,31E-01	0,56	1,48E-01	22,41	7,79E-03	16,96	9,84E-01	0,51	4,78E-01	0,31
O43776	SYNC_HUMAN Asparagine--tRNA ligase, cytoplasmic	11	11	1,33E-01	0,19	2,39E-01	0,65	5,27E-01	0,16	2,40E-01	2,81	9,39E-01	0,53	2,60E-01	0,31
P62753	RS6_HUMAN 40S ribosomal protein S6	7	7	5,47E-01	0,31	2,25E-01	1,38	6,18E-01	2,26	4,39E-01	2,66	2,27E-02	0,99	1,92E-01	0,31

O15270	SPTC2_HUMAN Serine palmitoyltransferase 2	2	2	1,31E-01	0,26	7,69E-01	0,76	1,00E+00	1,00	7,89E-03	3,47	1,00E+00	0,50	2,82E-01	0,30
P05120	PAI2_HUMAN Plasminogen activator inhibitor 2	5	5	7,63E-01	0,57	8,48E-01	0,82	4,64E-01	3,90	3,61E-02	18,25	3,91E-03	1,00	1,96E-01	0,30
Q13596	SNX1_HUMAN Sorting nexin-1	5	3	5,28E-02	0,13	9,83E-01	1,00	2,07E-01	5,08	7,12E-05	42,87	2,98E-02	0,99	5,23E-02	0,30
P25789	PSA4_HUMAN Proteasome subunit alpha type-4	4	4	5,76E-01	0,48	3,81E-01	0,44	1,00E+00	1,00	1,43E-02	10,11	4,04E-02	0,99	3,10E-01	0,30
P69849	NOM3_HUMAN Nodal modulator 3	7	7	5,85E-01	0,63	3,15E-01	0,39	4,92E-01	0,58	3,39E-01	1,51	9,14E-02	0,98	7,21E-04	0,30
O95571	ETHE1_HUMAN Persulfide dioxygenase ETHE1, mitochondrial	2	2	1,00E+00	1,00	8,18E-01	0,82	1,00E+00	1,00	9,02E-02	3,17	1,00E+00	0,50	2,92E-02	0,30
Q9BXF6	RFIP5_HUMAN Rab11 family-interacting protein 5	3	3	8,51E-01	1,09	4,26E-01	0,58	1,00E+00	1,00	1,23E-01	2,93	1,00E+00	0,50	3,18E-02	0,29
Q13740	CD166_HUMAN CD166 antigen	12	12	4,88E-01	0,38	1,45E-02	0,62	6,92E-01	0,71	1,42E-03	29,32	2,54E-02	0,99	8,76E-02	0,29
Q9Y5X3	SNX5_HUMAN Sorting nexin-5	4	4	2,44E-01	4,43	1,03E-01	0,27	6,54E-01	0,64	1,42E-04	68,54	9,85E-02	0,98	2,60E-01	0,29
P05121	PAI1_HUMAN Plasminogen activator inhibitor 1	5	5	3,21E-01	0,26	2,30E-01	1,44	9,60E-01	1,03	4,13E-01	0,39	1,96E-01	0,95	3,70E-02	0,29
P55795	HNRH2_HUMAN Heterogeneous nuclear ribonucleoprotein H2	6	3	1,94E-01	0,43	4,56E-01	0,65	9,75E-01	1,03	3,75E-02	3,26	2,55E-02	0,99	1,07E-01	0,29
Q93052	LPP_HUMAN Lipoma-preferred partner	2	2	1,55E-01	0,27	8,51E-01	0,83	1,00E+00	1,00	3,13E-01	3,41	1,45E-02	1,00	1,53E-01	0,28
P05107	ITB2_HUMAN Integrin beta-2	21	21	4,93E-01	0,60	3,29E-01	0,46	4,04E-01	4,06	1,27E-06	870,39	2,56E-01	0,92	1,31E-01	0,27
Q12905	ILF2_HUMAN Interleukin enhancer-binding factor 2	6	6	1,54E-01	0,18	2,24E-01	1,80	6,34E-01	0,68	2,01E-01	1,81	4,40E-02	0,99	2,80E-02	0,27
P47895	AL1A3_HUMAN Aldehyde dehydrogenase family 1	6	6	7,79E-01	0,77	1,01E-01	0,35	2,28E-01	0,37	2,15E-01	2,09	1,36E-03	1,00	1,32E-01	0,27

	member A3														
Q9BRF8	CPPED_HUMAN Serine/threonine-protein phosphatase CPPED1	4	4	5,40E-01	0,45	5,14E-01	0,84	1,00E+00	1,00	1,98E-06	32,76	8,19E-02	0,98	3,13E-01	0,27
Q8NCA5	FA98A_HUMAN Protein FAM98A	3	3	3,66E-01	0,41	3,15E-01	0,26	4,12E-01	0,38	4,43E-02	4,82	4,48E-02	0,99	1,25E-01	0,27
Q9NP79	VTA1_HUMAN Vacuolar protein sorting-associated protein VTA1 homolog	3	3	1,44E-01	0,08	2,66E-03	0,44	1,00E+00	1,00	1,00E+00	1,00	1,89E-02	0,99	3,58E-01	0,27
O75223	GGCT_HUMAN Gamma-glutamylcyclotransferase	2	2	4,09E-01	0,60	4,19E-01	0,69	1,00E+00	1,00	9,18E-01	0,87	1,00E+00	0,50	3,47E-02	0,27
Q86TX2	ACOT1_HUMAN Acyl-coenzyme A thioesterase 1	3	3	9,40E-01	1,07	6,78E-01	0,83	1,00E+00	1,00	1,06E-01	2,27	1,59E-02	1,00	7,48E-03	0,26
Q96TA1	NIBA2_HUMAN Protein Niban 2	8	8	7,19E-01	0,57	3,49E-01	0,79	1,24E-01	1,66	1,11E-03	26,25	2,51E-01	0,92	3,25E-02	0,26
Q02318	CP27A_HUMAN Sterol 26-hydroxylase, mitochondrial	13	12	5,46E-01	0,64	1,56E-01	2,80	1,38E-01	4,08	1,79E-05	602,73	6,57E-01	0,67	1,12E-01	0,26
O95302	FKBP9_HUMAN Peptidyl-prolyl cis-trans isomerase FKBP9	10	10	6,29E-02	0,11	7,69E-01	0,89	4,10E-01	0,41	1,65E-02	8,53	9,59E-04	1,00	7,56E-03	0,26
P41252	SYIC_HUMAN Isoleucine--tRNA ligase, cytoplasmic	10	10	2,31E-01	0,15	3,32E-01	0,43	3,72E-01	0,36	5,35E-01	1,90	6,92E-02	0,99	3,60E-02	0,26
Q99829	CPNE1_HUMAN Copine-1	6	6	1,03E-01	0,19	2,56E-01	0,57	9,35E-01	0,94	4,26E-02	2,27	1,44E-01	0,97	8,61E-02	0,26
P06865	HEXA_HUMAN Beta-hexosaminidase subunit alpha	8	8	4,68E-02	0,27	1,69E-01	0,37	1,00E+00	1,00	1,87E-02	24,45	6,93E-02	0,99	2,38E-01	0,26
P04179	SODM_HUMAN Superoxide dismutase [Mn], mitochondrial	6	6	3,97E-01	0,36	9,39E-01	1,04	2,88E-01	8,12	1,07E-02	22,69	1,04E-02	1,00	5,44E-02	0,26
Q13423	NNTM_HUMAN NAD(P) transhydrogenase, mitochondrial	13	12	5,93E-01	2,58	5,25E-01	0,56	3,78E-01	3,50	7,76E-05	113,51	7,17E-03	1,00	9,59E-02	0,26
Q13011	ECH1_HUMAN Delta(3,5)-	6	6	4,61E-	0,13	1,12E-	0,27	6,22E-	0,61	1,68E-	5,75	6,24E-	0,99	1,39E-	0,25

	Delta(2,4)-dienoyl-CoA isomerase, mitochondrial			02		01		01		01		02		01	
P53582	MAP11_HUMAN Methionine aminopeptidase 1	5	5	2,12E-01	0,31	9,88E-01	1,00	1,00E+00	1,00	2,98E-01	0,35	5,77E-05	1,00	2,96E-01	0,25
O95292	VAPB_HUMAN Vesicle-associated membrane protein-associated protein B/C	6	5	2,65E-01	0,26	4,74E-01	0,50	1,00E+00	1,00	1,57E-01	4,83	1,90E-02	0,99	2,22E-01	0,25
P55010	IF5_HUMAN Eukaryotic translation initiation factor 5	7	7	4,00E-01	0,57	3,71E-01	1,29	6,72E-02	0,10	7,84E-01	1,45	3,36E-02	0,99	1,98E-01	0,25
Q04760	LGUL_HUMAN Lactoylglutathione lyase	4	4	3,36E-01	0,31	6,01E-01	0,55	3,78E-01	1,57	3,90E-02	5,24	9,48E-03	1,00	1,41E-01	0,25
Q9BT78	CSN4_HUMAN COP9 signalosome complex subunit 4	9	9	8,34E-01	0,78	7,19E-01	0,76	1,00E+00	1,00	1,23E-02	6,34	3,86E-02	0,99	2,41E-01	0,25
P27695	APEX1_HUMAN DNA-(apurinic or apyrimidinic site) lyase	7	7	1,07E-02	0,02	5,15E-01	0,85	1,00E+00	1,00	7,09E-01	1,40	1,34E-02	1,00	2,67E-01	0,25
Q12907	LMAN2_HUMAN Vesicular integral-membrane protein VIP36	11	11	1,88E-01	0,08	9,89E-01	1,00	1,00E+00	1,00	1,34E-05	55,66	1,12E-02	1,00	1,89E-01	0,25
Q04323	UBXN1_HUMAN UBX domain-containing protein 1	2	2	8,13E-01	1,37	3,02E-01	1,90	1,74E-01	5,63	2,15E-01	1,69	6,05E-01	0,70	1,83E-01	0,24
O43488	ARK72_HUMAN Aflatoxin B1 aldehyde reductase member 2	3	3	3,09E-01	0,26	4,82E-01	0,90	1,00E+00	1,00	9,76E-06	71,15	1,00E+00	0,50	1,10E-01	0,24
P49721	PSB2_HUMAN Proteasome subunit beta type-2	5	5	6,89E-02	0,15	7,76E-01	0,89	4,40E-01	2,27	3,63E-05	14,02	7,79E-02	0,98	7,86E-02	0,24
P35580	MYH10_HUMAN Myosin-10	48	36	1,40E-01	0,06	2,26E-01	0,85	9,18E-01	0,92	3,90E-01	0,44	1,77E-02	0,99	1,54E-02	0,24
P19623	SPEE_HUMAN Spermidine synthase	4	4	4,22E-01	0,32	6,04E-01	1,58	6,37E-02	0,16	8,65E-01	0,78	1,45E-01	0,97	4,41E-02	0,24
Q8TEA8	DTD1_HUMAN D-aminoacyl-tRNA deacylase 1	2	2	7,38E-01	0,62	5,55E-01	0,62	1,00E+00	1,00	2,55E-02	6,61	2,01E-01	0,95	2,48E-01	0,24

P46940	IQGA1_HUMAN Ras GTPase-activating-like protein IQGAP1	61	57	8,40E-02	0,04	3,67E-01	0,87	2,99E-01	3,38	5,05E-06	81,48	2,30E-01	0,93	2,42E-03	0,24
P00390	GSHR_HUMAN Glutathione reductase, mitochondrial	8	8	1,81E-01	0,33	6,20E-01	0,62	4,56E-01	2,93	1,06E-02	24,48	1,15E-01	0,98	8,89E-02	0,23
O75718	CRTAP_HUMAN Cartilage-associated protein	13	13	1,63E-01	0,20	4,33E-01	0,64	8,44E-01	0,81	6,75E-01	1,84	8,87E-01	0,55	4,39E-02	0,23
O75533	SF3B1_HUMAN Splicing factor 3B subunit 1	6	6	3,19E-03	61,37	4,56E-01	0,47	1,10E-01	0,09	8,55E-01	1,15	5,93E-03	1,00	2,46E-01	0,23
Q8IWU6	SULF1_HUMAN Extracellular sulfatase Sulf-1	3	3	1,27E-01	0,11	6,70E-02	0,46	7,38E-01	1,38	1,13E-01	4,58	1,67E-01	0,96	2,10E-02	0,23
Q9Y224	RTRAF_HUMAN RNA transcription, translation and transport factor protein	6	6	5,82E-02	0,14	3,47E-01	0,38	1,00E+00	1,00	1,94E-01	1,28	6,83E-01	0,65	1,76E-01	0,22
P52790	HXK3_HUMAN Hexokinase-3	15	12	9,32E-01	1,07	1,17E-01	0,58	8,06E-01	0,78	2,60E-04	231,12	1,37E-01	0,97	1,05E-01	0,22
P51858	HDGF_HUMAN Hepatoma-derived growth factor	2	2	1,04E-01	0,29	2,43E-03	0,27	1,00E+00	1,00	1,00E+00	1,00	1,29E-02	1,00	2,40E-02	0,22
O43765	SGTA_HUMAN Small glutamine-rich tetratricopeptide repeat-containing protein alpha	5	5	7,12E-01	0,65	6,45E-01	0,92	1,00E+00	1,00	1,00E+00	1,00	4,38E-02	0,99	7,55E-02	0,22
P47813	IF1AX_HUMAN Eukaryotic translation initiation factor 1A, X-chromosomal	3	3	1,61E-01	0,33	7,86E-03	0,21	3,64E-01	0,49	1,00E+00	1,00	1,00E+00	0,50	1,04E-01	0,22
Q96E39	RMXL1_HUMAN RNA binding motif protein, X-linked-like-1	2	2	3,61E-01	0,39	6,00E-01	0,71	1,00E+00	1,00	4,04E-02	5,62	2,95E-02	0,99	2,71E-01	0,22
Q13231	CHIT1_HUMAN Chitotriosidase-1	13	13	4,75E-01	1,95	3,62E-01	0,71	2,64E-01	2,62	5,09E-04	121,60	7,32E-04	1,00	1,62E-01	0,21
Q06323	PSME1_HUMAN Proteasome activator complex subunit 1	6	6	5,17E-04	0,04	5,81E-01	0,78	4,84E-01	1,92	5,85E-06	45,39	1,19E-01	0,98	6,31E-02	0,21

P61163	ACTZ_HUMAN Alpha-centractin	7	5	5,85E-01	0,41	2,98E-01	0,66	1,87E-01	0,19	3,21E-02	22,64	4,33E-01	0,81	1,91E-01	0,21
P13995	MTDC_HUMAN Bifunctional methylenetetrahydrofolate dehydrogenase/cyclohydrolase , mitochondrial	3	2	1,98E-01	0,10	8,06E-01	1,11	4,97E-02	12,01	2,76E-05	71,68	4,08E-02	0,99	1,49E-01	0,21
Q07157	ZO1_HUMAN Tight junction protein Z	4	4	6,69E-01	0,87	3,61E-01	0,77	1,00E+00	1,00	1,00E+00	1,00	1,00E+00	0,50	3,03E-04	0,21
Q13492	PICAL_HUMAN Phosphatidylinositol-binding clathrin assembly protein	8	8	2,67E-01	0,13	5,77E-01	1,14	7,82E-01	0,58	1,36E-01	9,78	8,14E-01	0,59	3,96E-02	0,21
P62249	RS16_HUMAN 40S ribosomal protein S16	7	7	3,36E-01	0,23	4,59E-01	1,28	4,51E-02	2,64	1,35E-01	6,75	1,71E-03	1,00	4,27E-02	0,21
Q96I24	FUBP3_HUMAN Far upstream element-binding protein 3	4	4	2,60E-01	0,42	9,75E-01	1,03	1,00E+00	1,00	1,00E+00	1,00	4,74E-02	0,99	2,66E-01	0,21
P16278	BGAL_HUMAN Beta-galactosidase	8	8	1,81E-01	0,18	7,11E-01	0,86	1,99E-01	5,51	4,85E-03	15,40	5,81E-02	0,99	1,37E-01	0,21
Q86Y82	STX12_HUMAN Syntaxin-12	4	4	4,20E-01	2,38	5,56E-01	0,81	1,00E+00	1,00	1,20E-04	23,08	7,71E-01	0,61	2,70E-01	0,21
P15144	AMPN_HUMAN Aminopeptidase N	7	7	3,92E-01	0,51	5,14E-01	0,61	1,00E+00	1,00	3,19E-07	43,21	8,72E-02	0,98	4,34E-02	0,20
P16435	NCPR_HUMAN NADPH--cytochrome P450 reductase	3	3	6,01E-01	0,51	5,72E-01	0,75	5,57E-01	0,55	1,12E-01	8,53	1,00E+00	0,50	1,16E-02	0,20
Q9Y6C2	EMIL1_HUMAN EMILIN-1	12	12	3,45E-01	0,34	6,11E-01	1,73	8,01E-01	0,81	6,04E-02	1,61	1,36E-02	1,00	2,89E-01	0,20
P63027	VAMP2_HUMAN Vesicle-associated membrane protein 2	4	1	1,61E-01	0,48	8,77E-01	0,87	1,00E+00	1,00	1,28E-01	1,68	4,07E-02	0,99	3,12E-01	0,20
Q14914	PTGR1_HUMAN Prostaglandin reductase 1	4	4	2,27E-01	0,48	9,82E-01	1,02	8,91E-01	0,89	1,00E+00	1,00	7,25E-02	0,98	4,20E-02	0,20
P83731	RL24_HUMAN 60S ribosomal protein L24	4	4	9,03E-01	1,18	2,90E-01	1,43	9,67E-01	1,03	2,39E-03	6,40	7,65E-03	1,00	3,18E-02	0,19

O94979	SC31A_HUMAN Protein transport protein Sec31A	16	16	6,58E-01	0,58	4,91E-01	0,79	3,86E-01	0,42	1,54E-02	11,18	7,10E-01	0,64	5,58E-02	0,19
Q13263	TIF1B_HUMAN Transcription intermediary factor 1-beta	12	12	2,61E-01	0,14	9,18E-01	0,95	7,65E-01	0,79	1,25E-01	7,35	1,88E-01	0,95	1,83E-01	0,19
Q9UPN3	MACF1_HUMAN Microtubule-actin cross-linking factor 1, isoforms 1/2/3/5	25	24	3,64E-01	0,41	1,18E-01	0,30	5,00E-01	0,67	7,18E-01	1,70	4,25E-01	0,81	4,88E-02	0,19
O94925	GLSK_HUMAN Glutaminase kidney isoform, mitochondrial	17	17	2,10E-01	0,39	1,23E-02	0,50	7,42E-01	1,17	7,32E-01	1,28	2,38E-01	0,93	2,97E-02	0,19
Q16531	DDB1_HUMAN DNA damage-binding protein 1	5	5	4,18E-01	0,21	2,34E-01	1,64	1,00E+00	1,00	1,73E-06	112,32	2,94E-01	0,90	6,78E-02	0,19
Q07960	RHG01_HUMAN Rho GTPase-activating protein 1	16	14	2,51E-02	0,02	4,48E-01	0,73	6,86E-01	0,58	1,54E-01	3,36	8,80E-04	1,00	2,15E-01	0,19
Q8N766	EMC1_HUMAN ER membrane protein complex subunit 1	4	4	7,14E-01	1,47	6,92E-01	0,90	4,28E-01	1,70	9,34E-03	6,38	4,76E-01	0,78	9,39E-02	0,19
P48739	PIPNB_HUMAN Phosphatidylinositol transfer protein beta isoform	2	1	1,00E+00	1,00	4,42E-01	0,68	1,00E+00	1,00	3,51E-02	2,20	4,71E-02	0,99	1,93E-02	0,19
P55786	PSA_HUMAN Puromycin-sensitive aminopeptidase	16	16	3,97E-01	0,28	4,27E-01	0,38	7,78E-01	1,19	7,67E-04	16,89	1,42E-02	1,00	1,56E-01	0,18
P36542	ATPG_HUMAN ATP synthase subunit gamma, mitochondrial	6	6	6,60E-02	7,96	3,26E-01	0,57	7,88E-01	0,74	4,04E-02	21,53	1,40E-01	0,97	9,92E-02	0,17
P22087	FBRL_HUMAN rRNA 2-O-methyltransferase fibrillarin	2	2	3,14E-01	0,56	6,10E-01	0,73	1,00E+00	1,00	1,00E+00	1,00	4,98E-02	0,99	9,49E-04	0,17
P55290	CAD13_HUMAN Cadherin-13	5	4	4,66E-02	0,10	2,31E-02	0,47	6,53E-01	0,52	8,16E-01	1,07	9,58E-05	1,00	3,54E-02	0,17
O75367	H2AY_HUMAN Core histone macro-H2A.1	4	4	2,90E-01	0,30	7,10E-01	1,83	1,00E+00	1,00	2,49E-04	59,73	5,87E-02	0,99	2,47E-01	0,17

O15067	PUR4_HUMAN Phosphoribosylformylglycinamidine synthase	2	2	1,24E-01	21,01	7,01E-01	2,01	6,04E-01	3,69	9,70E-01	0,95	1,00E+00	0,50	2,28E-01	0,17
O75891	AL1L1_HUMAN Cytosolic 10-formyltetrahydrofolate dehydrogenase	2	2	1,75E-01	0,17	1,22E-01	0,68	1,00E+00	1,00	1,00E+00	1,00	3,33E-02	0,99	3,51E-02	0,17
Q13418	ILK_HUMAN Integrin-linked protein kinase	6	6	3,13E-02	0,08	7,64E-01	1,22	1,00E+00	1,00	1,17E-01	2,65	1,31E-01	0,97	1,39E-01	0,16
Q13310	PABP4_HUMAN Polyadenylate-binding protein 4	14	5	2,68E-01	0,28	4,04E-01	0,50	1,00E+00	1,00	9,12E-02	3,35	6,72E-02	0,99	4,32E-02	0,16
P61006	RAB8A_HUMAN Ras-related protein Rab-8A	3	2	9,75E-01	1,02	1,05E-01	0,40	1,00E+00	1,00	3,91E-02	8,08	1,72E-01	0,96	7,33E-02	0,16
Q86UE4	LYRIC_HUMAN Protein LYRIC	6	6	5,52E-01	0,43	2,92E-01	0,77	6,56E-01	0,59	3,78E-02	13,68	4,60E-02	0,99	6,60E-02	0,16
P12111	CO6A3_HUMAN Collagen alpha-3(VI) chain OS	23	23	5,95E-02	0,06	3,44E-01	0,59	7,04E-01	0,67	1,74E-01	2,47	1,01E-01	0,98	4,97E-02	0,16
Q92974	ARHG2_HUMAN Rho guanine nucleotide exchange factor 2	2	2	5,90E-01	0,77	3,02E-01	0,24	1,00E+00	1,00	1,00E+00	1,00	1,99E-02	0,99	1,02E-02	0,16
Q9Y617	SERC_HUMAN Phosphoserine aminotransferase	20	20	4,80E-02	0,03	1,07E-04	0,25	6,75E-01	1,51	8,42E-01	0,82	5,91E-03	1,00	2,01E-03	0,15
P11233	RALA_HUMAN Ras-related protein Ral-A	3	2	2,32E-01	0,42	4,21E-01	1,21	1,00E+00	1,00	1,53E-01	5,89	2,27E-02	0,99	1,61E-02	0,15
P13073	COX41_HUMAN Cytochrome c oxidase subunit 4 isoform 1, mitochondrial	4	4	4,08E-01	0,25	6,87E-01	0,84	2,30E-02	2,94	1,84E-06	112,47	9,46E-02	0,98	1,27E-02	0,15
P09493	TPM1_HUMAN Tropomyosin alpha-1 chain	31	12	3,08E-01	0,24	2,59E-02	0,64	4,54E-02	5,54	6,16E-01	0,70	4,80E-02	0,99	1,53E-04	0,15
Q9H2D6	TARA_HUMAN TRIO and F-actin-binding protein	4	4	5,60E-01	0,76	4,48E-01	0,45	3,57E-01	0,44	1,00E+00	1,00	2,06E-02	0,99	1,18E-02	0,15

Q12884	SEPR_HUMAN Prolyl endopeptidase FAP	7	7	6,48E-01	1,83	2,61E-02	0,37	3,77E-01	3,23	4,45E-01	0,51	2,17E-01	0,94	4,88E-02	0,15
P49821	NDUV1_HUMAN NADH dehydrogenase [ubiquinone] flavoprotein 1, mitochondrial	6	6	5,67E-01	0,55	1,27E-01	0,40	8,78E-01	0,82	8,47E-01	1,27	2,39E-01	0,93	1,51E-02	0,14
P31150	GDIA_HUMAN Rab GDP dissociation inhibitor alpha	17	9	6,63E-01	0,50	2,73E-01	0,38	3,84E-01	3,63	3,82E-02	9,07	1,20E-02	1,00	4,33E-02	0,14
Q9BY44	EIF2A_HUMAN Eukaryotic translation initiation factor 2A	3	3	5,68E-01	0,56	9,29E-01	1,04	9,95E-01	1,00	1,00E+00	1,00	4,02E-02	0,99	8,99E-03	0,14
P17980	PRS6A_HUMAN 26S proteasome regulatory subunit 6A	10	10	2,52E-01	0,14	2,24E-01	0,57	1,38E-01	0,18	9,59E-02	2,09	4,37E-01	0,80	5,90E-03	0,14
Q9Y3Z3	SAMH1_HUMAN Deoxynucleoside triphosphate triphosphohydrolase SAMHD1	11	11	2,54E-01	7,32	1,89E-01	2,29	9,77E-01	0,95	1,02E-03	134,00	9,34E-01	0,53	4,57E-02	0,14
P19971	TYPH_HUMAN Thymidine phosphorylase	9	9	1,24E-01	9,40	6,20E-01	1,49	9,72E-01	1,06	6,14E-05	260,84	5,29E-01	0,74	1,56E-01	0,14
P31937	3HIDH_HUMAN 3-hydroxyisobutyrate dehydrogenase, mitochondrial	3	3	3,18E-01	0,23	9,45E-01	1,03	7,96E-01	0,77	7,83E-05	72,01	1,99E-01	0,95	9,86E-02	0,14
Q0ZGT2	NEXN_HUMAN Nexilin	6	6	5,21E-01	2,14	9,60E-02	0,22	6,18E-01	1,28	5,42E-01	0,50	1,09E-01	0,98	7,34E-03	0,13
Q9H4M9	EHD1_HUMAN EH domain-containing protein 1	12	10	2,99E-01	0,20	5,19E-01	0,73	2,20E-03	0,07	5,59E-01	1,99	1,22E-03	1,00	9,78E-03	0,13
P17858	PFKAL_HUMAN ATP-dependent 6-phosphofructokinase, liver type	5	4	6,49E-01	0,72	1,87E-01	1,61	7,15E-01	0,68	3,59E-02	7,23	1,00E+00	0,50	2,57E-02	0,13
Q6NUK1	SCMC1_HUMAN Calcium-binding mitochondrial carrier protein SCaMC-1	5	5	1,28E-01	0,23	6,97E-01	0,71	1,00E+00	1,00	5,79E-06	86,49	2,09E-01	0,94	6,72E-02	0,13

Q02818	NUCB1_HUMAN Nucleobindin-1	9	9	3,11E-02	0,05	2,47E-01	0,64	7,36E-01	0,71	5,30E-01	0,33	1,28E-01	0,97	9,86E-03	0,13
P54709	AT1B3_HUMAN Sodium/potassium-transporting ATPase subunit beta-3	3	3	9,60E-01	0,97	4,59E-01	0,49	9,14E-01	1,07	1,45E-01	3,52	1,00E+00	0,50	2,92E-02	0,13
P40261	NNMT_HUMAN Nicotinamide N-methyltransferase	8	8	2,28E-01	0,10	1,79E-02	0,55	7,02E-01	0,76	8,91E-02	1,73	5,65E-01	0,72	3,69E-02	0,12
Q13162	PRDX4_HUMAN Peroxiredoxin-4	10	8	4,12E-01	0,29	6,57E-01	0,69	4,81E-01	1,44	1,42E-02	18,83	6,58E-02	0,99	6,56E-02	0,12
Q13510	ASAH1_HUMAN Acid ceramidase	7	7	4,28E-03	0,13	3,84E-01	0,47	2,26E-01	1,97	9,81E-05	343,09	2,12E-01	0,94	8,32E-04	0,12
O00629	IMA3_HUMAN Importin subunit alpha-3	7	5	7,87E-01	0,62	4,15E-02	0,74	5,00E-01	0,33	1,63E-02	20,19	4,73E-01	0,78	7,68E-02	0,12
P30085	KCY_HUMAN UMP-CMP kinase	10	10	2,08E-01	0,15	4,36E-02	0,31	2,54E-01	0,16	6,84E-02	4,05	7,17E-01	0,63	1,04E-01	0,12
P63241	IF5A1_HUMAN Eukaryotic translation initiation factor 5A-1	6	6	2,24E-01	0,11	1,05E-01	0,55	1,52E-01	16,39	4,91E-02	0,04	1,71E-02	0,99	2,49E-01	0,12
P19878	NCF2_HUMAN Neutrophil cytosol factor 2	5	5	8,70E-01	0,89	6,51E-01	0,67	5,71E-01	0,56	1,15E-03	61,12	1,00E+00	0,50	3,21E-02	0,12
P61803	DAD1_HUMAN Dolichyl-diphosphooligosaccharide--protein glycosyltransferase subunit DAD1	3	3	1,65E-01	0,09	5,59E-01	1,13	6,75E-01	1,28	8,25E-07	121,00	1,15E-01	0,98	8,11E-02	0,12
P00966	ASSY_HUMAN Argininosuccinate synthase	3	3	2,19E-01	0,50	5,79E-01	0,59	3,13E-01	2,73	1,35E-01	2,09	1,19E-01	0,98	1,36E-02	0,11
P52943	CRIP2_HUMAN Cysteine-rich protein 2	2	2	1,40E-01	0,27	6,33E-02	0,19	1,00E+00	1,00	1,00E+00	1,00	6,52E-02	0,99	4,65E-02	0,11
Q07954	LRP1_HUMAN Prolow-density lipoprotein receptor-related protein 1	29	29	1,13E-02	0,08	7,59E-01	0,76	6,42E-01	0,62	9,33E-01	1,04	2,91E-01	0,90	1,26E-01	0,11

P02649	APOE_HUMAN Apolipoprotein E	9	6	7,38E-01	0,82	5,99E-01	0,77	8,47E-01	1,25	3,95E-04	129,93	6,37E-02	0,99	5,29E-04	0,11
Q08378	GOGA3_HUMAN Golgin subfamily A member 3	4	4	1,37E-01	0,36	1,02E-01	0,32	1,00E+00	1,00	7,26E-02	2,11	3,75E-02	0,99	2,17E-02	0,10
Q16822	PCKGM_HUMAN Phosphoenolpyruvate carboxykinase [GTP], mitochondrial	9	9	2,45E-01	0,24	6,59E-02	0,21	1,00E+00	1,00	5,59E-04	40,85	1,38E-01	0,97	2,01E-02	0,10
P08134	RHOC_HUAMN Rho-related GTP-binding protein RhoC	7	7	2,28E-02	0,07	8,46E-02	0,19	3,14E-01	0,29	5,29E-03	16,00	1,52E-02	1,00	6,98E-02	0,10
Q9UBI6	GBG12_HUMAN Guanine nucleotide-binding protein G(I)/G(S)/G(O) subunit gamma-12	3	3	2,09E-01	0,14	4,25E-01	0,64	1,00E+00	1,00	1,00E+00	1,00	1,00E+00	0,50	4,37E-02	0,10
Q96IZ0	PAWR_HUMAN PRKC apoptosis WT1 regulator protein	3	3	1,74E-01	0,26	3,52E-01	0,81	9,27E-02	2,14	1,00E+00	1,00	2,10E-03	1,00	9,32E-03	0,09
Q6IAA8	LTOR1_HUMAN Ragulator complex protein LAMT	3	3	1,76E-01	0,37	5,67E-01	0,63	1,00E+00	1,00	1,22E-02	21,91	6,77E-03	1,00	4,08E-02	0,09
O14773	TPP1_HUMAN Tripeptidyl-peptidase 1	6	6	8,91E-01	1,17	2,58E-01	0,50	1,58E-01	0,11	7,28E-03	36,39	5,97E-01	0,70	1,35E-02	0,09
O95816	BAG2_HUMAN BAG family molecular chaperone regulator 2	3	3	3,79E-01	0,40	9,28E-01	0,90	7,58E-01	1,17	1,00E+00	1,00	3,53E-02	0,99	2,00E-02	0,09
O76094	SRP72_HUMAN Signal recognition particle subunit SRP72	2	2	1,48E-01	0,22	6,47E-01	0,67	1,00E+00	1,00	1,00E+00	1,00	4,32E-03	1,00	3,20E-02	0,08
Q27J81	INF2_HUMAN Inverted formin-2	5	5	3,81E-02	17,84	9,86E-01	1,00	4,62E-01	4,13	5,90E-01	0,53	1,17E-01	0,98	6,34E-02	0,08
Q92572	AP3S1_HUMAN AP-3 complex subunit sigma-1	2	2	4,25E-02	0,06	2,34E-01	0,84	1,00E+00	1,00	1,00E+00	1,00	1,29E-01	0,97	8,03E-05	0,08
O60716	CTND1_HUMAN Catenin delta-1	5	5	1,51E-01	0,19	2,61E-01	0,41	1,00E+00	1,00	2,09E-01	2,94	9,32E-02	0,98	3,60E-05	0,07

P25325	THTM_HUMAN 3-mercaptopyruvate sulfurtransferase	3	3	5,62E-01	1,89	7,19E-01	0,72	1,00E+00	1,00	1,00E+00	1,00	1,00E+00	0,50	6,49E-04	0,07
Q3SY69	AL1L2_HUMAN Mitochondrial 10-formyltetrahydrofolate dehydrogenase	9	9	2,44E-01	0,23	2,11E-02	0,24	1,00E+00	1,00	3,69E-01	2,92	4,63E-01	0,79	1,83E-02	0,07
Q9HC35	EMAL4_HUMAN Echinoderm microtubule-associated protein-like 4	4	4	5,46E-01	0,43	6,79E-03	0,47	8,79E-01	1,20	3,06E-01	3,67	7,04E-01	0,64	4,43E-02	0,07
Q92890	UFD1_HUMAN Ubiquitin recognition factor in ER-associated degradation protein 1	2	2	7,87E-01	0,60	2,51E-01	0,66	7,64E-01	1,72	6,30E-01	0,56	1,00E+00	0,50	1,51E-03	0,07
O00622	CCN1_HUMAN CCN family member 1	4	4	2,09E-01	0,42	9,55E-02	0,14	4,17E-01	0,44	6,96E-02	4,09	3,45E-03	1,00	5,55E-03	0,06
P16989	YBOX3_HUMAN Y-box-binding protein 3	7	3	1,00E+00	1,00	1,46E-01	0,31	1,00E+00	1,00	1,00E+00	1,00	1,00E+00	0,50	3,42E-04	0,06
P08243	ASNS_HUMAN Asparagine synthetase [glutamine-hydrolyzing]	4	4	4,20E-02	0,07	1,58E-01	0,36	1,00E+00	1,00	1,00E+00	1,00	6,09E-02	0,99	7,01E-06	0,06
Q9UH99	SUN2_HUMAN SUN domain-containing protein 2	4	4	1,00E+00	1,00	7,10E-01	0,68	1,00E+00	1,00	6,07E-02	2,43	1,07E-01	0,98	8,92E-04	0,06
Q9ULC3	RAB23_HUMAN Ras-related protein Rab-23	3	3	8,56E-01	1,38	1,02E-01	1,52	7,16E-01	0,41	8,46E-01	1,54	1,00E+00	0,50	3,23E-02	0,05
Q13557	KCC2D_HUMAN Calcium/calmodulin-dependent protein kinase type II subunit delta	4	4	9,00E-01	1,22	1,96E-01	0,35	1,24E-01	2,13	7,53E-02	0,16	1,68E-02	0,99	4,48E-02	0,05
P61081	UBC12_HUMAN NEDD8-conjugating enzyme Ubc12	3	3	7,21E-01	1,16	3,41E-01	0,27	1,00E+00	1,00	1,00E+00	1,00	6,14E-03	1,00	1,81E-03	0,04
P12109	CO6A1_HUMAN Collagen alpha-1(VI) chain	5	5	9,05E-01	0,88	7,45E-01	0,73	6,96E-01	0,61	8,62E-01	0,79	1,43E-02	1,00	1,13E-03	0,04

P29992	GNA11_HUMAN Guanine nucleotide-binding protein subunit alpha-11	2	2	2,47E-01	0,30	4,08E-01	0,84	5,60E-01	0,44	1,00E+00	1,00	1,68E-01	0,96	1,72E-03	0,04
P13611	CSPG2_HUMAN Versican core protein	9	8	9,68E-01	0,93	1,38E-01	0,41	8,44E-01	0,70	4,84E-01	3,12	3,38E-02	0,99	1,71E-02	0,04
O00151	PDLI1_HUMAN PDZ and LIM domain protein 1	3	3	4,81E-01	0,76	5,31E-02	0,08	1,28E-01	1,83	1,00E+00	1,00	1,36E-01	0,97	7,63E-04	0,04
P68402	PA1B2_HUMAN Platelet-activating factor acetylhydrolase IB subunit beta	4	3	2,22E-01	0,14	1,34E-01	4,44	1,00E+00	1,00	1,00E+00	1,00	1,00E+00	0,50	5,37E-09	0,03
P98095	FBLN2_HUMAN Fibulin-2	11	11	5,33E-01	0,32	9,16E-02	0,13	4,95E-01	0,36	1,12E-01	5,19	7,83E-02	0,98	1,09E-03	0,03
O95202	LETM1_HUMAN Mitochondrial proton/calcium exchanger protein	6	6	4,56E-01	0,42	9,14E-01	1,13	6,62E-01	1,17	1,57E-01	2,73	8,40E-01	0,57	7,98E-05	0,03
