# Supplementary material for: Proteomic Analysis of Mesenchymal Stem Cells and Monocyte Co-Cultures Exposed to a Bioactive Silica-Based Sol–Gel Coating
Source: ACS Biomater Sci Eng. 2023 May 19;9(6):3306–19. doi: 10.1021/acsbiomaterials.3c00254 (PMC10265575; doi:10.1021/acsbiomaterials.3c00254)
Supplement: Supplementary file 2 — ab3c00254_si_002.pdf [file ab3c00254_si_002.pdf]

Proteomic analysis of mesenchymal stem cells and monocytes co-cultures exposed to a bioactive silica-based sol-gel coating

Andreia Cerqueira[1*], Francisco Romero-Gavilán[1], Heike Helmholz[2], Mikel Azkargorta[3], Félix Elortza[3], Mariló Gurruchaga[4], Isabel Goñi[4], Regine Willumeit-Römer[2], Julio Suay[1]

[1]Department of Industrial Systems Engineering and Design, Universitat Jaume I, Av. Vicent Sos Baynat s/n, 12071 Castellón de la Plana, Spain

[2]Helmholtz-Zentrum Hereon Institute of Metallic Biomaterials, Max-Planck-St.1, Geesthacht D-21502, Germany

[3]Proteomics Platform, CIC bioGUNE, Basque Research and Technology Alliance (BRTA), CIBERehd, Bizkaia Science and Technology Park, 48160 Derio, Spain

[4]Departament of Science and Technology of Polymers, University of the Basque Country, P. M. de Lardizábal, 3, 20018 San Sebastián, Spain

*Corresponding author: Andreia Cerqueira. E-mail: lages@uji.es

Supplementary Information: 7 pages

Table S2. First 150 proteins differentially expressed in co-culture systems exposed to MT in relation to Ti after 7 days of assay.

Table S2. First 150 proteins differentially expressed in co-culture systems exposed to MT in relation to Ti after 7 days of assay and used for STRING analysis. Proteins with ANOVA $p < 0.05$ (yellow) and a ratio higher than 1.5 in either direction were considered as significantly different. Upregulated proteins are marked in red and downregulated proteins appear in green.

Accession	Description	*p* value	Ratio
O94808	GFPT2_HUMAN Glutamine--fructose-6-phosphate aminotransferase [isomerizing] 2	5,96E-03	89,56
Q93077	H2A1C_HUMAN Histone H2A type 1-C	7,35E-03	19,17
Q9Y3I0	RTCB_HUMAN RNA-splicing ligase RtcB homolog	3,55E-02	17,05
P51648	AL3A2_HUMAN Aldehyde dehydrogenase family 3 member A2	3,95E-03	6,47
P19784	CSK22_HUMAN Casein kinase II subunit alpha	3,63E-03	4,05
P62277	RS13_HUMAN 40S ribosomal protein S13	2,00E-02	2,23
Q99961	SH3G1_HUMAN Endophilin-A2	7,40E-03	0,61
Q9H2D6	TARA_HUMAN TRIO and F-actin-binding protein	2,06E-02	0,60
A8MVU1	NCF1C_HUMAN Putative neutrophil cytosol factor 1C	4,50E-02	0,59
P00492	HPRT_HUMAN Hypoxanthine-guanine phosphoribosyltransferase	4,01E-02	0,57
P50897	PPT1_HUMAN Palmitoyl-protein thioesterase 1	4,50E-02	0,55
Q96CX2	KCD12_HUMAN BTB/POZ domain-containing protein	8,74E-03	0,55
Q99733	NP1L4_HUMAN Nucleosome assembly protein 1-like 4	1,73E-02	0,54
P26447	S10A4_HUMAN Protein S100-A4	1,39E-02	0,53
Q9BY44	EIF2A_HUMAN Eukaryotic translation initiation factor 2A	4,02E-02	0,53
P04839	CY24B_HUMAN Cytochrome b-245 heavy chain	1,68E-02	0,51
P39748	FEN1_HUMAN Flap endonuclease 1	2,00E-03	0,50
P25391	LAMA1_HUMAN Laminin subunit alpha-1	1,68E-02	0,50
Q7L2H7	EIF3M_HUMAN Eukaryotic translation initiation factor 3 subunit M	2,19E-02	0,50
Q9Y5B9	SP16H_HUMAN FACT complex subunit SPT16	4,15E-02	0,50
P12235	ADT1_HUMAN ADP/ATP translocase 1	1,90E-02	0,50
P00738	HPT_HUMAN Haptoglobin	1,59E-02	0,49
Q16401	PSMD5_HUMAN 26S proteasome non-ATPase regulatory subunit 5	3,45E-02	0,49

P48594	SPB4_HUMAN Serpin B4	1,92E-02	0,49
Q9UBB4	ATX10_HUMAN Ataxin-10	2,67E-02	0,48
Q9Y2D5	AKAP2_HUMAN A-kinase anchor protein 2	4,71E-02	0,48
O14908	GIPC1_HUMAN PDZ domain-containing protein GIPC1	7,82E-03	0,48
P29144	TPP2_HUMAN Tripeptidyl-peptidase 2	1,34E-02	0,48
Q86TX2	ACOT1_HUMAN Acyl-coenzyme A	1,59E-02	0,47
P00918	CAH2_HUMAN Carbonic anhydrase 2	3,45E-02	0,47
P35555	FBN1_HUMAN Fibrillin-1	2,30E-02	0,46
Q07955	SRSF1_HUMAN Serine/arginine-rich splicing factor 1	1,76E-02	0,46
O75891	AL1L1_HUMAN Cytosolic 10-formyltetrahydrofolate dehydrogenase	3,33E-02	0,46
Q9UQ35	SRRM2_HUMAN Serine/arginine repetitive matrix protein 2	7,38E-03	0,46
Q06124	PTN11_HUMAN Tyrosine-protein phosphatase non-receptor type 11	5,40E-03	0,46
O43765	SGTA_HUMAN Small glutamine-rich tetratricopeptide repeat-containing protein alpha	4,38E-02	0,45
Q8N8S7	ENAH_HUMAN Protein enabled homolog	3,82E-02	0,44
P43304	GPDM_HUMAN Glycerol-3-phosphate dehydrogenase, mitochondrial	1,52E-02	0,44
O14929	HAT1_HUMAN Histone acetyltransferase type B catalytic subunit	8,23E-03	0,44
Q8NCA5	FA98A_HUMAN Protein FAM98A	4,48E-02	0,44
P83111	LACTB_HUMAN Serine beta-lactamase-like protein LACTB, mitochondrial	4,42E-02	0,43
Q14683	SMC1A_HUMAN Structural maintenance of chromosomes protein 1A	2,74E-02	0,43
Q9Y6C2	EMIL1_HUMAN EMILIN-1	1,36E-02	0,43
O75643	U520_HUMAN U5 small nuclear ribonucleoprotein 200 kDa helicase	1,98E-02	0,43
O43598	DNPH1_HUMAN 2-deoxynucleoside 5-phosphate N-hydrolase 1	4,12E-02	0,42
P13686	PPA5_HUMAN Tartrate-resistant acid phosphatase type 5	1,10E-02	0,42
P42765	THIM_HUMAN 3-ketoacyl-CoA thiolase, mitochondrial	4,31E-03	0,42
P10909	CLUS_HUMAN Clusterin	2,17E-03	0,42
O75489	NDUS3_HUMAN NADH dehydrogenase [ubiquinone] iron-sulfur protein 3, mitochondrial	3,17E-02	0,42
P49006	MRP_HUMAN MARCKS-related protein	2,91E-02	0,42
Q8NC56	LEMD2_HUMAN LEM domain-containing protein 2	1,32E-04	0,41

Q9Y2W1	TR150_HUMAN Thyroid hormone receptor-associated protein 3	4,28E-02	0,41
P47929	LEG7_HUMAN Galectin-7	7,05E-03	0,40
Q9NUQ9	CYRIB_HUMAN CYFIP-related Rac1 interactor B	1,68E-02	0,40
Q14694	UBP10_HUMAN Ubiquitin carboxyl-terminal hydrolase 10	4,89E-02	0,39
Q15393	SF3B3_HUMAN Splicing factor 3B subunit 3	2,07E-02	0,39
Q15056	IF4H_HUMAN Eukaryotic translation initiation factor 4H	2,38E-02	0,39
P15374	UCHL3_HUMAN Ubiquitin carboxyl-terminal hydrolase isozyme L3	1,86E-02	0,39
P29508	SPB3_HUMAN Serpin B3	2,56E-02	0,39
Q9NSE4	SYIM_HUMAN Isoleucine--tRNA ligase, mitochondrial	1,29E-02	0,38
Q16647	PTGIS_HUMAN Prostacyclin synthase	1,02E-02	0,38
O00560	SDCB1_HUMAN Syntenin-1	1,79E-02	0,38
P10253	LYAG_HUMAN Lysosomal alpha-glucosidase	2,47E-02	0,38
Q96RP9	EFGM_HUMAN Elongation factor G, mitochondrial	2,25E-03	0,38
O43854	EDIL3_HUMAN EGF-like repeat and discoidin I-like domain-containing protein 3	5,59E-03	0,37
Q9Y263	PLAP_HUMAN Phospholipase A-2-activating protein	2,72E-03	0,37
Q92905	CSN5_HUMAN COP9 signalosome complex subunit 5	5,43E-03	0,37
P43121	MUC18_HUMAN Cell surface glycoprotein MUC18	1,01E-02	0,37
P46977	STT3A_HUMAN Dolichyl-diphosphooligosaccharide--protein glycosyltransferase subunit STT3A	2,26E-02	0,37
P61086	UBE2K_HUMAN Ubiquitin-conjugating enzyme E2 K	2,10E-02	0,36
Q92974	ARHG2_HUMAN Rho guanine nucleotide exchange factor 2	1,99E-02	0,36
P13473	LAMP2_HUMAN Lysosome-associated membrane glycoprotein 2	3,51E-02	0,36
Q9NZ32	ARP10_HUMAN Actin-related protein 10	2,18E-02	0,36
P18031	PTN1_HUMAN Tyrosine-protein phosphatase non-receptor type 1	2,67E-02	0,36
Q9NUJ1	ABHDA_HUMAN Mycophenolic acid acyl-glucuronide esterase, mitochondrial	4,13E-02	0,36
P30046	DOPD_HUMAN D-dopachrome decarboxylase	4,02E-02	0,36
P30533	AMRP_HUMAN Alpha-2-macroglobulin receptor-associated protein	4,76E-02	0,36
Q9H2U2	IPYR2_HUMAN Inorganic pyrophosphatase 2, mitochondrial	2,96E-02	0,36
P12931	SRC_HUMAN Proto-oncogene tyrosine-protein kinase Src	6,08E-03	0,35

P09038	FGF2_HUMAN Fibroblast growth factor 2	4,62E-03	0,35
P22102	PUR2_HUMAN Trifunctional purine biosynthetic protein adenosine-3	1,53E-02	0,35
P14780	MMP9_HUMAN Matrix metalloproteinase-9	2,27E-03	0,35
Q9BXJ9	NAA15_HUMAN N-alpha-acetyltransferase 15, NatA auxiliary subunit	3,88E-05	0,35
P61201	CSN2_HUMAN COP9 signalosome complex subunit 2	4,95E-02	0,35
Q15459	SF3A1_HUMAN Splicing factor 3A subunit 1	4,16E-02	0,34
Q6IAA8	LTOR1_HUMAN Ragulator complex protein LAMT	6,77E-03	0,34
P01834	IGKC_HUMAN Immunoglobulin kappa constant	4,15E-02	0,33
Q96BM9	ARL8A_HUMAN ADP-ribosylation factor-like protein 8A	3,20E-02	0,33
P68363	TBA1B_HUMAN Tubulin alpha-1B chain	2,96E-03	0,33
Q9NVS9	PNPO_HUMAN Pyridoxine-5-phosphate oxidase	2,10E-03	0,33
Q53GG5	PDLI3_HUMAN PDZ and LIM domain protein 3	2,44E-02	0,33
Q96I24	FUBP3_HUMAN Far upstream element-binding protein 3	4,74E-02	0,32
P19525	E2AK2_HUMAN Interferon-induced, double-stranded RNA-activated protein kinase	1,66E-02	0,32
Q14112	NID2_HUMAN Nidogen-2	2,03E-02	0,32
P63027	VAMP2_HUMAN Vesicle-associated membrane protein 2	4,07E-02	0,32
P33897	ABCD1_HUMAN ATP-binding cassette sub-family D member 1	6,50E-03	0,31
Q08378	GOGA3_HUMAN Golgin subfamily A member 3	3,75E-02	0,31
Q8NBL1	PGLT1_HUMAN Protein	2,41E-02	0,31
Q15843	NEDD8_HUMAN NEDD8	1,92E-02	0,31
Q13636	RAB31_HUMAN Ras-related protein Rab-31	1,88E-02	0,31
P55795	HNRH2_HUMAN Heterogeneous nuclear ribonucleoprotein H2	2,55E-02	0,31
P15170	ERF3A_HUMAN Eukaryotic peptide chain release factor GTP-binding subunit ERF3A	4,96E-02	0,30
P04114	APOB_HUMAN Apolipoprotein B-100	4,98E-02	0,30
O75116	ROCK2_HUMAN Rho-associated protein kinase 2	1,39E-02	0,30
P17050	NAGAB_HUMAN Alpha-N-acetylgalactosaminidase	2,39E-03	0,29
Q9UHQ9	NB5R1_HUMAN NADH-cytochrome b5 reductase 1	4,01E-02	0,29
P23919	KTHY_HUMAN Thymidylate kinase	1,61E-02	0,29

P31949	S10AB_HUMAN Protein S100-A11	2,18E-02	0,29
P49419	AL7A1_HUMAN Alpha-aminoadipic semialdehyde dehydrogenase	3,29E-02	0,29
Q86V81	THOC4_HUMAN THO complex subunit 4	1,39E-02	0,29
Q12931	TRAP1_HUMAN Heat shock protein 75 kDa, mitochondrial	5,52E-03	0,28
P60709	ACTB_HUMAN Actin, cytoplasmic 1	2,82E-03	0,28
Q9NRV9	HEBP1_HUMAN Heme-binding protein 1	1,24E-02	0,28
P14618	KPYM_HUMAN Pyruvate kinase PKM	8,96E-03	0,28
P21964	COMT_HUMAN Catechol	2,30E-02	0,28
P26196	DDX6_HUMAN Probable ATP-dependent RNA helicase DDX6	4,23E-02	0,28
Q9HD20	AT131_HUMAN Manganese-transporting ATPase 13A1	1,46E-02	0,28
Q96FQ6	S10AG_HUMAN Protein S100-A16	4,51E-02	0,28
P12004	PCNA_HUMAN Proliferating cell nuclear antigen	3,53E-02	0,28
P22087	FBRL_HUMAN rRNA 2-O=-methyltransferase fibrillarin	4,98E-02	0,28
P29373	RABP2_HUMAN Cellular retinoic acid-binding protein 2	2,37E-02	0,27
Q9Y5S1	TRPV2_HUMAN Transient receptor potential cation channel subfamily V member 2	4,82E-02	0,27
O75886	STAM2_HUMAN Signal transducing adapter molecule 2	3,86E-02	0,27
Q14192	FHL2_HUMAN Four and a half LIM domains protein 2	4,30E-02	0,27
Q5JRX3	PREP_HUMAN Presequence protease, mitochondrial	2,96E-02	0,27
P11233	RALA_HUMAN Ras-related protein Ral-A	2,27E-02	0,27
Q7Z2K6	ERMP1_HUMAN Endoplasmic reticulum metallopeptidase 1	3,80E-02	0,27
P62937	PPIA_HUMAN Peptidyl-prolyl cis-trans isomerase A	1,07E-03	0,27
P29317	EPHA2_HUMAN Ephrin type-A receptor 2	1,29E-02	0,27
P36222	CH3L1_HUMAN Chitinase-3-like protein 1	3,78E-02	0,27
P21912	SDHB_HUMAN Succinate dehydrogenase [ubiquinone] iron-sulfur subunit, mitochondrial	2,29E-02	0,27
Q92520	FAM3C_HUMAN Protein FAM3C	3,91E-02	0,27
Q14344	GNA13_HUMAN Guanine nucleotide-binding protein subunit alpha-13	1,61E-02	0,27
P52888	THOP1_HUMAN Thimet oligopeptidase	3,41E-02	0,26
P31944	CASPE_HUMAN Caspase-14	1,60E-02	0,26

P35354	PGH2_HUMAN Prostaglandin G/H synthase 2	1,32E-02	0,26
O96008	TOM40_HUMAN Mitochondrial import receptor subunit T OM40 homolog	9,37E-03	0,26
Q13155	AIMP2_HUMAN Aminoacyl tRNA synthase complex-interacting multifunctional protein 2	4,47E-02	0,26
Q12905	ILF2_HUMAN Interleukin enhancer-binding factor 2	4,40E-02	0,26
P53007	TXTP_HUMAN Tricarboxylate transport protein, mitochondrial	3,51E-02	0,26
Q13126	MTAP_HUMAN S-methyl-5-thioadenosine phosphorylase	2,37E-02	0,26
Q9UBS4	DJB11_HUMAN DnaJ homolog subfamily B member 11	4,37E-02	0,25
P01857	IGHG1_HUMAN Immunoglobulin heavy constant gamma 1	2,56E-02	0,25
O95479	G6PE_HUMAN GDH/6PGL endoplasmic bifunctional protein	3,74E-02	0,25
P09874	PARP1_HUMAN Poly [ADP-ribose] polymerase 1	4,41E-02	0,25
P55899	FCGRN_HUMAN IgG receptor FcRn large subunit p51	4,17E-02	0,25
Q14677	EPN4_HUMAN Clathrin interactor 1	2,76E-03	0,24
P16070	CD44_HUMAN CD44 antigen	3,60E-03	0,24
P06737	PYGL_HUMAN Glycogen phosphorylase, liver form	1,31E-02	0,24
P54619	AAKG1_HUMAN 5-AMP-activated protein kinase subunit gamma-1	4,86E-02	0,24
