# Supplementary material for: Proteomic Analysis of Mesenchymal Stem Cells and Monocyte Co-Cultures Exposed to a Bioactive Silica-Based Sol–Gel Coating
Source: ACS Biomater Sci Eng. 2023 May 19;9(6):3306–19. doi: 10.1021/acsbiomaterials.3c00254 (PMC10265575; doi:10.1021/acsbiomaterials.3c00254)
Supplement: Supplementary file 3 — ab3c00254_si_003.pdf [file ab3c00254_si_003.pdf]

Proteomic analysis of mesenchymal stem cells and monocytes co-cultures exposed to a bioactive silica-based sol-gel coating

Andreia Cerqueira[1*], Francisco Romero-Gavilán[1], Heike Helmholz[2], Mikel Azkargorta[3], Félix Elortza[3], Mariló Gurruchaga[4], Isabel Goñi[4], Regine Willumeit-Römer[2], Julio Suay[1]

[1]Department of Industrial Systems Engineering and Design, Universitat Jaume I, Av. Vicent Sos Baynat s/n, 12071 Castellón de la Plana, Spain

[2]Helmholtz-Zentrum Hereon Institute of Metallic Biomaterials, Max-Planck-St.1, Geesthacht D-21502, Germany

[3]Proteomics Platform, CIC bioGUNE, Basque Research and Technology Alliance (BRTA), CIBERehd, Bizkaia Science and Technology Park, 48160 Derio, Spain

[4]Departament of Science and Technology of Polymers, University of the Basque Country, P. M. de Lardizábal, 3, 20018 San Sebastián, Spain

*Corresponding author: Andreia Cerqueira. E-mail: lages@uji.es

Supplementary Information: 7 pages

Table S3. First 150 proteins differentially expressed in co-culture systems exposed to MT in relation to Ti after 14 days of assay.

Table S3. First 150 proteins differentially expressed in co-culture systems exposed to MT in relation to Ti after 14 days of assay and used for STRING analysis. Proteins with ANOVA $p < 0.05$ (yellow) and a ratio higher than 1.5 in either direction were considered as significantly different. Upregulated proteins are marked in red and downregulated proteins appear in green.

Accession	Description	*p* value	Ratio
P17813	FABP5_HUMAN Fatty acid-binding protein 5	5,24E-03	12,89
P27482	SATT_HUMAN Neutral amino acid transporter A	3,62E-03	6,52
Q9NZB2	ATPMD_HUMAN ATP synthase membrane subunit DAPIT, mitochondrial	3,39E-02	5,37
Q14956	CALL5_HUMAN Calmodulin-like protein 5	5,04E-03	4,60
P36222	ALDH2_HUMAN Aldehyde dehydrogenase, mitochondrial	3,62E-03	3,40
P36405	CSK22_HUMAN Casein kinase II subunit alpha	1,07E-02	2,70
O60884	COTL1_HUMAN Coactosin-like protein	8,53E-03	2,39
O43488	PLOD2_HUMAN Procollagen-lysine,2-oxoglutarate 5-dioxygenase	4,58E-03	2,21
P62495	EIF3K_HUMAN Eukaryotic translation initiation factor 3 subunit K	5,02E-03	2,14
P51149	IDE_HUMAN Insulin-degrading enzyme	3,30E-02	2,07
Q08554	PLD3_HUMAN 5-3 exonuclease PLD3	4,74E-02	2,05
P28331	RL10A_HUMAN 60S ribosomal protein L10a	7,02E-03	2,04
O14818	PRDX2_HUMAN Peroxiredoxin-2	2,79E-02	1,64
P07900-2	TOM20_HUMAN Mitochondrial import receptor subunit TOM20 homolog	3,48E-03	1,63
O43237	ECI2_HUMAN Enoyl-CoA delta isomerase 2, mitochondrial	1,64E-03	1,59
Q16576	RL27_HUMAN 60S ribosomal protein L27	4,81E-02	0,66
Q9Y5S1	PGK1_HUMAN Phosphoglycerate kinase 1	1,06E-02	0,64
P43307	GANAB_HUMAN Neutral alpha-glucosidase AB	2,36E-02	0,64
P49588	SPTN1_HUMAN Spectrin alpha chain, non-erythrocytic 1	4,04E-02	0,64
P04083	HNRPU_HUMAN Heterogeneous nuclear ribonucleoprotein U	4,23E-02	0,63
P47897	CATZ_HUMAN Cathepsin Z	3,91E-02	0,61
P61201	RS11_HUMAN 40S ribosomal protein S11	1,81E-03	0,61

Q9H223	PTPA_HUMAN Serine/threonine-protein phosphatase 2A activator	4,57E-02	0,61
Q0VD83	PLEC_HUMAN Plectin	1,70E-02	0,61
P38646	CAN2_HUMAN Calpain-2 catalytic subunit	4,59E-02	0,59
P29317	YKT6_HUMAN Synaptobrevin homolog YKT6	4,27E-02	0,59
P46060	SEP11_HUMAN Septin-11	4,93E-02	0,58
P61026	GELS_HUMAN Gelsolin	1,02E-02	0,58
P56192	SERA_HUMAN D-3-phosphoglycerate dehydrogenase	4,56E-03	0,58
P06280	CO1A1_HUMAN Collagen alpha-1(I) chain	1,99E-02	0,57
P30084	IF2B1_HUMAN Insulin-like growth factor 2 mRNA-binding protein 1	4,33E-02	0,57
Q9H4M9	BAX_HUMAN Apoptosis regulator BAX	4,79E-02	0,56
Q8N1F7	RCN3_HUMAN Reticulocalbin-3	2,29E-03	0,56
P36542	ERP29_HUMAN Endoplasmic reticulum resident protein 29	2,12E-02	0,55
Q5TZA2	STX7_HUMAN Syntaxin-7	2,64E-02	0,55
Q9NUU7	P3H1_HUMAN Prolyl 3-hydroxylase 1	4,23E-03	0,54
P62081	GDIR1_HUMAN Rho GDP-dissociation inhibitor 1	2,91E-02	0,53
Q9UI30	ARPC2_HUMAN Actin-related protein 2/3 complex subunit 2	4,39E-02	0,53
P67870	CALD1_HUMAN Caldesmon	1,42E-02	0,52
Q9UNS2	S10A6_HUMAN Protein S100-A6	2,23E-02	0,51
Q7Z406	RAP1B_HUMAN Ras-related protein Rap-1b	3,19E-02	0,50
P61916	SRP14_HUMAN Signal recognition particle 14 kDa protein	2,68E-02	0,50
Q9UBT2	NCF4_HUMAN Neutrophil cytosol factor 4	3,52E-02	0,50
P14866	CNN3_HUMAN Calponin-3	1,38E-02	0,49
Q16698	SYEP_HUMAN Bifunctional glutamate/proline--tRNA ligase	1,47E-03	0,49
Q8IWE2	DCD_HUMAN Dermcidin	2,19E-02	0,48
P25786	GNS_HUMAN N-acetylglucosamine-6-sulfatase	2,29E-02	0,48
Q8N163	NTF2_HUMAN Nuclear transport factor 2	2,63E-02	0,47
P52597	NB5R3_HUMAN NADH-cytochrome b5 reductase 3	1,33E-02	0,47
P20073	SYWC_HUMAN Tryptophan--tRNA ligase, cytoplasmic	4,77E-03	0,46

Q6UB35	AP2A1_HUMAN AP-2 complex subunit alpha-1	2,05E-02	0,45
Q15417	TPM4_HUMAN Tropomyosin alpha-4 chain	6,56E-03	0,45
P0DP25	ACON_HUMAN Aconitate hydratase	4,02E-02	0,43
P13929	PRRC1_HUMAN Protein PRRC1	2,54E-02	0,43
P26641	MYG1_HUMAN UPF0160 protein MYG1, mitochondrial	1,83E-02	0,42
Q76M96	SYAC_HUMAN Alanine--tRNA ligase, cytoplasmic	2,38E-02	0,42
P09972	SYCC_HUMAN Cysteine--tRNA ligase, cytoplasmic	5,31E-03	0,40
P12268	UGGG1_HUMAN UDP-glucose:glycoprotein glucosyltransferase 1	3,41E-02	0,40
P36873	TGM3_HUMAN Protein-glutamine gamma-glutamyltransferase E	1,98E-02	0,38
P14923	PSB3_HUMAN Proteasome subunit beta type-3	8,36E-03	0,37
Q9NR30	IF2B2_HUMAN Insulin-like growth factor 2 mRNA-binding protein 2	1,56E-02	0,36
P42167	GPX1_HUMAN Glutathione peroxidase 1	1,11E-02	0,36
P62266	DPYL2_HUMAN Dihydropyrimidinase-related protein 2	7,71E-03	0,34
Q9Y5M8	RS4X_HUMAN 40S ribosomal protein S4, X isoform	4,51E-02	0,34
P60903	ERAP1_HUMAN Endoplasmic reticulum aminopeptidase 1	1,04E-02	0,33
Q12931	CAV1_HUMAN Caveolin-1	3,29E-02	0,32
P49006	GLGB_HUMAN 1,4-alpha-glucan-branching enzyme	4,20E-02	0,32
P78417	PDLI7_HUMAN PDZ and LIM domain protein 7	7,82E-03	0,31
Q9H4A4	NOMO3_HUMAN Nodal modulator 3	7,21E-04	0,30
Q1KMD3	ETHE1_HUMAN Persulfide dioxygenase ETHE1, mitochondrial	2,92E-02	0,30
P39019	TXLNA_HUMAN Alpha-taxilin	2,40E-03	0,29
P61970	RFIP5_HUMAN Rab11 family-interacting protein 5	3,18E-02	0,29
P43353	PAI1_HUMAN Plasminogen activator inhibitor 1	3,70E-02	0,29
P54709	CSPG4_HUMAN Chondroitin sulfate proteoglycan 4	4,30E-02	0,28
P35080	ILF2_HUMAN Interleukin enhancer-binding factor 2	2,80E-02	0,27
Q9Y2Q5	GGCT_HUMAN Gamma-glutamylcyclotransferase	3,47E-02	0,27
P15144	ACOT1_HUMAN Acyl-coenzyme A thioesterase 1	7,48E-03	0,26
Q15758	NIBA2_HUMAN Protein Niban 2	3,25E-02	0,26

Q06830	FKBP9_HUMAN Peptidyl-prolyl cis-trans isomerase FKBP9	7,56E-03	0,26
Q99832	SYIC_HUMAN Isoleucine--tRNA ligase, cytoplasmic	3,60E-02	0,26
Q14677	MYH10_HUMAN Myosin-10	1,54E-02	0,24
P55809	SPEE_HUMAN Spermidine synthase	4,41E-02	0,24
P01834	IQGA1_HUMAN Ras GTPase-activating-like protein IQGAP1	2,42E-03	0,24
P20962	CRTAP_HUMAN Cartilage-associated protein	4,39E-02	0,23
Q15084	SULF1_HUMAN Extracellular sulfatase Sulf-1	2,10E-02	0,23
P55036	HDGF_HUMAN Hepatoma-derived growth factor	2,40E-02	0,22
O15511	ZO1_HUMAN Tight junction protein ZO-1	3,03E-04	0,21
Q16891	PICAL_HUMAN Phosphatidylinositol-binding clathrin assembly protein	3,96E-02	0,21
P06132	RS16_HUMAN 40S ribosomal protein S16	4,27E-02	0,21
Q92882	AMPN_HUMAN Aminopeptidase N	4,34E-02	0,20
P09769	NCPR_HUMAN NADPH--cytochrome P450 reductase	1,16E-02	0,20
Q02750	PTGR1_HUMAN Prostaglandin reductase 1	4,20E-02	0,20
P20700	RL24_HUMAN 60S ribosomal protein L24	3,18E-02	0,19
P62258	MACF1_HUMAN Microtubule-actin cross-linking factor 1, isoforms 1/2/3/5	4,88E-02	0,19
Q9BQE5	GLSK_HUMAN Glutaminase kidney isoform, mitochondrial	2,97E-02	0,19
P63010	PIPNB_HUMAN Phosphatidylinositol transfer protein beta isoform	1,93E-02	0,19
P48506	FBRL_HUMAN rRNA 2-	9,49E-04	0,17
P31942	CAD13_HUMAN Cadherin-13	3,54E-02	0,17
Q9NQW7	AL1L1_HUMAN Cytosolic 10-formyltetrahydrofolate dehydrogenase	3,51E-02	0,17
P24534	PABP4_HUMAN Polyadenylate-binding protein 4	4,32E-02	0,16
Q9BYT8	CO6A3_HUMAN Collagen alpha-3(VI) chain	4,97E-02	0,16
Q6NYC8	ARHG2_HUMAN Rho guanine nucleotide exchange factor 2	1,02E-02	0,16
P12235	SERC_HUMAN Phosphoserine aminotransferase	2,01E-03	0,15
P05362	RALA_HUMAN Ras-related protein Ral-A	1,61E-02	0,15
P40763	COX41_HUMAN Cytochrome c oxidase subunit 4 isoform 1, mitochondrial	1,27E-02	0,15
P40123	TPM1_HUMAN Tropomyosin alpha-1 chain	1,53E-04	0,15

P04181	TARA_HUMAN TRIO and F-actin-binding protein	1,18E-02	0,15
P52790	SEPR_HUMAN Prolyl endopeptidase FAP	4,88E-02	0,15
P54920	NDUV1_HUMAN NADH dehydrogenase [ubiquinone] flavoprotein 1, mitochondrial	1,51E-02	0,14
P20908	GDIA_HUMAN Rab GDP dissociation inhibitor alpha	4,33E-02	0,14
P21980	EIF2A_HUMAN Eukaryotic translation initiation factor 2A	8,99E-03	0,14
Q13724	PRS6A_HUMAN 26S proteasome regulatory subunit 6A	5,90E-03	0,14
P29401	SAMH1_HUMAN Deoxynucleoside triphosphate triphosphohydrolase SAMHD1	4,57E-02	0,14
Q96CX2	NEXN_HUMAN Nexilin	7,34E-03	0,13
O00154	EHD1_HUMAN EH domain-containing protein 1	9,78E-03	0,13
Q8WXF1	PFKAL_HUMAN ATP-dependent 6-phosphofructokinase, liver type	2,57E-02	0,13
P31946	NUCB1_HUMAN Nucleobindin-1	9,86E-03	0,13
Q16270	AT1B3_HUMAN Sodium/potassium-transporting ATPase subunit beta-3	2,92E-02	0,13
O15269	NNMT_HUMAN Nicotinamide N-methyltransferase	3,69E-02	0,12
P11413	ASAH1_HUMAN Acid ceramidase	8,32E-04	0,12
P16930	NCF2_HUMAN Neutrophil cytosol factor 2	3,21E-02	0,12
P53814	ASSY_HUMAN Argininosuccinate synthase	1,36E-02	0,11
P69849	CRIP2_HUMAN Cysteine-rich protein 2	4,65E-02	0,11
P62424	APOE_HUMAN Apolipoprotein E	5,29E-04	0,11
Q13162	GOGA3_HUMAN Golgin subfamily A member 3	2,17E-02	0,10
P07195	PCKGM_HUMAN Phosphoenolpyruvate carboxykinase [GTP], mitochondrial	2,01E-02	0,10
P12081	GBG12_HUMAN Guanine nucleotide-binding protein G(I)/G(S)/G(	4,37E-02	0,10
P16435	PAWR_HUMAN PRKC apoptosis WT1 regulator protein	9,32E-03	0,09
Q16881	LTOR1_HUMAN Ragulator complex protein LAMTOR1	4,08E-02	0,09
P80303	TPP1_HUMAN Tripeptidyl-peptidase 1	1,35E-02	0,09
Q9BY89	BAG2_HUMAN BAG family molecular chaperone regulator 2	2,00E-02	0,09
P84077	SRP72_HUMAN Signal recognition particle subunit SRP72	3,20E-02	0,08
Q9ULE4	AP3S1_HUMAN AP-3 complex subunit sigma-1	8,03E-05	0,08
O00203	CTND1_HUMAN Catenin delta-1	3,60E-05	0,07

P07384	THTM_HUMAN 3-mercaptopyruvate sulfurtransferase	6,49E-04	0,07
P07942	AL1L2_HUMAN Mitochondrial 10-formyltetrahydrofolate dehydrogenase	1,83E-02	0,07
P04843	EMAL4_HUMAN Echinoderm microtubule-associated protein-like 4	4,43E-02	0,07
Q13501	UFD1_HUMAN Ubiquitin recognition factor in ER-associated degradation protein 1	1,51E-03	0,07
O94905	CCN1_HUMAN CCN family member 1	5,55E-03	0,06
O60568	YBOX3_HUMAN Y-box-binding protein 3	3,42E-04	0,06
P27824	ASNS_HUMAN Asparagine synthetase [glutamine-hydrolyzing]	7,01E-06	0,06
P17174	SUN2_HUMAN SUN domain-containing protein 2	8,92E-04	0,06
O95573	RAB23_HUMAN Ras-related protein Rab-23	3,23E-02	0,05
O43914	KCC2D_HUMAN Calcium/calmodulin-dependent protein kinase type II subunit delta	4,48E-02	0,05
Q9H2U2	UBC12_HUMAN NEDD8-conjugating enzyme Ubc12	1,81E-03	0,04
Q01813	CO6A1_HUMAN Collagen alpha-1(VI) chain	1,13E-03	0,04
P46781	GNA11_HUMAN Guanine nucleotide-binding protein subunit alpha-11	1,72E-03	0,04
Q07866	CSPG2_HUMAN Versican core protein	1,71E-02	0,04
Q96TA1	PDLI1_HUMAN PDZ and LIM domain protein 1	7,63E-04	0,04
P35222	PA1B2_HUMAN Platelet-activating factor acetylhydrolase IB subunit beta	5,37E-09	0,03
Q05682	FBLN2_HUMAN Fibulin-2	1,09E-03	0,03
B1ANS9	LETM1_HUMAN Mitochondrial proton/calcium exchanger protein	7,98E-05	0,03
